# Small Molecule Liver X Receptor Modulator GAC0001E5 Targets Mechanisms of Endocrine Resistance in Estrogen Receptor-Positive Breast Cancer Cells

**DOI:** 10.3390/biom16060856

**Published:** 2026-06-11

**Authors:** Shinjini Basu, Asitha Premaratne, Scott Widmann, Esther A. Olaleye, Chin-Yo Lin

**Affiliations:** 1 Center for Nuclear Receptors and Cell Signaling, Department of Biology and Biochemistry, University of Houston, Houston, TX 77004, USA; 2 Center for Biomedical and Health Research, Texas Southern University, Houston, TX 77004, USA; 3 Biomolecular Research and Advanced Computing Center, Texas Southern University, Houston, TX 77004, USA

**Keywords:** breast cancer, liver X receptor, estrogen receptor, endocrine resistance

## Abstract

Endocrine therapy is an effective and common treatment strategy for estrogen receptor (ER)-positive breast cancers. However, the development of endocrine resistance, through genetic mutations and epigenetic alterations, in about 40% of treated patients remains a significant therapeutic challenge. Liver X receptors (LXRs) are nuclear receptors that regulate lipid metabolism and cholesterol homeostasis and have been implicated in metabolic reprogramming in breast cancers and other malignancies. We previously identified a novel LXR ligand GAC0001E5 (1E5), with potent antiproliferative activity across breast cancer subtypes. Here, we investigate its mechanisms of action in responsive (MCF-7) and endocrine-resistant (MCF-7-TamR) ER-positive breast cancer cells. Treatment with 1E5 resulted in the downregulation of LXR and its target genes, and significantly reduced ERα expression and the expression of ER-responsive genes. Aberrant expression of androgen receptor (AR) and human epidermal growth factor receptor 2 (HER2), both implicated in endocrine resistance, were downregulated following 1E5 treatment. siRNA-mediated knockdown of LXR expression only partially recapitulated the actions of 1E5, suggesting the involvement of LXR-dependent and independent mechanisms. Collectively, these findings reveal potential crosstalk between LXR and the genetic and epigenetic regulation of pathways involved in endocrine response and alternative signaling mechanisms, highlighting potential targets in endocrine-resistant breast cancer.

## 1. Introduction

Breast cancer is one of the leading causes of cancer-related mortality among women worldwide [1]. Although advances in screening and endocrine therapies have greatly improved patient outcomes, endocrine resistance remains a major clinical challenge, necessitating further investigation into resistant mechanisms and alternative therapeutic approaches.

Hormone receptor-positive breast cancer accounts for approximately 70% of all breast cancer cases [2]. Estrogen receptor α (ERα) serves as the primary oncogenic driver by promoting aberrant cell proliferation and survival. Endocrine therapies targeting ER signaling, including selective estrogen receptor modulators (SERMs), aromatase inhibitors, and selective estrogen receptor degraders (SERDs), have long represented the standard of care for this subtype, with considerable success [3]. However, accumulating evidence indicates that genetic and epigenetic alterations contribute to endocrine resistance and disease recurrence in a significant subset of patients [4,5]. Mutations in the ERα ligand-binding domain, gene rearrangements or fusions, ERα amplification, and epigenetic reprogramming have all been linked to endocrine resistance [6]. Additional compensatory mechanisms, including aberrant expression of androgen receptor (AR) and human epidermal growth factor receptor 2 (HER2), as well as metabolic reprogramming, have also been implicated [7,8,9].

Liver X receptors (LXRs) are nuclear receptors, functioning as ligand-dependent transcription factors that regulate genes involved in lipid metabolism and cholesterol homeostasis. LXR overexpression has been reported in various cancer types, indicating a potential role in disease progression [10,11]. In breast cancer, LXR signaling has been associated with altered proliferative signaling and metabolism, with LXR expression correlating with hormone receptor status and disease progression [12]. Notably, LXR target gene expression and LXRβ levels have been reported to be elevated in malignant breast tissue compared to non-cancerous mammary tissue [13,14]. Previous studies have shown that LXR ligands exert antiproliferative effects across different breast cancer subtypes [15,16]. Both LXR agonists and inverse agonists have been studied in various cancer models, although agonists have been more extensively characterized and shown to adversely affect liver and circulating lipid levels while inverse agonists were lipid neutral [17,18,19,20]. Through a focused library screen of potential LXR ligands for basic and translational cancer research, our lab previously identified the small molecule GAC0001E5 (1E5), an LXR inverse agonist and degrader, with potent antiproliferative effects in a variety of breast cancer cells [14,21]. In this study, we investigate the mechanisms of action of 1E5 in cell models of tamoxifen-sensitive and tamoxifen-resistant ER-positive breast cancer.

## 2. Materials and Methods

### 2.1. Cell Culture and Treatments

Human ER-positive breast cancer cell lines MCF-7 (HTB-22) and T-47D (HTB-133) were purchased from American Type Culture Collection (ATCC) (Manassas, VA, USA). Both cell lines are representative luminal A breast cancer models characterized by high expression of estrogen receptor α (ERα) and low expression of human epidermal growth factor 2 (HER2). MCF-7 cells are widely used as a hormone-responsive luminal ER-positive breast cancer model, while T-47D cells serve as an additional ER-positive model to support experimental reproducibility in this study. MCF-7-TamR cells were generated in Dr. Tasneem Bawa-Khalfe’s lab (University of Houston, Houston, TX, USA) by treating MCF-7 cells with 1 μM 4-Hydroxytamoxifen (4-OHT) (Selleckchem, Houston, TX, USA, #S7827) for over six months. Subsequently, the cells were regularly treated with 1 μM 4-OHT to maintain endocrine resistance. In this study, MCF-7-TamR cells were used as a model of acquired endocrine resistance in breast cancer, with prior reports of increased expression of androgen receptor (AR) compared to parental MCF-7 cells [22].

Cell culture media and fetal bovine serum (FBS) were purchased from Thermo Fisher Scientific Inc. (Waltham, MA, USA). MCF-7 and MCF-7-TamR cells were cultured in Dulbecco’s Modified Eagle Medium (DMEM) (Gibco, Thermo Fisher Scientific Inc., Waltham, MA, USA, #12430054). T-47D cells were cultured in Roswell Park Memorial Institute Medium (RPMI-1640) (Gibco, #11875093). Media were supplemented with 10% FBS (Gibco, #A5670701). Cells were incubated at 37 °C in 5% CO_2_.

DMSO was obtained from VWR (Radnor, PA, USA, #97063136). LXR agonist GW3965 was purchased from Cayman Chemicals (Ann Arbor, MI, USA, #10054). Novel LXR inverse agonist GAC0001E5 was synthesized by OTAVA Chemicals (Concord, ON, Canada). LXR inverse agonists SR9238 and SR9243 were synthesized by Cayman Chemicals (Ann Arbor, MI, USA, #18771, #18420). These compounds were selected based on their established roles as LXR modulators in prior studies.

### 2.2. Real-Time Quantitative PCR (qRT-PCR)

Cells were seeded in 6-well plates at 2 × 10^5^ cells/well and treated after 24 h with vehicle DMSO, agonist GW3965, and inverse agonist 1E5 at 10 μM. Additional experiments included SR9238 and SR9243 also at 10 μM. After 48 h of incubation, RNA was extracted using RNeasy Mini Kit (Qiagen, Germantown, MD, USA, #74104). cDNA was prepared using 1 μg of RNA with iScript cDNA synthesis kit (Bio-Rad, Hercules, CA, USA, #1725035). qRT-PCR was performed with PowerUp^TM^ SYBR Green Master Mix (Applied Biosystems, Foster City, CA, USA, #A25742) on a 7500 Fast Real-Time PCR system (Applied Biosystems, Foster City, CA, USA, #4481192). Three biological replicates were conducted for each cell line. Relative fold-change, compared to vehicle, was calculated using the ΔΔCt method. qPCR data were presented as descriptive relative expression normalized to vehicle-treated samples. Primers were purchased from PrimerQuest^TM^ (Integrated DNA Technologies, Coralville, IA, USA) and are listed in Supplementary Table S1.

### 2.3. Western Blotting

Cells were seeded in 6-well plates at 2 × 10^5^ cells/well and treated with DMSO, GW3965, 1E5, SR9238, and SR9243 (10 μM) for 48 h. Western blotting was performed using standard procedures. Cell lysates were prepared in RIPA buffer containing protease inhibitors (Roche Diagnostics, Indianapolis, IN, USA, #11836170001), resolved by SDS-PAGE and transferred to PVDF membranes. Membranes were probed with primary antibodies against β-actin (Millipore, Burlington, MA, USA, #A2228), LXRβ (R&D Systems, Minneapolis, MN, USA, #PP-K8917), ERα (Cell Signaling Technology, Danvers, MA, USA, #8644), AR (Invitrogen, Carlsbad, CA, USA, #MA5-13426), HER2 (Cell Signaling Technology, Danvers, MA, USA, #4290), and PARP1 (Cell Signaling Technology, Danvers, MA, USA, #9542), followed by HRP-conjugated secondary antibodies (Cell Signaling Technology, Danvers, MA, USA, #7074, #7076). Membranes were visualized using Clarity^TM^ Western ECL (Bio-Rad, Hercules, CA, USA, #1705061) in the LI-COR Odyssey Fc system (LI-COR Biosciences, Lincoln, NE, USA, #OFC-0842). Western blot band intensities were quantified by densitometric analysis using ImageJ software v1.54 (National Institutes of Health, Bethesda, MD, USA). Protein expression levels were normalized to β-actin as a loading control, and results were plotted as relative fold change compared with control. All experiments were performed in biological triplicates.

### 2.4. MTS Assay

Cells were seeded in a 96-well plate at a seeding density of 1 × 10^4^ cells/well and treated with LXR ligands for 72 h. Cell proliferation was assessed using CellTiter 96 AQueous One Solution (MTS) (Promega, Madison, WI, USA, #G3581) and absorbance recorded at 490 nm using the SpectraMAX M5 plate reader (Molecular Devices, San Jose, CA, USA). Experiments were performed in biological triplicates with technical quadruplicates.

### 2.5. Trypan Blue Exclusion Assay

Cell viability was assessed through the trypan blue exclusion assay. Cells were seeded in 6-well plates at 2 × 10^5^ cells/well and treated with DMSO, GW3965, 1E5, SR9238, or SR9243 (10 μM) for 72 h. Cells were trypsinized, stained with trypan blue (Corning Inc., Corning, NY, USA, #25900CL), and viable cells were counted using a hemocytometer (MilliporeSigma, Burlington, MA, USA, #Z359629).

### 2.6. Clonogenic Assay

Cells were seeded in 6-well plates at 1 × 10^3^ cells/well. After 72 h, treatments (10 μM) were added and maintained for 48 h, with media and treatments replenished every 5 days until day 15. Colonies were fixed with 4% paraformaldehyde (MilliporeSigma, Burlington, MA, USA, #158127) and stained with 0.5% (*v*/*v*) crystal violet (MilliporeSigma, Burlington, MA, USA, #CO775) for 10 min. Excess stain was washed off with PBS (VWR, Radnor, PA, USA, #K813), and colonies counted.

### 2.7. siRNA Mediated Knockdown

Pooled siRNA and DharmaFECT transfection reagent (#T-2001-03) were purchased from Revvity Inc. (Waltham, MA, USA). Non-targeting scrambled control (#D-001800-10-50), LXRβ (#L-003412-02-0050), and LXRα (#L-003413-00-0050) siRNA were used. Stocks (100 μM) were diluted to 5 μM working solution. Cells were seeded in 6-well plates at 2 × 10^5^ cells/well. 24 h post-seeding, siRNA (10 μL) was mixed with 190 μL serum-free media (Gibco, Thermo Fisher Scientific Inc., Waltham, MA, USA, #31985062). Transfection reagent (5 μL) was added to 195 μL. After 5 min, siRNA and transfection reagent was combined, incubated 20 min, and added to each well containing culture media (Gibco, Thermo Fisher Scientific Inc., Waltham, MA, USA, #12430054). Knockdown efficiency was evaluated by qRT-PCR at 48 h post-transfection, and protein levels were assessed by Western blot at 96 h post-transfection.

### 2.8. Statistical Analysis

Statistical analyses and comparisons between treatment and control groups, as well as between sensitive and resistant cell lines, were conducted using Student’s *t*-tests, with paired or unpaired tests applied as appropriate. Data are presented as mean ± standard error of the mean (SEM). All experiments were performed with at least three independent biological replicates.

### 2.9. Reverse Docking and Nuclear Receptor Selectivity Analysis

The panel comprised all 48 human nuclear receptors, excluding the pseudogene NR1H5P/FXRβ. Primary target LXRβ used PDB 1UPV (T0901317 co-crystal). RORβ (NR1F2), lacking a human structure, used rat 1K4W (98.77% sequence identity in the LBD) directly as a template. Receptors without experimental human LBDs were docked using AlphaFold Protein Structure Database v6 models with docking boxes transferred from the nearest holo homolog by superposition [23]. The results were verified with P2Rank v2.4.2 (AlphaFold profile) [24]. Compound 1E5 and five positive controls (T0901317, estradiol, dexamethasone, rosiglitazone, calcitriol) were protonated at pH 7.4 with Molscrub v0.2.2 and converted to PDBQT with Meeko v0.7.1 (SDF input, default parameters) [25].

Receptors were prepared by Biopython v1.87 chain-A extraction with altloc stripping, PDBFixer heavy-atom repair, and Meeko conversion to PDBQT with --default_altloc A --allow_bad_res [26,27]. Structures that failed Meeko’s SanitizeMol pass were recovered by an Open Babel v3.1.0 connectivity round-trip on the clean input [28]. Rigid-receptor, flexible-ligand docking was performed with AutoDock Vina v1.2.6 (exhaustiveness 32, 20 output modes, 6 kcal/mol energy range, three fixed seeds of 101, 202, and 303 per receptor) [29]. Docking boxes were 22 Å cubes by default, 24 Å for PPAR and other extended-ligand pockets, 18 Å for TR and other small pockets, centered on the co-crystal ligand centroid or on the top P2Rank pocket. Per-receptor scores are reported as the best, median, and standard deviation of the rank-1 Vina score across seeds.

Baseline nuclear-receptor expression in parental MCF-7 was obtained from DepMap 24Q2 (MCF-7 = ACH-000019) [30]. All 48 NRs were mapped to their canonical HGNC symbols. A receptor was classified as expressed when TPM ≥ 1, following the Human Protein Atlas (HPA) convention [31]. Twenty-seven of 48 NRs passed the filter in parental MCF-7. The tamoxifen-resistant MCF-7 derivative was not used for filtering. Parental MCF-7 served as the baseline proxy. The filter was applied as a post-docking step for the Vina 48-NR rankings to yield a biologically grounded off-target panel.

## 3. Results

### 3.1. LXR Modulator GAC0001E5 (1E5) Inhibits Estrogen Receptor α (ERα)

**Figure 1:**
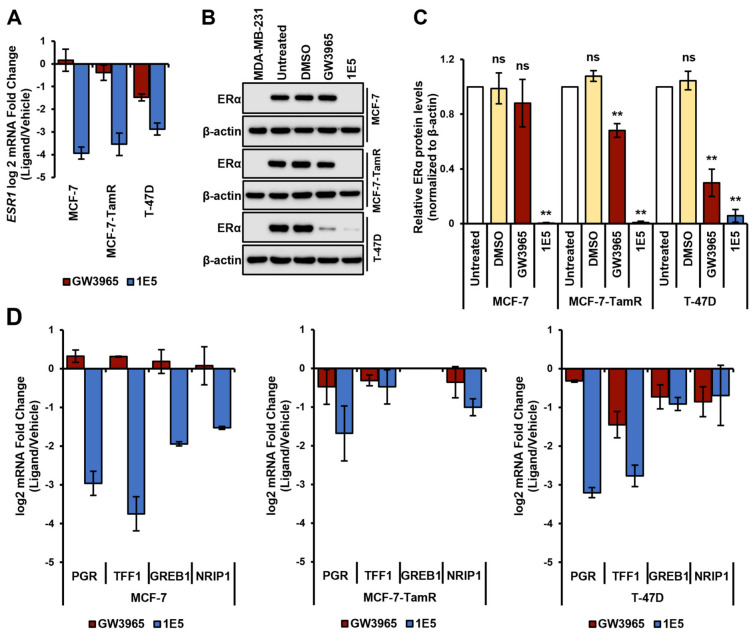
LXR modulator GAC0001E5 (1E5) downregulates Estrogen Receptor α (ERα) transcript and protein levels. (**A**) *ESR1* transcript levels were downregulated upon 1E5 treatment (10 µM) over 48 h. (**B**,**C**) ERα protein levels were significantly reduced upon 1E5 treatment over 48 h. No significant difference was observed between untreated and DMSO-treated cells. MDA-MB-231 cells were included as negative control. (**D**) Known ER target genes such as *PGR*, *TFF1*, *GREB1*, *NRIP1* were downregulated with 1E5 treatment, with more prominent downregulation in tamoxifen-sensitive cell lines. Data is presented as mean ± SEM from three independent biological replicates. Statistical significance was calculated using Student’s *t*-test (two-tailed, two-sample equal variance). Asterisks indicate statistical significance where ns *p* > 0.05, ** *p* < 0.01.

Previous studies have shown that the LXR modulator 1E5 exhibits potent antiproliferative effects in pancreatic and breast cancer models [14,21,32]. However, in ER-positive cells, its mechanisms of action remain unclear. GW3965 was included as a well-characterized synthetic LXR agonist and reference compound to enable direct comparison of LXR activation versus LXR inverse agonism mediated by 1E5. To determine whether LXR modulation by 1E5 affects ERα signaling, MCF-7, MCF-7-TamR, and T-47D cells were treated with the vehicle DMSO, LXR agonist GW3965 (10 μM), or 1E5 (10 μM) for 48 h. Treatment with 1E5 resulted in a marked reduction in ERα transcript levels (*ESR1*) across all three cell lines, whereas GW3965 reduced *ESR1* expression only in T-47D cells (Figure 1A). Western blot analysis confirmed that 1E5 significantly reduced ERα protein levels in both tamoxifen-sensitive and tamoxifen-resistant cells, while GW3965 reduced ERα protein expression only in MCF-7-TamR and T-47D cells (Figure 1B,C). No significant differences were observed between untreated and vehicle DMSO-treated controls. ER-negative MDA-MB-231 cells served as a negative control (Figure 1B, well 1).

ERα drives proliferative signaling in breast cancer by regulating downstream target genes, including those encoding progesterone receptor (*PGR*), trefoil factor 1 (*TFF1*), growth regulating estrogen receptor binding 1 (*GREB1*), and nuclear receptor interacting protein 1 (*NRIP1*) [33,34,35]. Therefore, to determine whether 1E5 affects ERα-dependent transcription, the expression of these established ER target genes was examined. Treatment with 1E5 reduced expression of all tested ER target genes in both tamoxifen-sensitive and tamoxifen-resistant models (Figure 1D). The effect was more pronounced in tamoxifen-sensitive MCF-7 and T-47D cells. In MCF-7-TamR cells, *GREB1* transcript levels were low or inconsistently detected, consistent with prior reports of epigenetic silencing in resistant models [36]. In contrast, GW3965 reduced ER target gene expression mainly in T-47D cells. These results indicate that 1E5 broadly disrupts ERα signaling in ER-positive breast cancer cells.

### 3.2. 1E5 Suppresses Expression of Androgen Receptor (AR)

**Figure 2:**
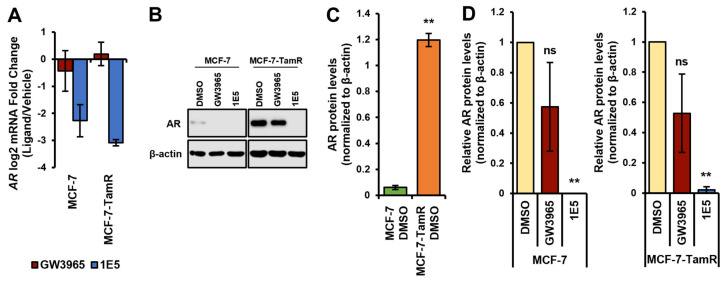
LXR inverse agonist 1E5 downregulates Androgen Receptor (AR) transcript and protein levels. (**A**) *AR* transcript levels were downregulated upon 1E5 treatment (10 µM). (**B**) Western blot for AR demonstrated greater expression in tamoxifen-resistant MCF-7-TamR compared to tamoxifen-sensitive MCF-7. (**C**) Differences in expression of AR in MCF-7-TamR vs. MCF-7 was found to be statistically significant. (**D**) AR protein levels were significantly reduced upon 1E5 treatment in both cell lines. Data is presented as mean ± SEM from three independent biological replicates. Statistical significance for between-cell-line comparison (MCF-7 vs. MCF-7-TamR) was calculated using Student’s *t*-test (two-tailed, two-sample unequal variance), and within cell-line comparison (treatment vs. vehicle) was calculated using Student’s *t*-test (two-tailed, two-sample equal variance). Asterisks indicate statistical significance where ns *p* > 0.05, ** *p* < 0.01.

Endocrine resistance in breast cancer can involve upregulation of alternative signaling pathways, including the androgen receptor (AR) and human epidermal growth factor receptor 2 (HER2). Overexpression of AR has been suggested to enable breast cancer cells to circumvent ER dependence in endocrine-resistant models [37]. To examine whether 1E5 affects AR expression, MCF-7 and MCF-7-TamR cells were treated with the vehicle DMSO, GW3965 (10 µM), or 1E5 (10 µM) for 48 h. Treatment with 1E5 downregulated *AR* transcripts in both cell lines, whereas GW3965 had minimal effect (Figure 2A). Canonical AR target genes, commonly studied in prostate cancer models, were undetectable or inconsistently expressed. 

Western blot analysis revealed markedly higher AR protein expression in DMSO-treated MCF-7-TamR compared to MCF-7 cells, consistent with previous reports of aberrantly elevated AR expression [38]. Treatment with 1E5 significantly reduced AR protein in MCF-7-TamR, while GW3965 did not produce a comparable effect (Figure 2B–D). Thus, 1E5 may attenuate proliferative signaling by downregulating AR, thereby disrupting a mechanism previously associated with endocrine resistance.

### 3.3. 1E5 Disrupts Expression of Human Epidermal Growth Factor Receptor 2 (HER2)

**Figure 3:**
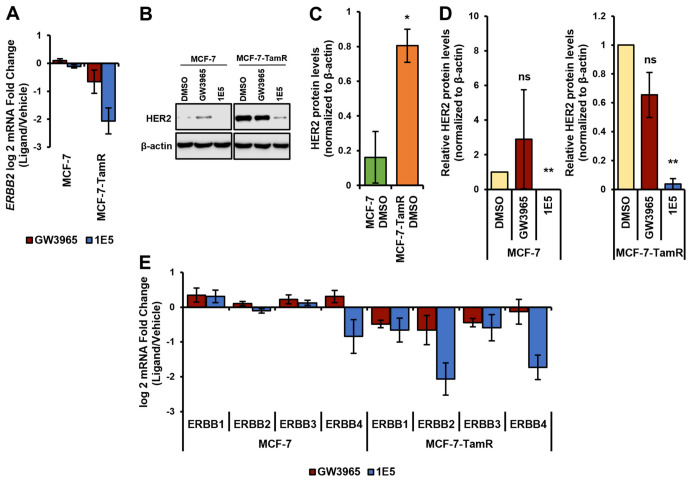
1E5 downregulates HER2 transcript and protein levels. (**A**) *ERBB2* (HER2) transcript levels were downregulated upon treatment with 1E5 (10 µM). (**B**,**C**) Western blot analysis shows reduction in HER2 protein upon 1E5 treatment, with higher baseline HER2 expression in tamoxifen-resistant MCF-7-TamR compared to tamoxifen-sensitive MCF-7. (**D**) HER2 protein levels were significantly reduced with 1E5 treatment in both cell lines. (**E**) Transcript levels of *ERBB*1-4 were reduced upon 1E5 treatment in MCF-7-TamR. Data is presented as mean ± SEM from three independent biological replicates. Statistical significance for between-cell-line comparison (MCF-7 vs. MCF-7-TamR) was calculated using Student’s *t*-test (two-tailed, two-sample unequal variance), and within cell-line comparison (treatment vs. vehicle) was calculated using Student’s *t*-test (two-tailed, two-sample equal variance). Asterisks indicate statistical significance where ns *p* > 0.05, * *p* < 0.05, ** *p* < 0.01.

HER2 is frequently upregulated in certain breast cancers and contributes to proliferation and endocrine resistance [39]. In this study, to assess whether endocrine resistance is associated with alterations in HER2 signaling, expression levels were compared between endocrine-sensitive MCF-7 and endocrine-resistant MCF-7-TamR cells under identical treatment conditions. Comparative Western blot analysis on the same gel confirmed elevated HER2 protein in MCF-7-TamR compared to MCF-7, with 1E5 significantly reducing HER2 levels, while GW3965 showed inconsistent effects (Figure 3B–D). Additionally, quantitative PCR analysis showed pronounced downregulation of *ERBB2* transcript levels in MCF-7-TamR cells following 1E5 treatment, whereas GW3965 had only modest effects. However, neither treatment significantly affected *ERBB2* transcript levels in MCF-7 cells (Figure 3A). These results suggest that 1E5 modulates HER2-mediated proliferative signaling in endocrine-resistant cells. Relatedly, HER2 can dimerize with other HER family members, including EGFR (HER1), HER3, and HER4, amplifying proliferative signaling [40]. Moreover, co-expression of multiple HER receptors is associated with poor patient outcomes [41]. Treatment with 1E5 reduced transcript levels of *ERBB1* (EGFR), *ERBB2* (HER2), *ERBB3* (HER3), and *ERBB4* (HER4) in MCF-7-TamR cells, whereas GW3965 produced only modest changes. In MCF-7 cells, neither treatment markedly altered HER family gene expression (Figure 3E). Therefore, 1E5 may specifically disrupt compensatory HER-mediated proliferative signaling pathways that contribute to endocrine resistance.

### 3.4. 1E5 Promotes PARP1 Cleavage

**Figure 4:**
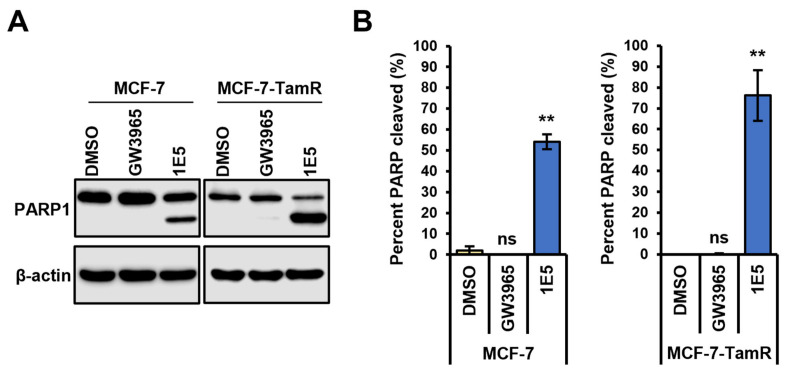
1E5 promotes PARP1 cleavage in ER-positive breast cancer. (**A**) Poly (ADP-ribose) polymerase 1 (PARP-1) cleavage represents apoptotic activation. Western blot for PARP exhibited full length PARP at 116 kDa. Cleaved product was observed at 89 kDa only in 1E5-treated samples. (**B**) Percent PARP cleavage was calculated, and significant cleavage was observed upon 1E5 treatment. Data is presented as mean ± SEM from three independent biological replicates. Statistical significance was calculated using Student’s *t*-test (two-tailed, two-sample equal variance). Asterisks indicate statistical significance where ns *p* > 0.05, ** *p* < 0.01.

In previous studies, 1E5 has been shown to induce oxidative stress in breast cancer through the accumulation of reactive oxygen species (ROS), which can trigger apoptosis [14,32]. ERα, AR, and HER2 signaling, in turn, promote cell survival, and co-inhibition of ER and HER2 or AR inhibition has been linked to pro-apoptotic effects [42,43]. Given that 1E5 suppresses these proliferative pathways, its effects on apoptosis in ER-positive breast cancer were next examined. Poly (ADP-ribose) polymerase (PARP1) is a DNA repair enzyme that facilitates cell survival. Cleavage of PARP1 is a well-established hallmark of apoptosis, reflecting loss of DNA repair function and activation of cell death pathways [44]. To evaluate whether 1E5 induces apoptosis, PARP1 cleavage was assessed in MCF-7 and MCF-7-TamR cells following treatment with DMSO, GW3965, and 1E5 for 48 h. Western blot analysis revealed full-length PARP1 (116 kDa) and the cleaved 89 kDa fragment (Figure 4A,B). In both cell lines, PARP1 cleavage was observed only with 1E5 treatment, whereas GW3965 did not induce detectable cleavage. These results indicate that 1E5 promotes apoptosis in ER-positive breast cancer cell models.

### 3.5. 1E5 Exhibits Potent Antiproliferative Effects Relative to SR9238 and SR9243

**Figure 5:**
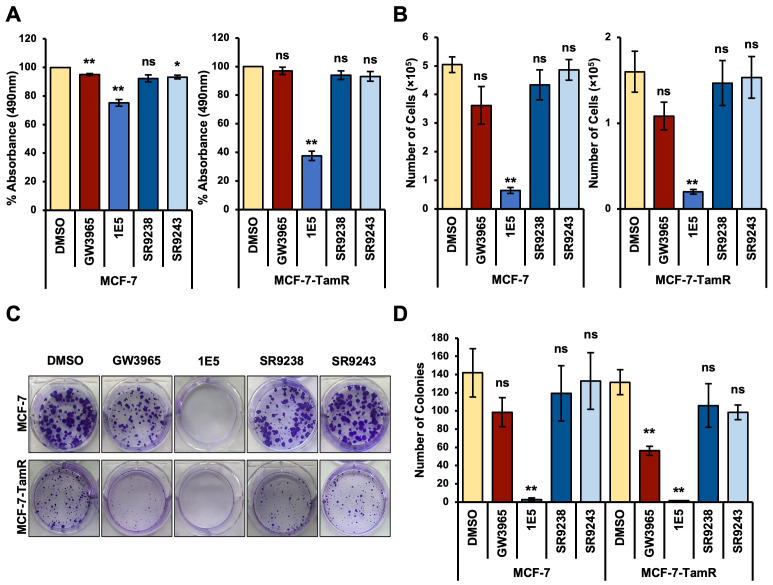
LXR inverse agonism is not sufficient to disrupt cell proliferation in ER-positive breast cancer. (**A**) MTS assay of breast cancer cells treated with LXR agonist GW3965 and inverse agonists GAC0001E5, SR9238, and SR9243 showed a greater reduction in metabolic activity upon 1E5 treatment compared to other LXR ligands. (**B**) Trypan blue exclusion assay corroborated the inhibitory effects, indicating that the antiproliferative effect was restricted to 1E5 treatment. (**C**,**D**) Clonogenic assay exhibited a similar reduction in number of colonies following 1E5 treatment. Data is presented as mean ± SEM from three independent biological replicates. Statistical significance was calculated using Student’s *t*-test (two-tailed, two-sample equal variance). Asterisks indicate statistical significance where ns *p* > 0.05, * *p* < 0.05, ** *p* < 0.01.

Initial characterization of 1E5 indicated its function as both an LXR inverse agonist and a potent LXR degrader. LXR agonists, such as GW3965, activate LXR-dependent downstream transcription, whereas inverse agonists, including SR9238 and SR9243, inhibit basal LXR activity. Both classes have been reported to exert antiproliferative effects in cancer models through mechanistically distinct pathways [15,19,45]. To determine whether the antiproliferative effects of 1E5 are primarily attributable to LXR inverse agonism, MCF-7 and MCF-7-TamR cells were treated with LXR agonist GW3965 or representative LXR inverse agonists SR9238 and SR9243, alongside 1E5 for comparative analysis of class-specific effects. Cell viability and proliferation were tested using MTS, trypan blue exclusion, and clonogenic assays. In MTS assays, 1E5 reduced metabolic activity compared to DMSO control, whereas GW3965, SR9238, and SR9243 produced only modest decreases (Figure 5A). Trypan blue exclusion confirmed that 1E5 significantly reduced the number of viable cells while the other ligands had minimal effects (Figure 5B). Clonogenic assays demonstrated that 1E5 strongly inhibited colony formation, whereas SR9238 and SR9243 had limited effects. GW3965 resulted in only a modest reduction in the number and size of colonies (Figure 5C,D). Together, these results show that 1E5 exerts greater antiproliferative activity compared to other LXR inverse agonists in ER-positive breast cancer cells.

### 3.6. 1E5 Selectively Disrupts LXRβ, ERα, AR, and HER2 Compared to SR9238 and SR9243

**Figure 6:**
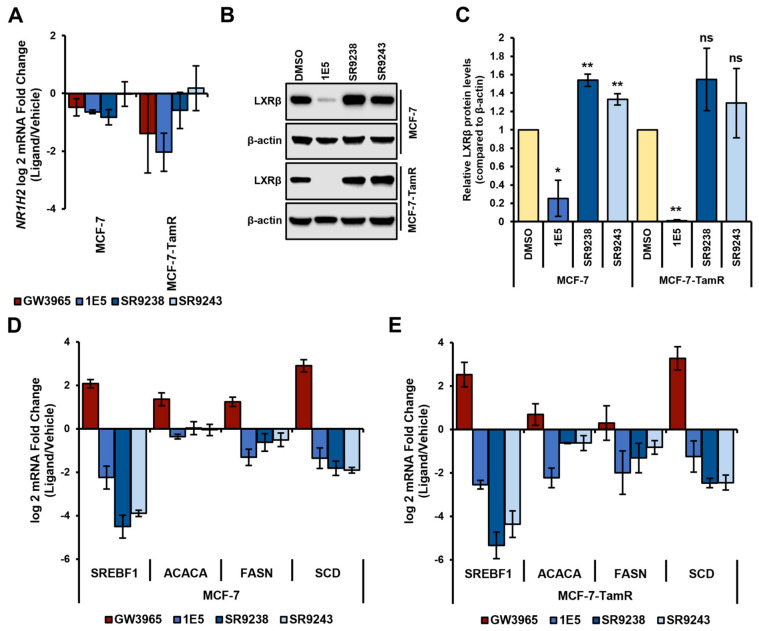
1E5 downregulates LXRβ expression and activity, whereas SR9238 and SR9243 solely downregulate downstream target genes. (**A**) *NR1H2* (LXRβ) transcript levels were reduced by GW3965, 1E5, and SR9238, but remain unchanged with SR9243. (**B**,**C**) LXRβ protein levels were significantly reduced upon 1E5 treatment, but upregulated following SR9238 and SR9243 treatment. (**D**,**E**) Expression of LXR target genes was downregulated upon treatment with all inverse agonists, 1E5, SR9238, and SR9243, in both tamoxifen-sensitive and tamoxifen-resistant cell lines. Data is presented as mean ± SEM from three independent biological replicates. Statistical significance was calculated using Student’s *t*-test (two-tailed, two-sample equal variance). Asterisks indicate statistical significance where ns *p* > 0.05, * *p* < 0.05, ** *p* < 0.01.

To determine whether differences in antiproliferative activity corresponded to LXR expression, LXRβ levels were assessed in both cell lines. LXRβ is the predominant isotype, expressed in most tissues, including ER-positive breast cancer. Treatments with 1E5 and SR9238 reduced LXRβ (*NR1H2*) transcript levels, whereas SR9243 had no effect (Figure 6A). Western blot analysis showed that 1E5 decreased LXRβ protein levels, consistent with transcript changes, while SR9238 unexpectedly increased LXRβ protein levels, and SR9243 had minimal effect (Figure 6B,C). These results highlight that the effect of 1E5 on LXRβ expression is mechanistically distinct and may contribute to its enhanced antiproliferative activity. To further assess whether differences in antiproliferative activity among LXR inverse agonists are reflected at the transcript level, expression of canonical LXR target genes was measured. Cells were treated with DMSO, 1E5, SR9238, or SR9243 at 10 μM for 48 h, with GW3965 as an agonist positive control for comparison. As expected, GW3965 upregulated LXR target genes in both cell lines, whereas all three inverse agonists reduced expression of *SREBF1*, *ACACA*, *FASN*, and *SCD* (Figure 6D,E). These results suggest that suppression of LXR target gene transcription alone is insufficient to elicit robust antiproliferative effects, distinguishing 1E5 from other inverse agonists.

**Figure 7:**
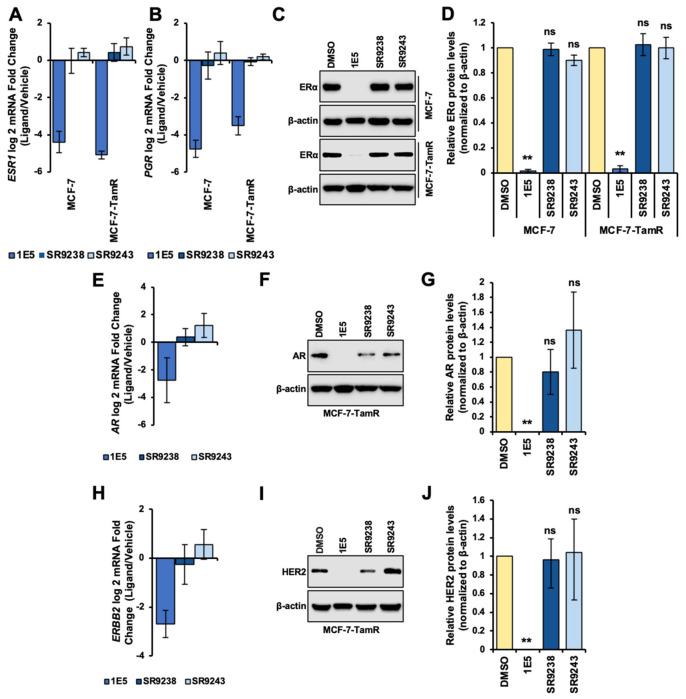
1E5 suppresses ERα, AR, and HER2 expression, whereas SR9238 and SR9243 have minimal effects. (**A**) *ESR1* transcript levels were downregulated following 1E5 treatment but remained unchanged with SR9238 and SR9243 treatment. (**B**) ER-target gene *PGR* transcript levels were similarly downregulated with 1E5 treatment. (**C**,**D**) ERα protein levels were reduced only with 1E5 treatment, with no significant reduction with SR9238 or SR9243. (**E**) *AR* transcript levels were downregulated upon 1E5 treatment in MCF-7-TamR cells. (**F**,**G**) AR protein levels were reduced following 1E5 treatment, whereas SR9238 and SR9243 had no significant effect. (**H**) *ERBB2* transcript levels were decreased upon treatment with 1E5, with minimal change observed with SR9238 or SR9243. (**I**,**J**) HER2 protein levels were reduced with 1E5 treatment, while SR9238 and SR9243 produced no significant change. Parental MCF-7 cells were excluded due to low AR and HER2 expression relative to MCF-7-TamR cells. β-actin blots were used as loading control, and the same image was used across multiple panels for illustrative purposes where appropriate for each cell line. Data is presented as mean ± SEM from three independent biological replicates. Statistical significance was calculated using Student’s *t*-test (two-tailed, two-sample equal variance). Asterisks indicate statistical significance where ns *p* > 0.05, ** *p* < 0.01.

Treatments with 1E5 also disrupted the expression of ERα, AR, and HER2 in endocrine-resistant breast cancer cells. *ESR1* transcript levels were prominently downregulated by 1E5, whereas SR9238 and SR9243 had no significant effect (Figure 7A). Western blotting confirmed that only 1E5 reduced ERα protein levels (Figure 7C,D). The ER target gene *PGR* showed a similar pattern, demonstrating that 1E5 suppresses ERα expression and downstream transcriptional activity (Figure 7B). In MCF-7-TamR cells, 1E5 also markedly reduced AR and HER2 (*ERBB2*) transcript and protein levels, whereas SR9238 and SR9243 showed minimal or inconsistent effects (Figure 7E–J). These results demonstrate that 1E5 uniquely downregulates LXRβ as well as ERα, AR, and HER2, indicating that its antiproliferative activity could extend beyond LXR inverse agonism alone.

### 3.7. LXR Knockdown Moderately Reduces Proliferation and ERα Signaling

**Figure 8:**
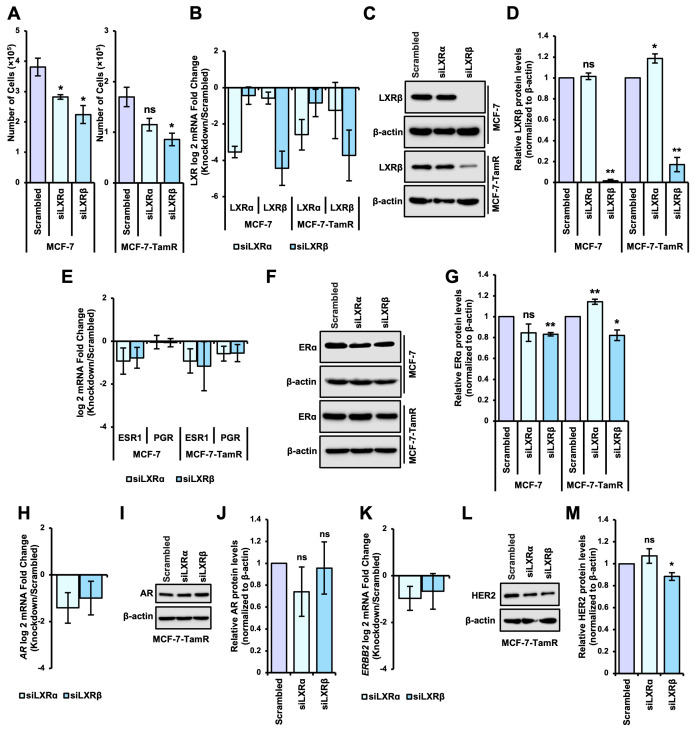
LXR knockdown modestly reduces cell proliferation and ERα expression but has limited effects on AR and HER2 in ER-positive breast cancer. (**A**) Trypan blue exclusion assay showed a significant reduction in cell proliferation upon LXR knockdown. (**B**) LXR transcript levels were downregulated following siRNA-mediated knockdown. (**C**,**D**) LXRβ protein levels were significantly reduced upon knockdown. (**E**) *ESR1* and *PGR* transcript levels showed modest reduction upon LXR depletion. (**F**,**G**) ERα protein levels showed slight reduction upon LXR knockdown. (**H**–**J**) *AR* transcript levels showed a small decrease whereas protein levels remained unchanged post knockdown. (**K**) *ERBB2* transcript levels showed slight reduction following LXR depletion. (**L**,**M**) HER2 protein expression was modestly inhibited upon LXRβ knockdown. β-actin blots were used as loading control and the same image was used across multiple panels for illustrative purposes where appropriate for each cell line. Data is presented as mean ± SEM from three independent biological replicates. Statistical significance was calculated using Student’s *t*-test (two-tailed, two-sample equal variance). Asterisks indicate statistical significance where ns *p* > 0.05, * *p* < 0.05, ** *p* < 0.01.

Since LXR inverse agonism did not fully recapitulate the antiproliferative effects of 1E5, we next investigated whether LXR depletion could influence cell proliferation and receptor signaling. MCF-7 and MCF-7-TamR cells were transfected with siRNAs targeting LXRα or LXRβ. Trypan blue exclusion assays revealed modest but statistically significant reductions in viable cell numbers following knockdown of either isotype in MCF-7 cells (Figure 8A). In MCF-7-TamR cells, only LXRβ knockdown decreased cell numbers. MTS assays showed no significant changes in metabolic activity. Knockdown efficiency was confirmed at the transcript and protein levels (Figure 8B–D). Both LXRα and LXRβ transcripts were effectively reduced relative to scrambled controls. LXRβ protein was markedly decreased, whereas LXRα protein remained low and inconsistently detected, consistent with its low expression in breast cancer [46].

To assess the effects of gene knockdown on ERα signaling, *ESR1* and ER target gene *PGR* transcript levels were measured following transfection. LXR knockdown led to modest reductions in *ESR1* expression, whereas *PGR* remained largely unchanged (Figure 8E). At the protein level, ERα showed a modest but statistically significant decrease following LXRβ knockdown (Figure 8F,G). Conversely, LXRα knockdown produced inconsistent results between cell lines. These results suggest that LXR, particularly LXRβ, may influence ERα expression and signaling through molecular crosstalk, although the effects are less pronounced than those observed with pharmacological inhibition by 1E5.

### 3.8. LXR Knockdown Partially Reduces Expression of AR and HER2

In tamoxifen-resistant MCF-7-TamR cells, which display elevated AR and HER2 expression, the impact of LXR depletion on these pathways was examined. LXR knockdown resulted in a modest reduction in AR transcript levels (Figure 8H). However, Western blot analysis revealed no significant change in AR protein level as compared to the scrambled control (Figure 8I,J). *ERBB2* (HER2) transcript levels showed a slight decrease following LXR knockdown, while Western blotting showed a modest but statistically significant reduction in HER2 following LXRβ depletion (Figure 8K–M). Taken together, these results signify that LXRβ may contribute to hormone and growth factor receptor signaling in tamoxifen-resistant breast cancer, with only modest effects on AR and HER2. The effects of LXR knockdown were less pronounced than those observed with pharmacological inhibition by 1E5, highlighting that the potent antiproliferative activity of 1E5 likely involves additional pleiotropic mechanisms, potentially including LXR-independent pathways.

## 4. Discussion

Small molecule LXR modulator 1E5 has previously been shown to exert potent antiproliferative effects in breast cancer [14,32]. However, the mechanisms underlying this activity in ER-positive breast cancer have remained unclear. In this study, we demonstrate that 1E5 significantly downregulates ERα, the principal oncogenic driver in this molecular subtype. Prior studies have shown that LXR ligands can suppress ER-positive breast cancer by disrupting ER-dependent signaling, supporting a role for LXR modulation in controlling oncogenic ER activity [16]. A major challenge in breast cancer treatment is the development of endocrine resistance, often driven by the activation of compensatory signaling pathways, including AR and HER2. The role of AR in endocrine resistance is context-dependent, with reports of both tumor-promoting and tumor-suppressive effects [7,47]. In some models, AR upregulation converts tamoxifen from an antagonist to an agonist and contributes to ER-dependent transcription [38,48]. AR loss has also been shown to inhibit growth and restore tamoxifen sensitivity in resistant models [7]. HER2 overexpression similarly sustains proliferative signaling and contributes to endocrine resistance, with HER2-targeted therapies restoring endocrine responsiveness [8,49]. In the present study, MCF-7-TamR cells represent an endocrine-resistant model that has previously been shown to exhibit increased AR expression compared to parental MCF-7 cells [22]. This may be associated with compensatory proliferative signaling and reduced ERα dependence in this cell line. Here, 1E5 coordinately suppressed ERα, AR, and HER2, as well as additional HER family members, suggesting broad disruption of compensatory proliferative signaling networks associated with endocrine resistance. While these effects were supported at the transcript level for multiple HER family members, the absence of protein-level validation represents a limitation of this study and would provide additional insight into downstream signaling effects. Overall, this suppression provides a mechanistic basis for the potent antiproliferative effects of 1E5. Moreover, 1E5 induced PARP1 cleavage, indicating activation of apoptotic pathways and supporting the model that 1E5 both disrupts oncogenic signaling and promotes cell death in ER-positive breast cancer.

1E5 has been characterized as both an LXR inverse agonist and an LXR “degrader”, capable of downregulating canonical LXR target genes as well as LXR expression itself [14,21,32]. To distinguish whether its antiproliferative effects arise from inverse agonism alone or from receptor depletion, we compared 1E5 with known LXR inverse agonists, SR9238 and SR9243. Although all three compounds effectively downregulated LXR target genes, only 1E5 elicited robust antiproliferative effects in both tamoxifen-sensitive and tamoxifen-resistant models. Notably, 1E5 uniquely reduced LXRβ expression, whereas the other inverse agonists appeared to stabilize LXRβ protein, suggesting that receptor depletion may contribute to the distinct activity of 1E5. Consistent with this, only 1E5 suppressed ERα, AR, and HER2 expression, indicating that suppression of LXR target genes through inverse agonism alone is insufficient to elicit a strong antiproliferative response. Although 1E5, SR9238, and SR9243 are all classified as LXR inverse agonists, structural differences among these ligands may influence their receptor interaction profiles and downstream transcription effects and potential molecular crosstalk. Previous studies have reported disruption of the His435-Trp457 interaction within the LXR ligand-binding pocket following SR9238 and SR9243 binding [50]. In contrast, this interaction appears to be maintained with 1E5 [21]. Additionally, differences in molecular size and associated steric properties may further contribute to the distinct ligand-mediated effects. Future studies investigating ligand-specific receptor conformations and interactions with additional receptors could further clarify the molecular basis underlying the differential effects observed with these ligands.

On the other hand, siRNA-mediated knockdown of both LXR isotypes resulted in only modest reductions in cell viability and limited effects on ERα, AR, and HER2 compared to pharmacological inhibition by 1E5. Although LXRβ knockdown modestly reduced HER2 protein levels, AR expression was largely unaffected despite small transcript level differences. These findings indicate that LXR-mediated molecular crosstalk may contribute to the genetic and epigenetic regulation of oncogenic signaling but does not fully account for the potent antiproliferative response of 1E5, suggesting involvement of additional mechanisms beyond receptor depletion alone.

To analyze the potential receptor selectivity profile of 1E5, reverse docking was performed against the human nuclear receptor superfamily, including the 48 canonical nuclear receptors. This analysis identified LXRα and LXRβ among the top-ranked predicted targets (Supplementary Table S2). Notably, while LXRα demonstrated strong predicted binding, expression data indicates that LXRβ is more abundant and may be more functionally relevant in this model system. In addition to LXR-dependent mechanisms, reverse docking also suggested potential interactions with other nuclear receptors such as retinoic acid receptor γ (RARγ), thereby suggesting off-target effects and LXR-independent mechanisms of action of 1E5. RARγ has previously been linked with tumorigenic effects in breast cancer models [51]. Additionally, retinoic acid signaling has been reported to interact with ERα, suggesting a potential crosstalk and indirect modulation independent of LXR activity [52]. In contrast to the possible interactions of 1E5 with other nuclear receptors, SR9238 and SR9243 have been reported to exhibit high selectivity for LXR with minimal off-target interactions [19,45]. Therefore, the broader effects of 1E5 may be associated with its wider nuclear receptor interaction profile. Further studies will be required to fully define the contribution of these interactions.

Taken together, the data indicates that the antiproliferative activity of 1E5 cannot be attributed solely to LXR inverse agonism or receptor depletion. LXR signaling is known to functionally interact with ERα, AR, and HER2 pathways [53,54,55,56,57]. Therefore, pharmacological disruption of LXR by 1E5 may induce coordinated suppression of known oncogenic signaling cascades while simultaneously triggering apoptosis. In addition, reverse docking analysis suggests potential LXR-independent mechanisms that may further regulate proliferative signaling pathways. Thus, this supports a broader multitargeted mechanism underlying the activity of 1E5.

## 5. Conclusions

This work provides novel mechanistic insights into the actions of LXR modulator 1E5 in ER-positive breast cancer. It exhibits potent antiproliferative activity by disrupting oncogenic signaling pathways and promoting PARP1 cleavage, indicating simultaneous inhibition of proliferation and induction of apoptosis. These effects may be mediated not only through LXR inverse agonism and receptor degradation but may also involve additional LXR-independent mechanisms, potentially including other genetic and epigenetic factors.

While these findings highlight the unique mechanism of action of 1E5, certain limitations, including the lack of in vivo models, remain. Additionally, evaluation of prolonged exposure and acquired resistance, as well as combination studies with other endocrine therapies, are needed to better define the precise molecular interactions targeted by 1E5. Although siRNA-mediated knockdown of LXR was performed, stable knockout approaches would further strengthen mechanistic interpretation. Reverse docking analysis demonstrates targeting of LXR while also indicating potential interactions with additional nuclear receptors. Therefore, pleiotropic effects, ligand promiscuity, and potential off-target or LXR-independent mechanisms cannot be fully ruled out. Transcriptomic, proteomic, and experimental validation of predicted molecular interactions could provide key mechanistic insights. Future research on receptor selectivity, ligand optimization, and downstream signaling will be important to further define the therapeutic potential of 1E5 and advance the development of related therapeutic strategies for breast cancer and other malignancies.

## Data Availability

The data presented in this study are available in the public domain: [DepMap] [https://depmap.org/portal/] [ACH-000019] (accessed on 19 April 2026).

## Supplementary Materials

The following supporting information can be downloaded at https://www.mdpi.com/article/10.3390/biom16060856/s1, Table S1: List of primers used in the study; Table S2: Reverse docking results for 1E5 with human nuclear receptor superfamily; Folder S3: Western blot images with key.
