# Supplementary material for: Small Molecule Liver X Receptor Modulator GAC0001E5 Targets Mechanisms of Endocrine Resistance in Estrogen Receptor-Positive Breast Cancer Cells
Source: Biomolecules. 2026 Jun 11;16(6):856. doi: 10.3390/biom16060856 (PMC13296967; doi:10.3390/biom16060856)
Supplement: Supplementary file 1 [file biomolecules-16-00856-s001.zip › Supplementary Table S1 - Primers.pdf]

Supplementary Table S1: List of primers used in the study

Primer ID	Primer Sequence
36B4-F	5′-GTGTTCGACAATGGCAGCAT-3′
36B4-R	5′-GACACCCTCCAGGAAGCGA-3′
SREBP1c-F	5′-GGAGGGGTAGGGCCAACGGCCT-3′
SREBP1c-R	5′-CATGTCTTCGAAAGTGCAATCC-3′
LXRα-F	5′-GGAGGTACAACCCTGGGAGT-3′
LXRα-R	5′-AGCAATGAGCAAGGCAAACT-3′
LXRβ-F	5′-ATCAAGAGGCCGCAGGACCA-3′
LXRβ-R	5′-AGGCGAAGACCTGCTCCGAG-3′
ACACA (ACC)-F	5′-GCAGGTCACACGTCTCTTTAT-3′
ACACA (ACC)-R	5′-CCAGCCTGTCATCCTCAATATC-3′
FASN-F	5′-ACAGGGACAACCTGGAGTTCT-3′
FASN-R	5′-CTGTGGTCCCACTTGATGAGT-3′
SCD1-F	5′-TTCAGAAACACATGCTGATCCTCATAA-3′
SCD1-R	5′- ATTAAGCACCACAGCATATCGCAAGAA -3′
ESR1-F	5′-CCCACTCAACAGCGTGTCTC-3′
ESR1-R	5′-CGTCGATTATCTGAATTTGGCCT-3′
PGR-F	5′-AGGACACCATAATGACAGCCT-3′
PGR-R	5′-ACCCGCCCTATCTCAACTACC-3′
TFF1-F	5′-CCCTCCCAGTGTGCAAATAAG-3′
TFF1-R	5′-GAACGGTGTCGTCGAAACAG-3′
GREB1-F	5′-CTGTACCACAGACGGGTTTTG-3′
GREB1-R	5′-TTCCGTGAAGTAACAGAAGCC-3′
NRIP1-F	5′-ATGCAGCAAAGCGGAAGAG-3′
NRIP1-R	5′-CCTTTAGGCACACTGTCAACC-3′
AR-F	5′-CCAGGGACCATGTTTTGCC-3′
AR-R	5′-CGAAGACGACAAGATGGACAA-3′
ERBB1-F	5′-AGGCACGAGTAACAAGCTCAC-3′
ERBB1-R	5′-ATGAGGACATAACCAGCCACC-3′
ERBB2-F	5′-TGCAGGGAAACCTGGAACTC-3′
ERBB2-R	5′-ACAGGGGTGGTATTGTTCAGC-3′
ERBB3-F	5′-GGTGATGGGGAACCTTGAGAT-3′
ERBB3-R	5′-CTGTCACTTCTCGAATCCACTG-3′
ERBB4-F	5′-GCCTCTGGAGAATTTACGCAT-3′
ERBB4-R	5′-GGGTTCCGAACAATATCTTGCC -3
