# Supplementary material for: Small Molecule Liver X Receptor Modulator GAC0001E5 Targets Mechanisms of Endocrine Resistance in Estrogen Receptor-Positive Breast Cancer Cells
Source: Biomolecules. 2026 Jun 11;16(6):856. doi: 10.3390/biom16060856 (PMC13296967; doi:10.3390/biom16060856)
Supplement: Supplementary file 1 [file biomolecules-16-00856-s001.zip › Western Blot Files/Figure 7 (ER, AR, HER2)/1_Key for Western Blot Images (Figure 7).docx]

All lanes correspond to the raw, uncropped gel images in this folder. Protein of interest and loading controls are visualized from the same blot for each cell line. Lanes shown in the manuscript figures are listed below.

Key for western blot (Figure 7 – ER, AR, HER2):

**Lane 1 –** DMSO-treatment

**Lane 2 –** 1E5-treatment

**Lane 3 –** SR9238-treatment

**Lane 4 –** SR9243-treatment

**Lane 5 –** KD95-treatment (not included in manuscript)

Example below.


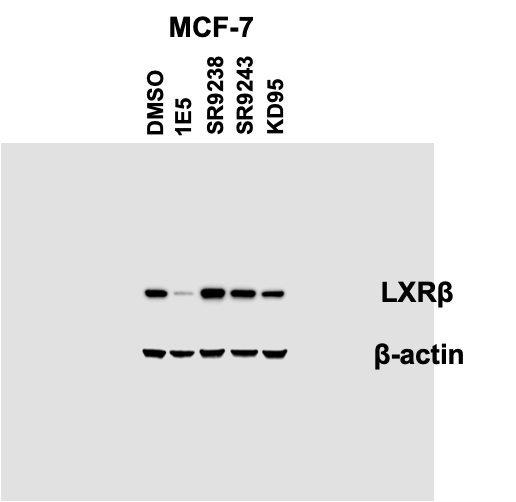


Note – The same blot was used to test expression of ER, AR and HER2 in Figure 7. The β-actin loading control corresponds to the same blot and lanes.
