# Supplementary figures and images for: Small Molecule Liver X Receptor Modulator GAC0001E5 Targets Mechanisms of Endocrine Resistance in Estrogen Receptor-Positive Breast Cancer Cells
Source: Biomolecules. 2026 Jun 11;16(6):856. doi: 10.3390/biom16060856 (PMC13296967; doi:10.3390/biom16060856)

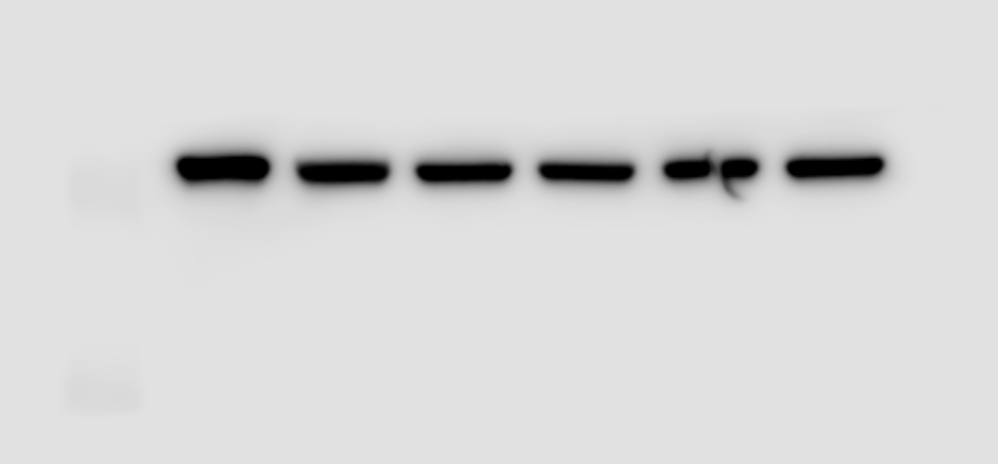

Supplement: Supplementary file 1 [file biomolecules-16-00856-s001.zip › Western Blot Files/Figure 1 (ER)/Fig1_MCF7TamR_Rep1_beta-actin_RAW.tif]

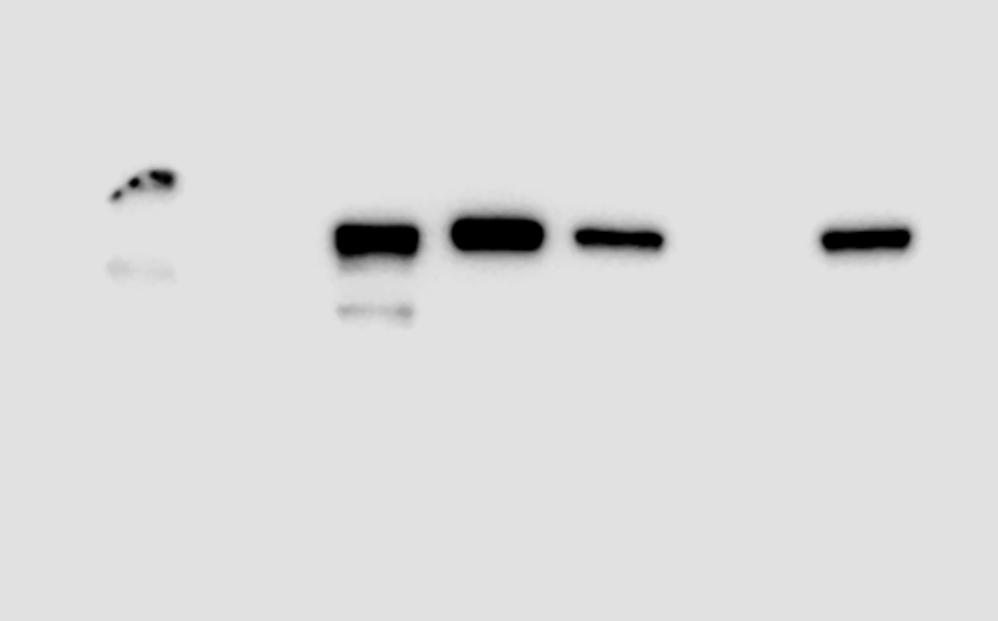

Supplement: Supplementary file 1 [file biomolecules-16-00856-s001.zip › Western Blot Files/Figure 1 (ER)/Fig1_MCF7TamR_Rep1_ERalpha_RAW.tif]

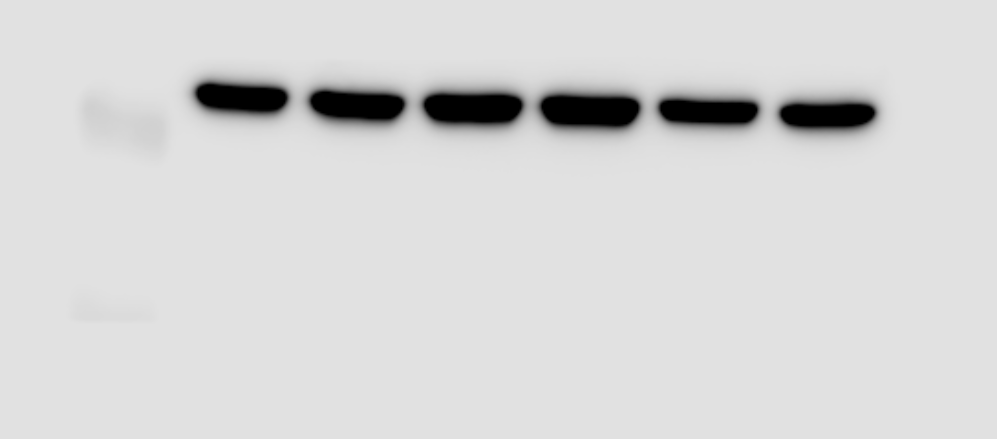

Supplement: Supplementary file 1 [file biomolecules-16-00856-s001.zip › Western Blot Files/Figure 1 (ER)/Fig1_MCF7TamR_Rep2_beta-actin_RAW.tif]

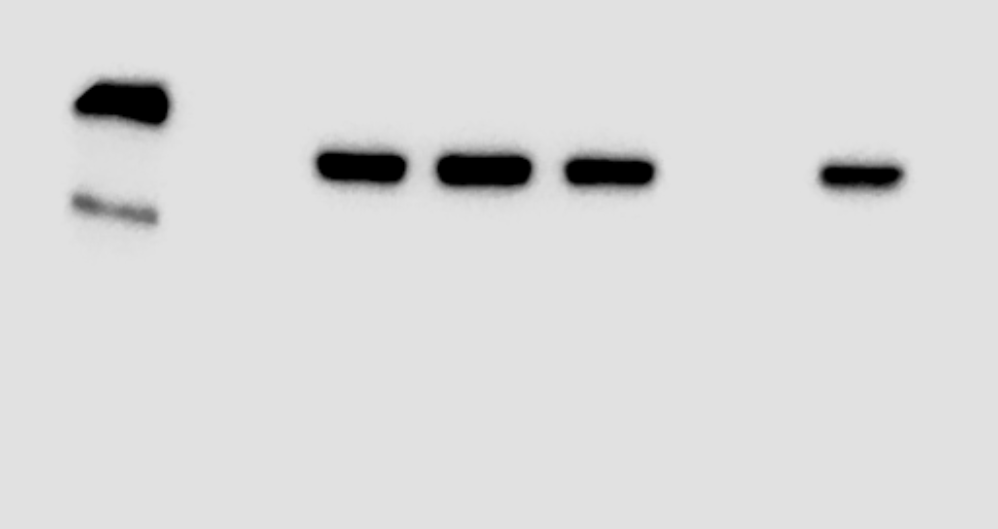

Supplement: Supplementary file 1 [file biomolecules-16-00856-s001.zip › Western Blot Files/Figure 1 (ER)/Fig1_MCF7TamR_Rep2_ERalpha_RAW.tif]

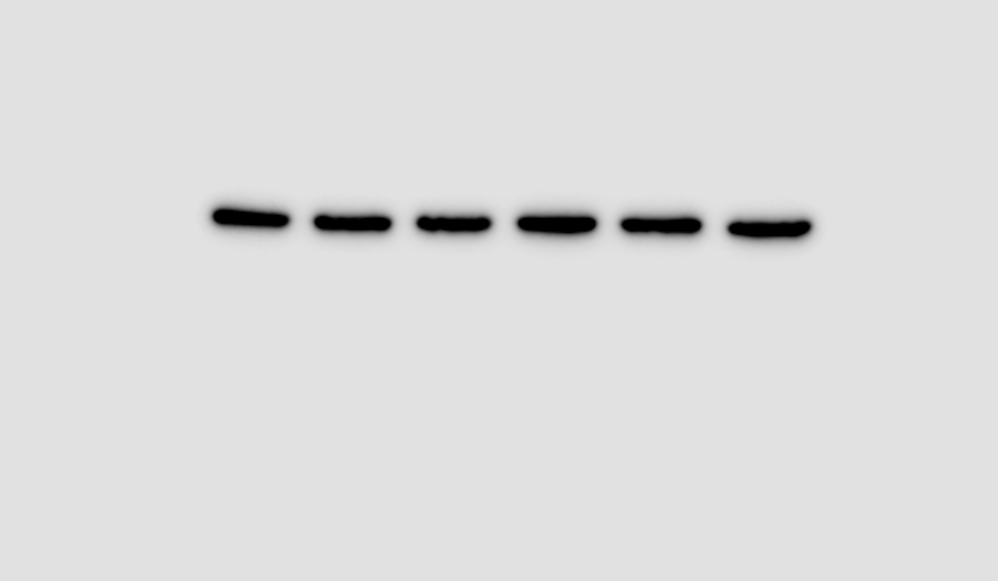

Supplement: Supplementary file 1 [file biomolecules-16-00856-s001.zip › Western Blot Files/Figure 1 (ER)/Fig1_MCF7TamR_Rep3_beta-actin_RAW.tif]

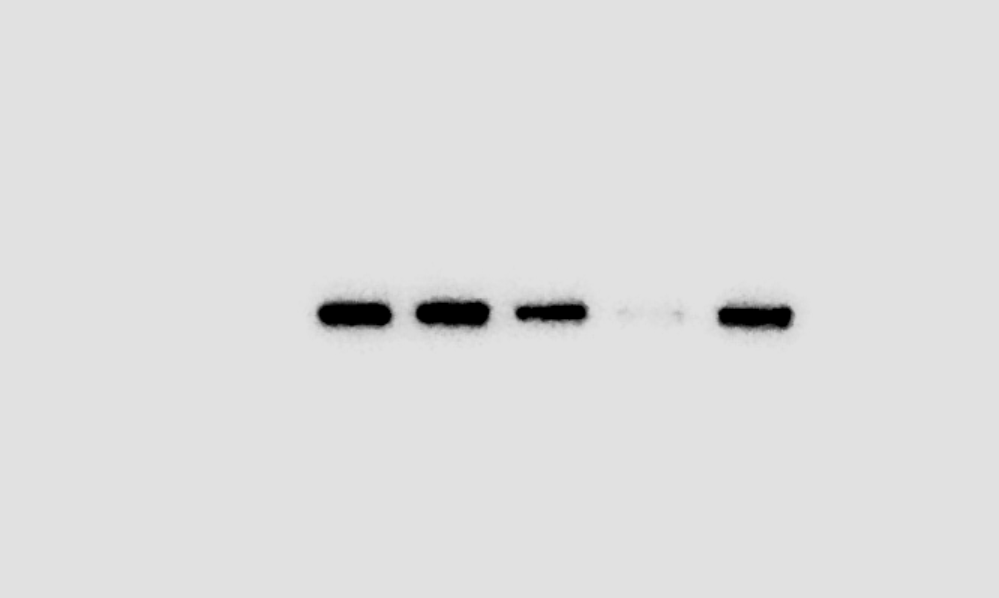

Supplement: Supplementary file 1 [file biomolecules-16-00856-s001.zip › Western Blot Files/Figure 1 (ER)/Fig1_MCF7TamR_Rep3_ERalpha_RAW.tif]

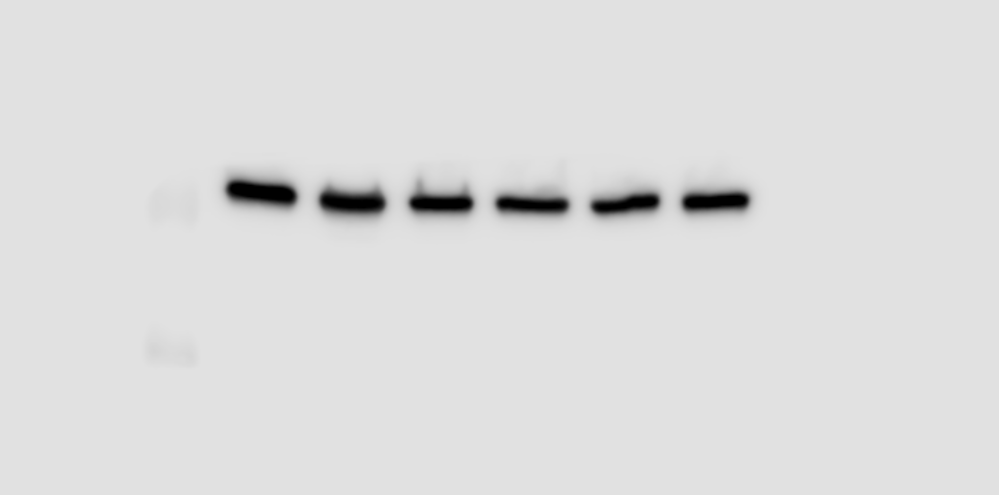

Supplement: Supplementary file 1 [file biomolecules-16-00856-s001.zip › Western Blot Files/Figure 1 (ER)/Fig1_MCF7_Rep1_beta-actin_RAW.tif]

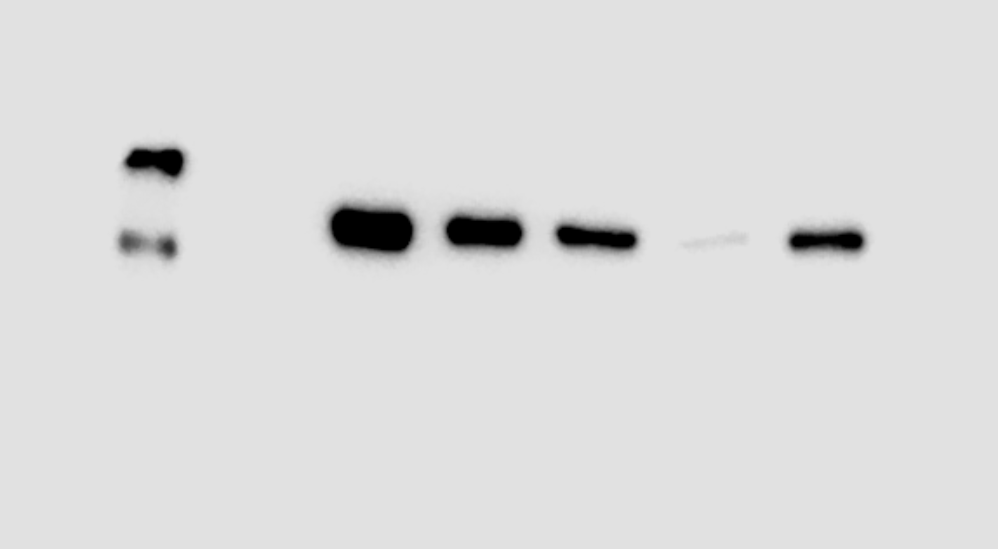

Supplement: Supplementary file 1 [file biomolecules-16-00856-s001.zip › Western Blot Files/Figure 1 (ER)/Fig1_MCF7_Rep1_ERalpha_RAW.tif]

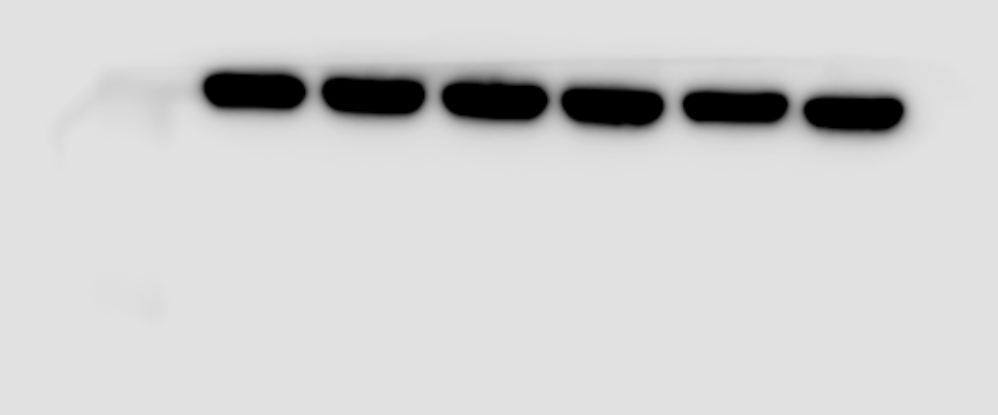

Supplement: Supplementary file 1 [file biomolecules-16-00856-s001.zip › Western Blot Files/Figure 1 (ER)/Fig1_MCF7_Rep2_beta-actin_RAW.tif]

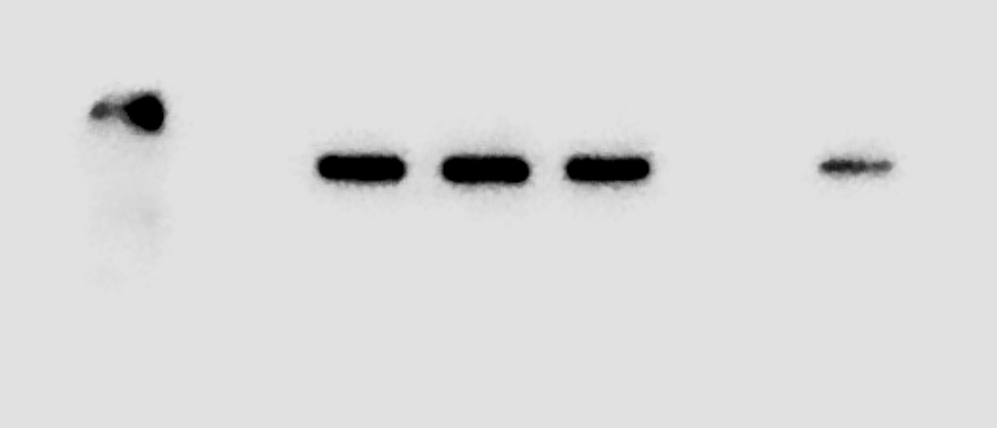

Supplement: Supplementary file 1 [file biomolecules-16-00856-s001.zip › Western Blot Files/Figure 1 (ER)/Fig1_MCF7_Rep2_ERalpha_RAW.tif]

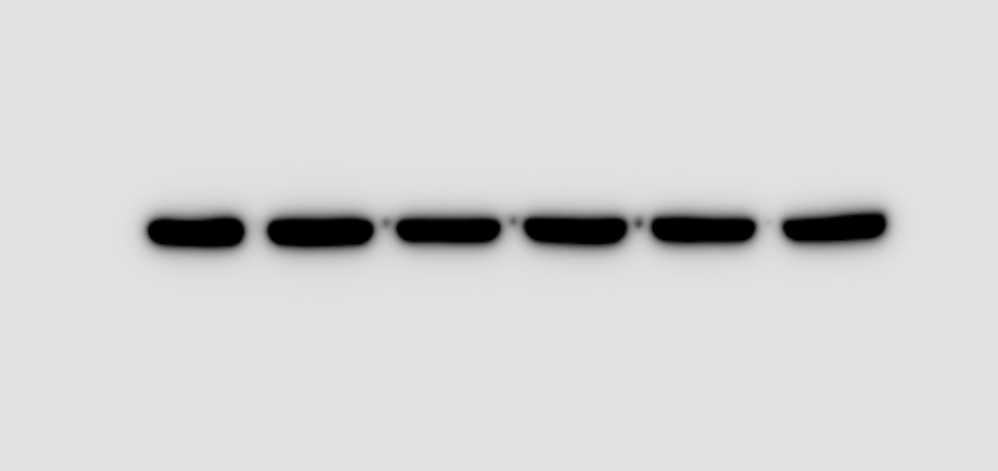

Supplement: Supplementary file 1 [file biomolecules-16-00856-s001.zip › Western Blot Files/Figure 1 (ER)/Fig1_MCF7_Rep3_beta-actin_RAW.tif]

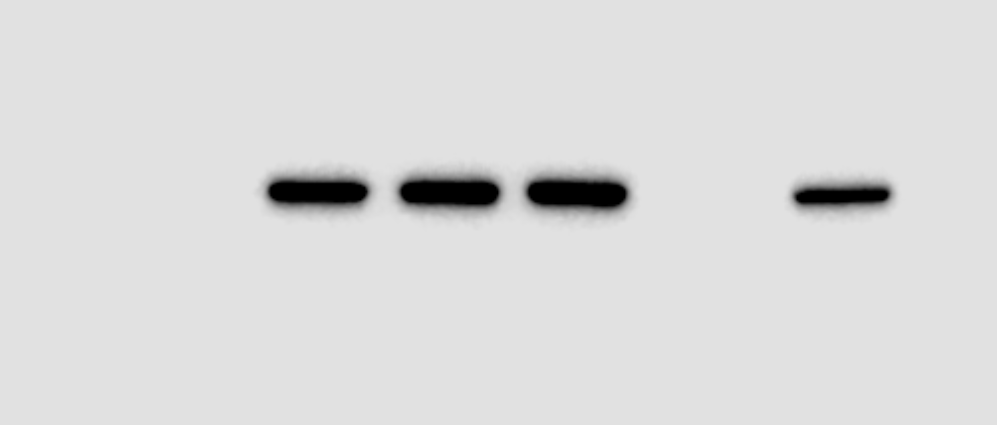

Supplement: Supplementary file 1 [file biomolecules-16-00856-s001.zip › Western Blot Files/Figure 1 (ER)/Fig1_MCF7_Rep3_ERalpha_RAW.tif]

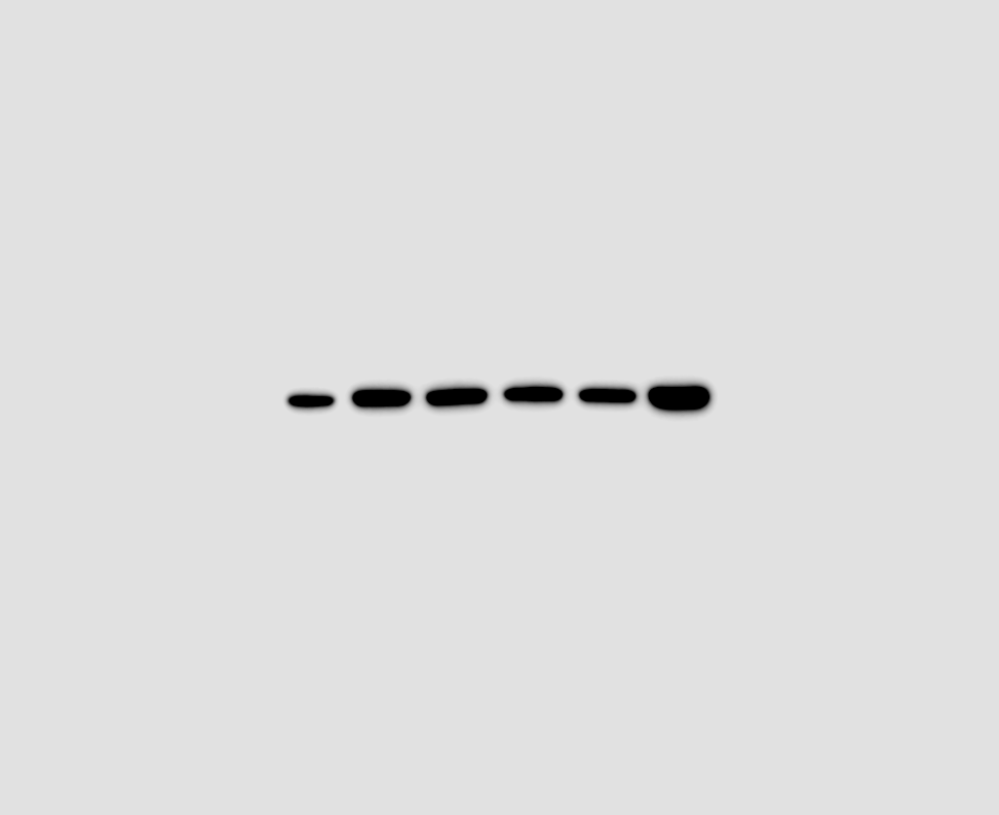

Supplement: Supplementary file 1 [file biomolecules-16-00856-s001.zip › Western Blot Files/Figure 1 (ER)/Fig1_T47D_Rep1_beta-actin_RAW.tif]

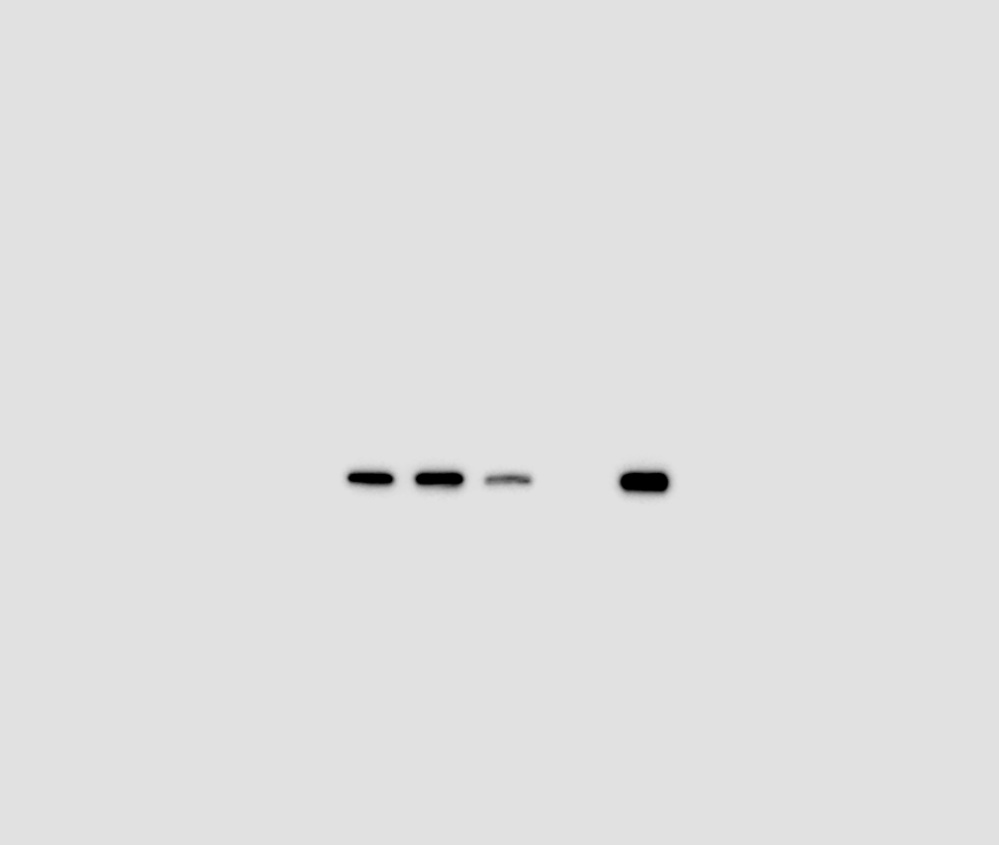

Supplement: Supplementary file 1 [file biomolecules-16-00856-s001.zip › Western Blot Files/Figure 1 (ER)/Fig1_T47D_Rep1_ERalpha_RAW.tif]

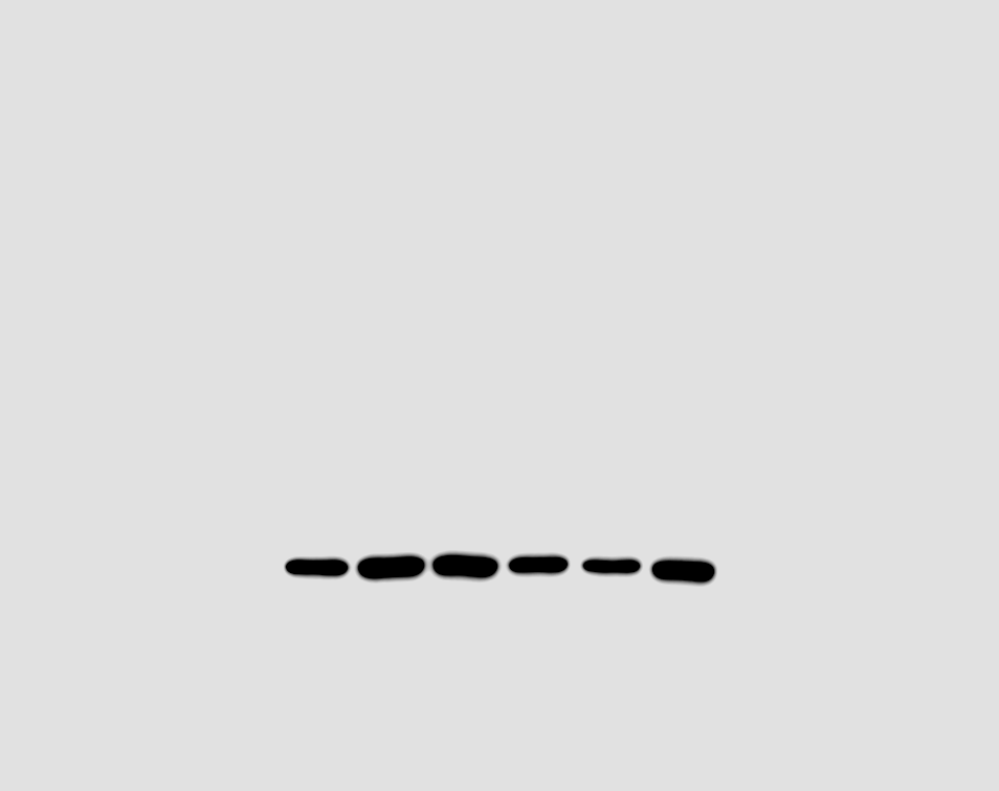

Supplement: Supplementary file 1 [file biomolecules-16-00856-s001.zip › Western Blot Files/Figure 1 (ER)/Fig1_T47D_Rep2_beta-actin_RAW.tif]

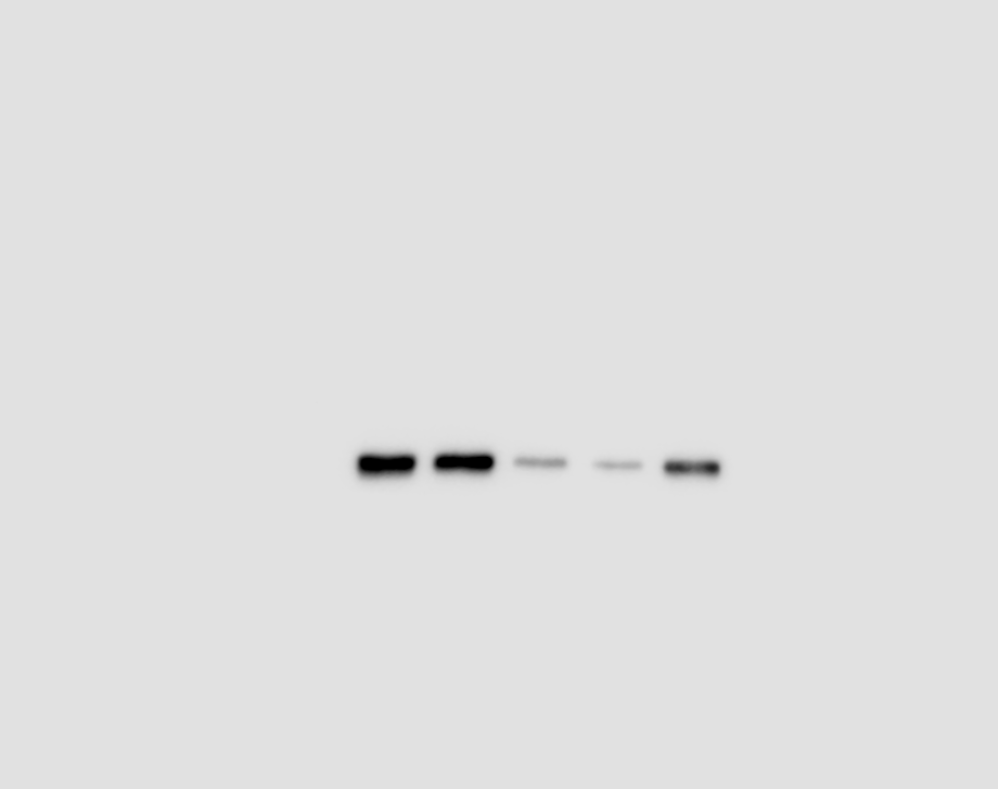

Supplement: Supplementary file 1 [file biomolecules-16-00856-s001.zip › Western Blot Files/Figure 1 (ER)/Fig1_T47D_Rep2_ERalpha_RAW.tif]

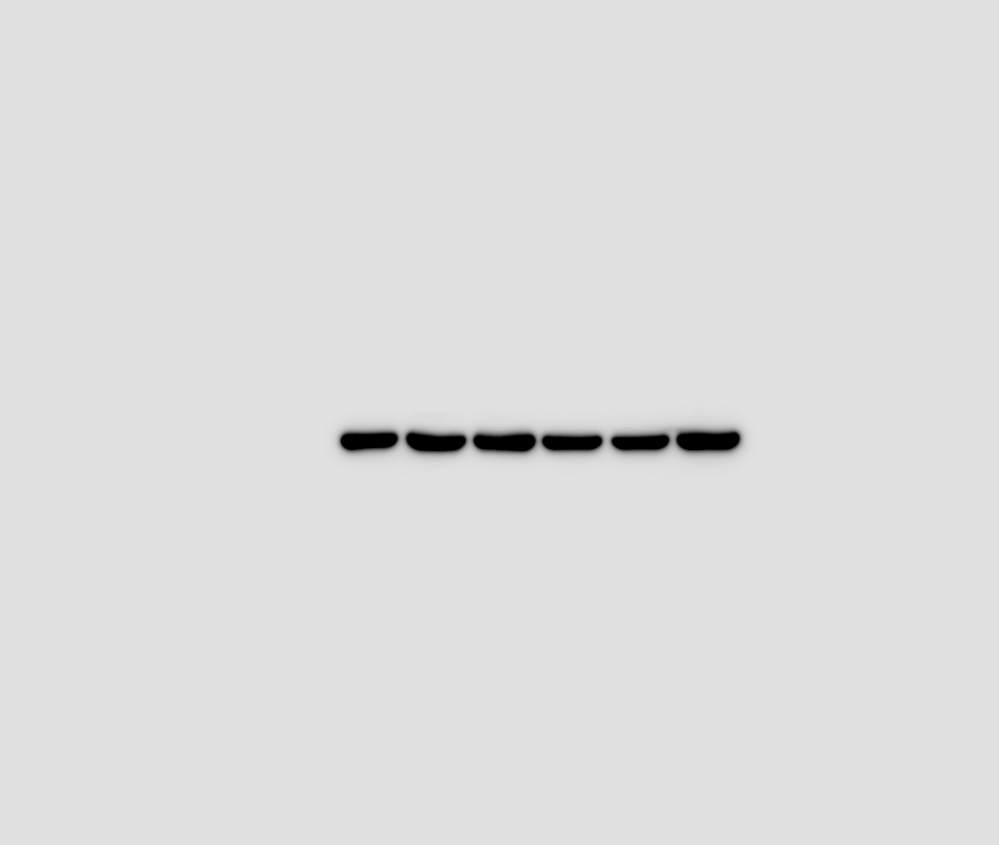

Supplement: Supplementary file 1 [file biomolecules-16-00856-s001.zip › Western Blot Files/Figure 1 (ER)/Fig1_T47D_Rep3_beta-actin_RAW.tif]

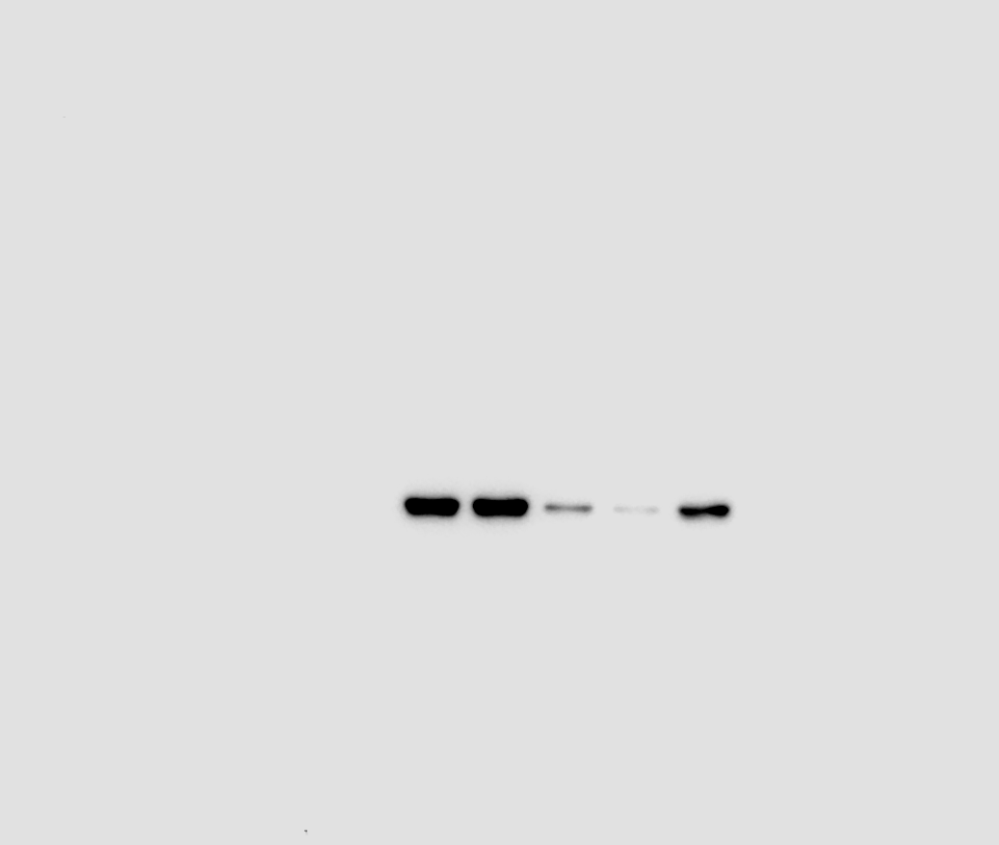

Supplement: Supplementary file 1 [file biomolecules-16-00856-s001.zip › Western Blot Files/Figure 1 (ER)/Fig1_T47D_Rep3_ERalpha_RAW.tif]

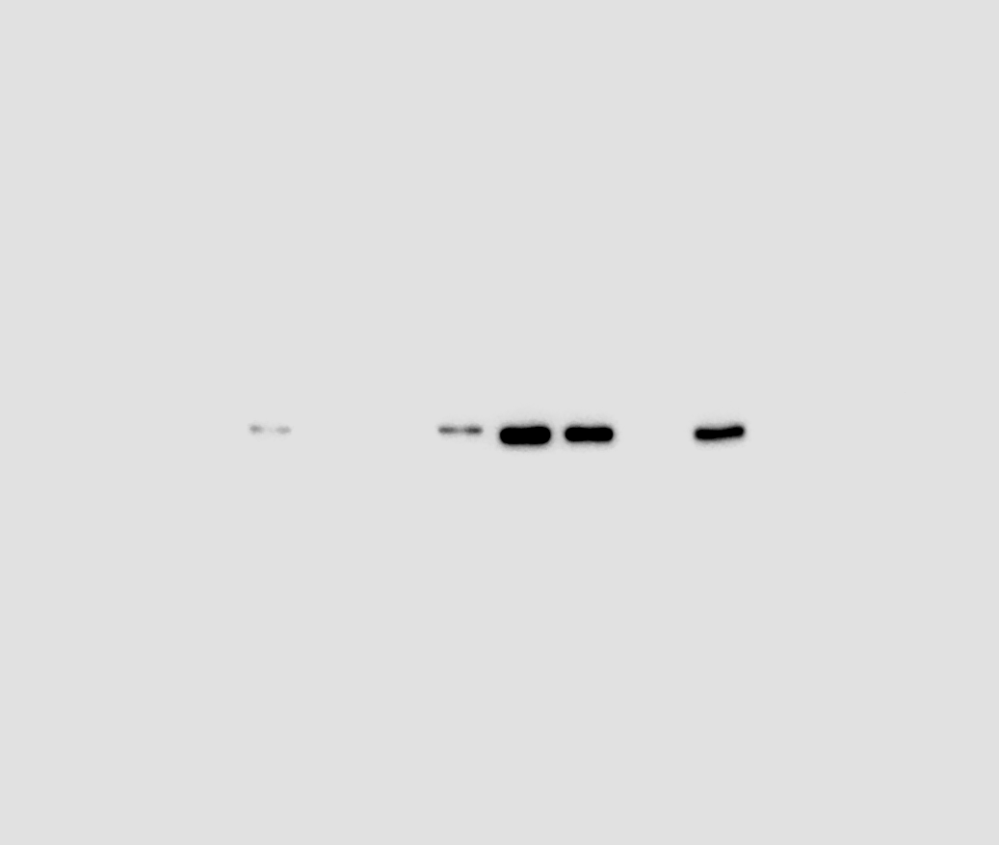

Supplement: Supplementary file 1 [file biomolecules-16-00856-s001.zip › Western Blot Files/Figure 2 (AR)/Fig2_MCF7vsMCF7TamR_Rep1_AR_RAW.tif]

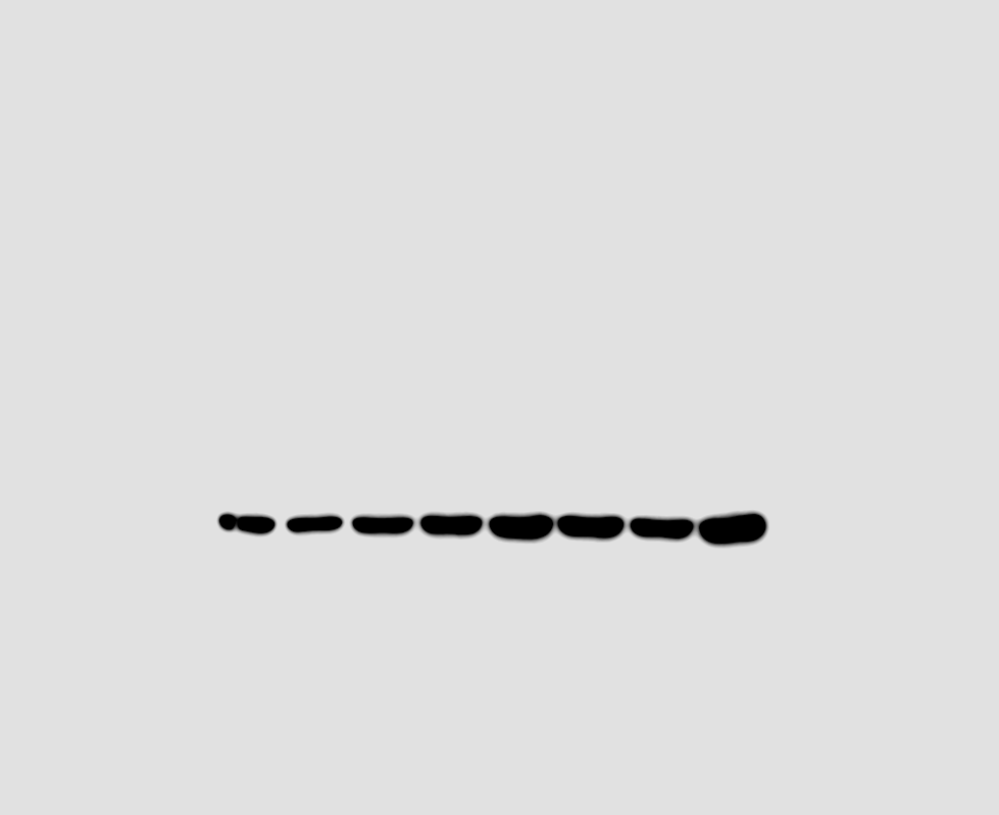

Supplement: Supplementary file 1 [file biomolecules-16-00856-s001.zip › Western Blot Files/Figure 2 (AR)/Fig2_MCF7vsMCF7TamR_Rep1_beta-actin_RAW.tif]

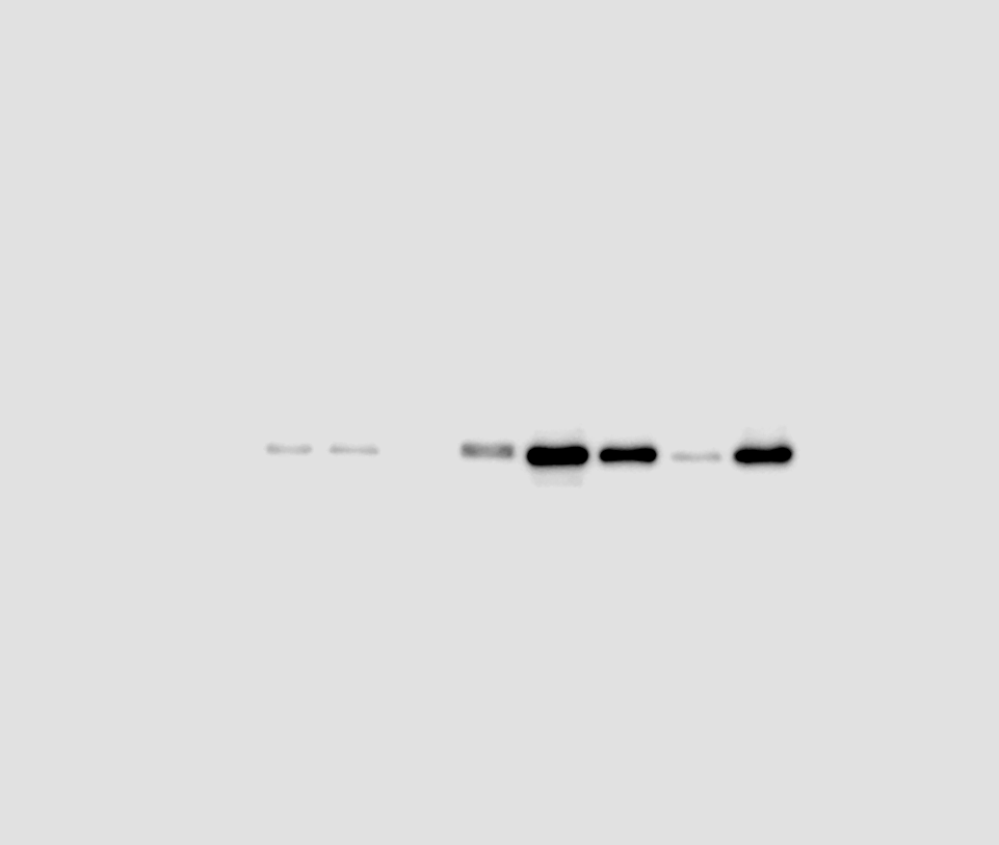

Supplement: Supplementary file 1 [file biomolecules-16-00856-s001.zip › Western Blot Files/Figure 2 (AR)/Fig2_MCF7vsMCF7TamR_Rep2_AR_RAW.tif]

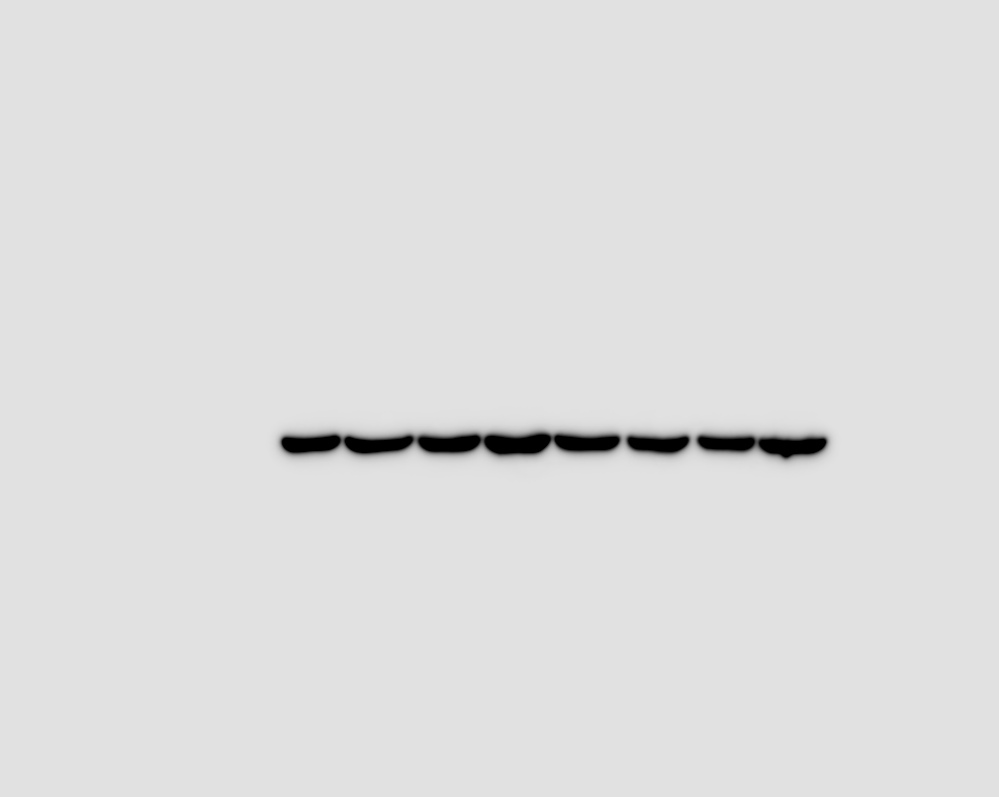

Supplement: Supplementary file 1 [file biomolecules-16-00856-s001.zip › Western Blot Files/Figure 2 (AR)/Fig2_MCF7vsMCF7TamR_Rep2_beta-actin_RAW.tif]

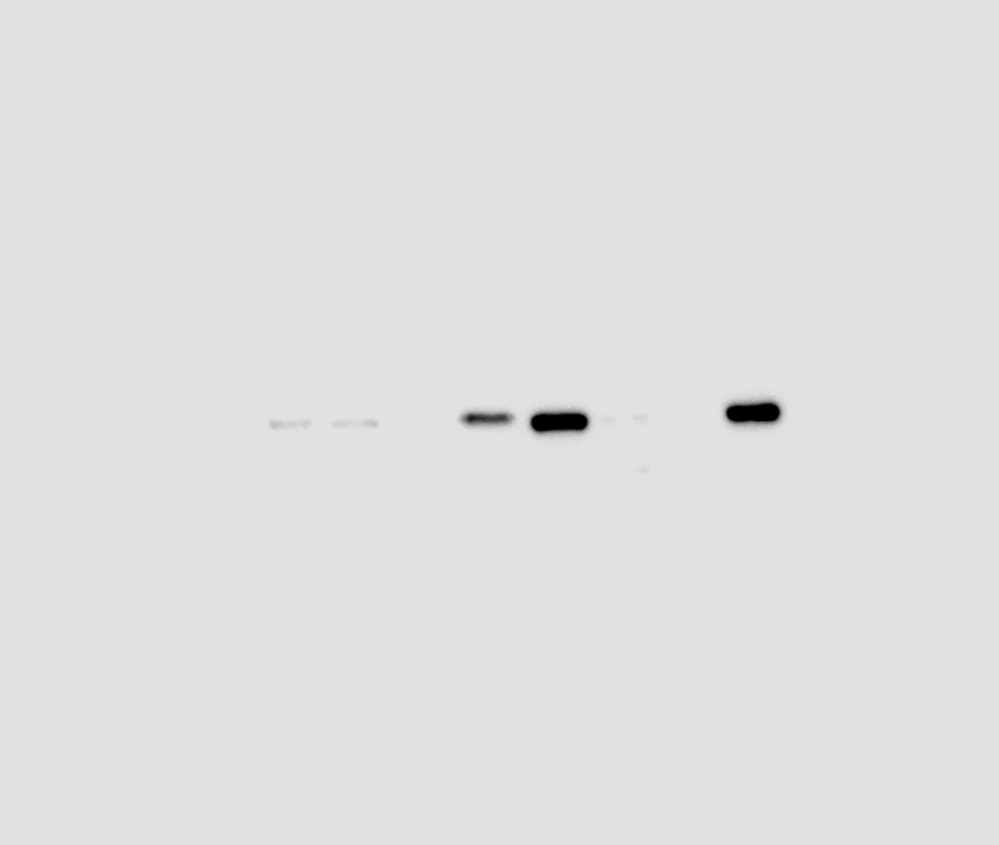

Supplement: Supplementary file 1 [file biomolecules-16-00856-s001.zip › Western Blot Files/Figure 2 (AR)/Fig2_MCF7vsMCF7TamR_Rep3_AR_RAW.tif]

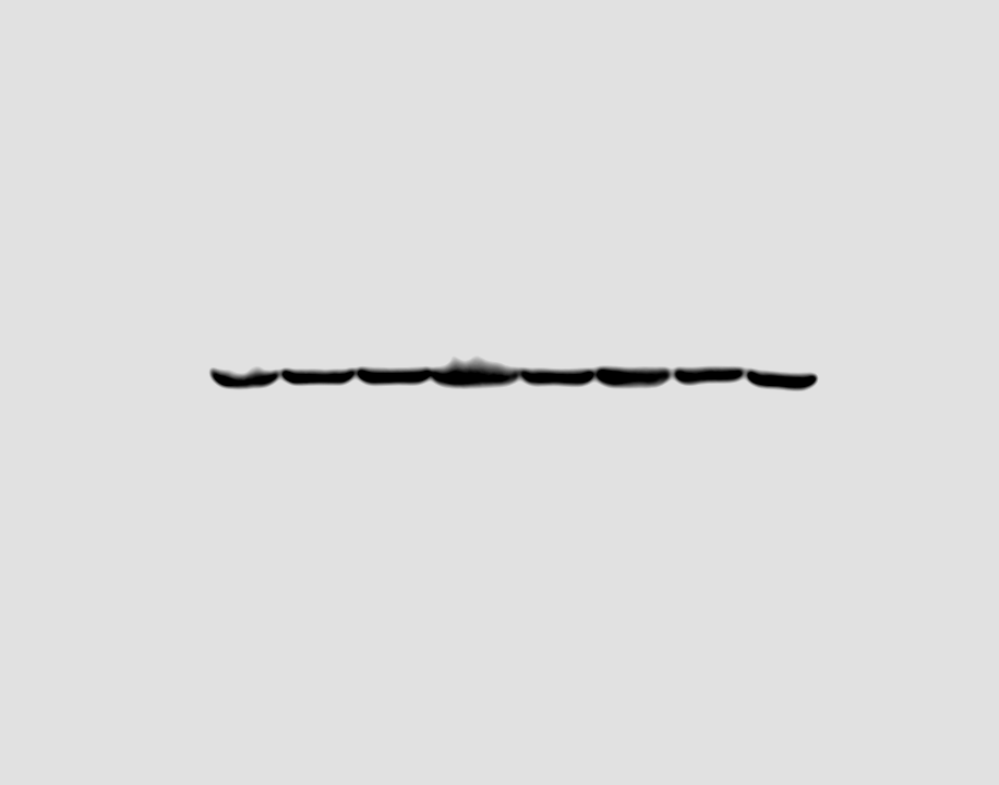

Supplement: Supplementary file 1 [file biomolecules-16-00856-s001.zip › Western Blot Files/Figure 2 (AR)/Fig2_MCF7vsMCF7TamR_Rep3_beta-actin_RAW.tif]

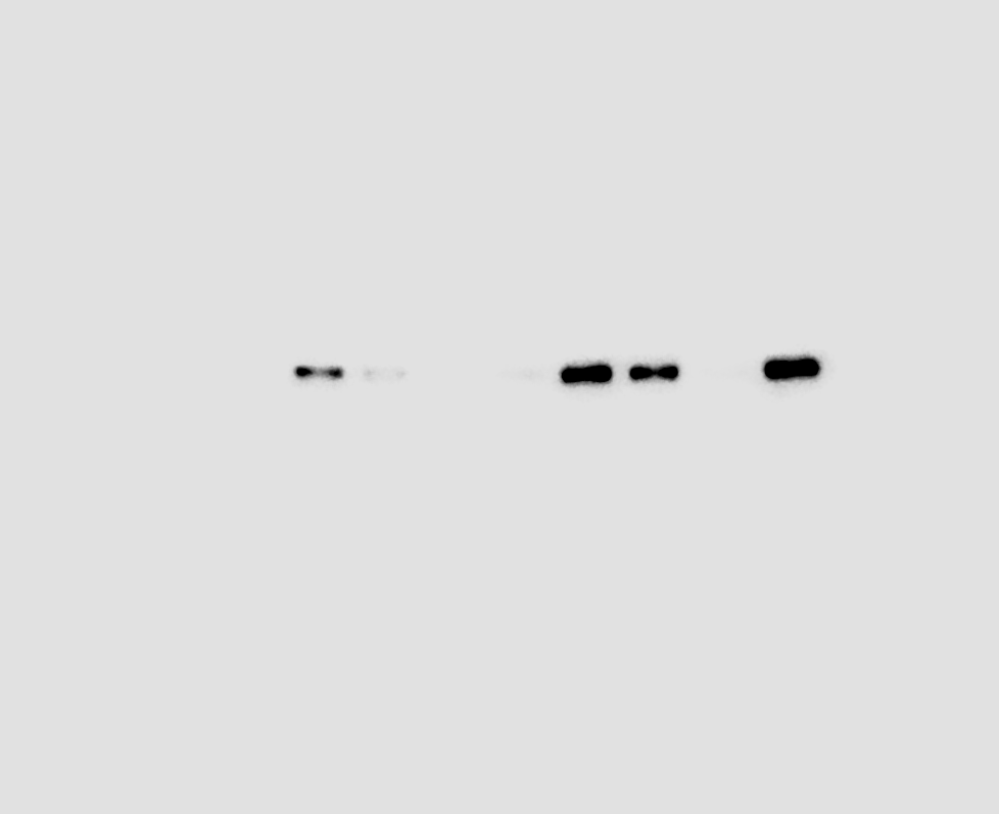

Supplement: Supplementary file 1 [file biomolecules-16-00856-s001.zip › Western Blot Files/Figure 3 (HER2)/Fig3_MCF7vsMCF7TamR_Rep1_HER2_RAW.tif]

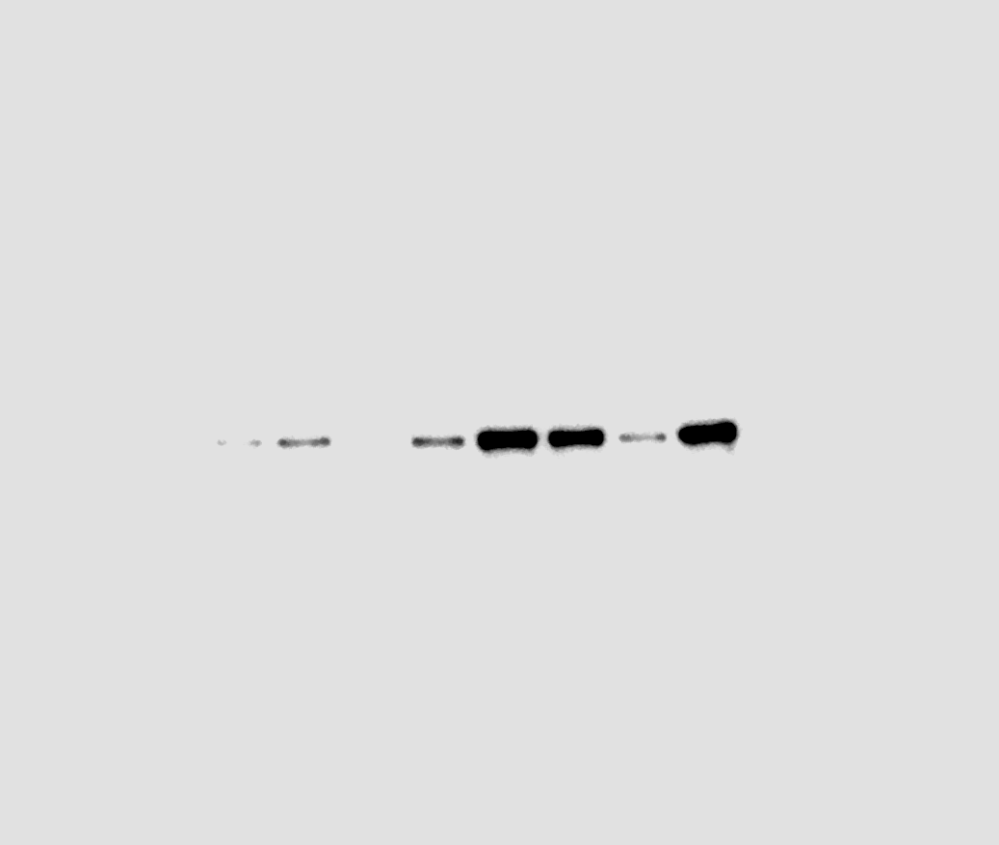

Supplement: Supplementary file 1 [file biomolecules-16-00856-s001.zip › Western Blot Files/Figure 3 (HER2)/Fig3_MCF7vsMCF7TamR_Rep2_HER2_RAW.tif.tif]

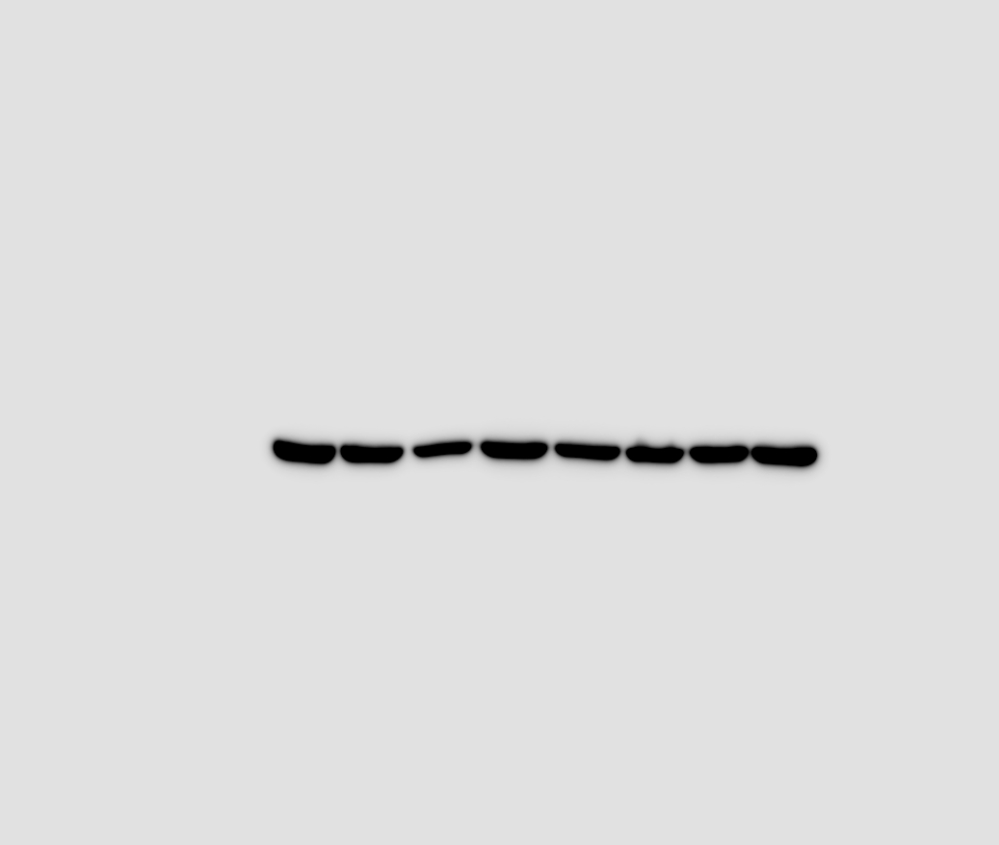

Supplement: Supplementary file 1 [file biomolecules-16-00856-s001.zip › Western Blot Files/Figure 3 (HER2)/Fig3_MCF7vsMCF7TamR_Rep3_beta-actin_RAW.tif]

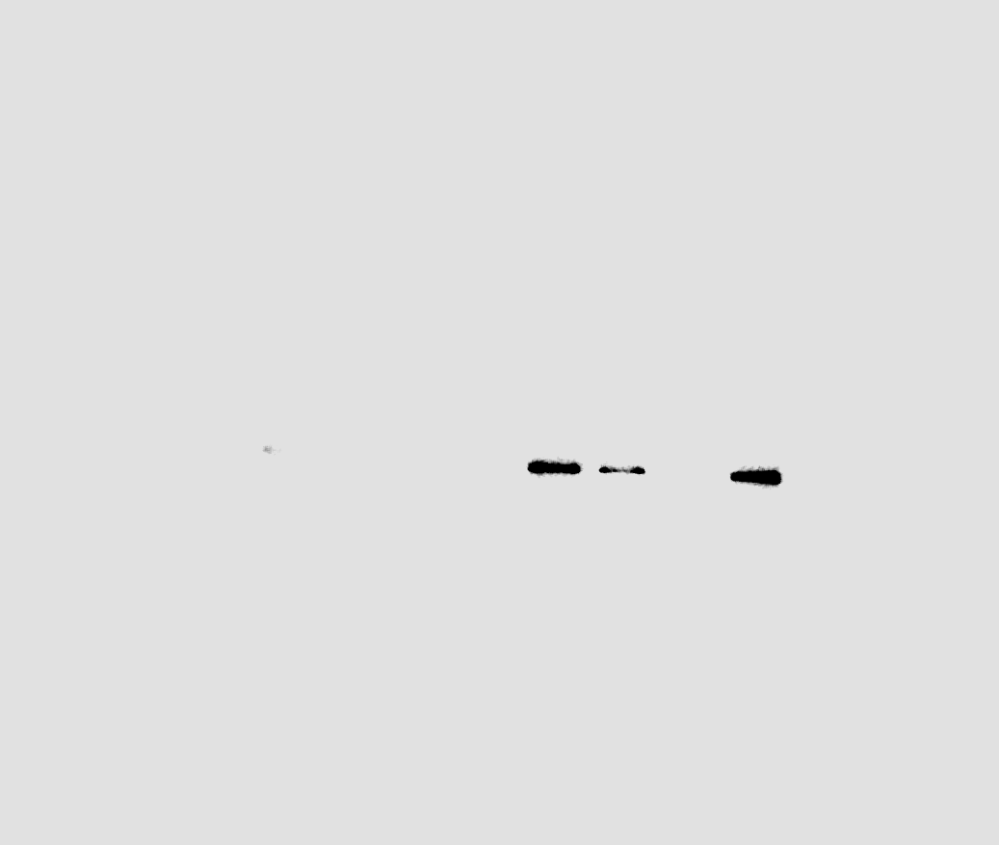

Supplement: Supplementary file 1 [file biomolecules-16-00856-s001.zip › Western Blot Files/Figure 3 (HER2)/Fig3_MCF7vsMCF7TamR_Rep3_HER2_RAW.tif]

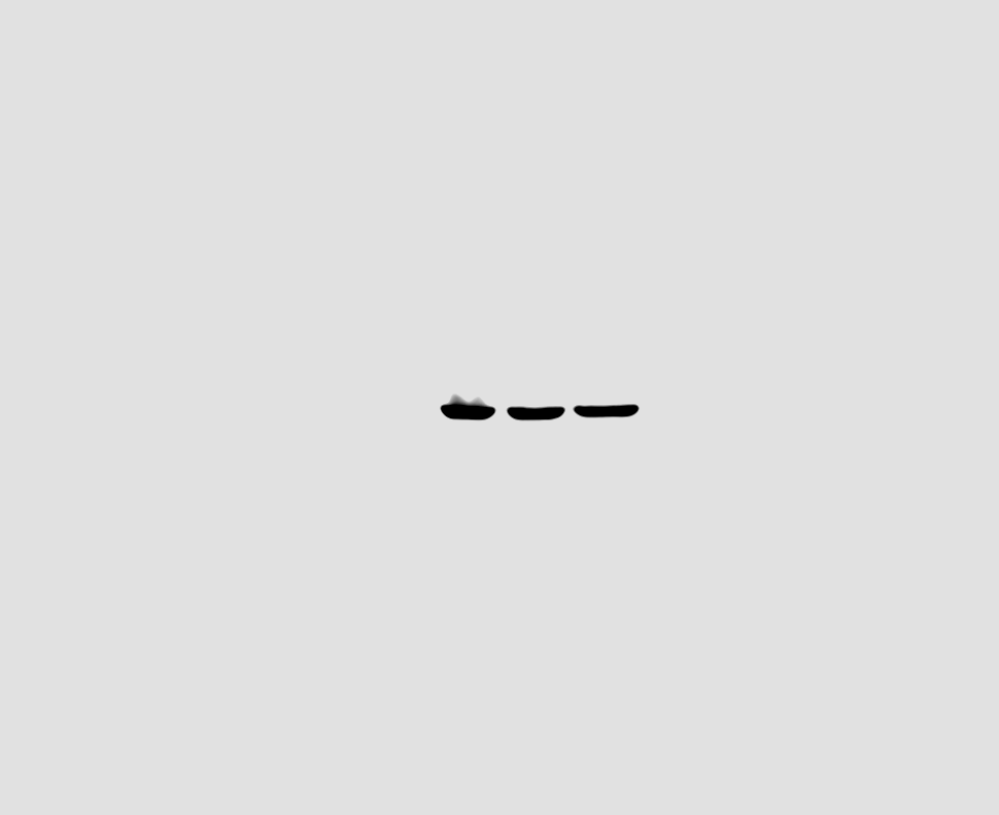

Supplement: Supplementary file 1 [file biomolecules-16-00856-s001.zip › Western Blot Files/Figure 4 (PARP)/Fig4_MCF7TamR_Rep1_beta-actin_RAW.tif]

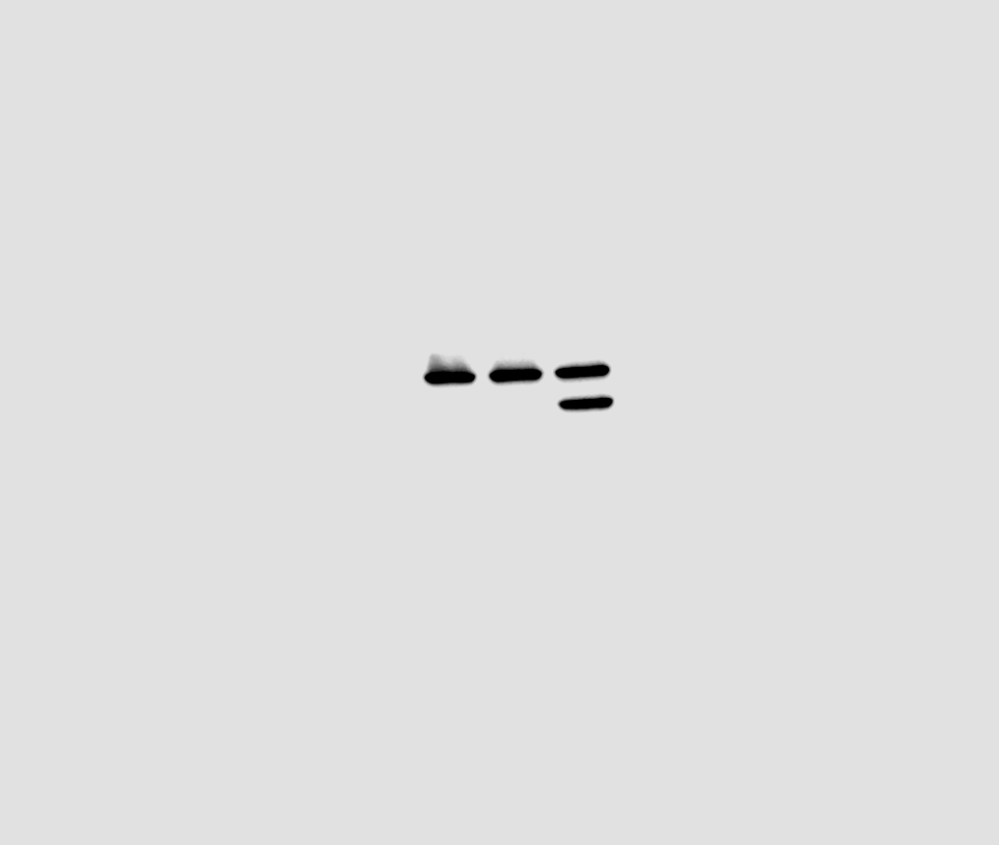

Supplement: Supplementary file 1 [file biomolecules-16-00856-s001.zip › Western Blot Files/Figure 4 (PARP)/Fig4_MCF7TamR_Rep1_PARP1_RAW.tif]

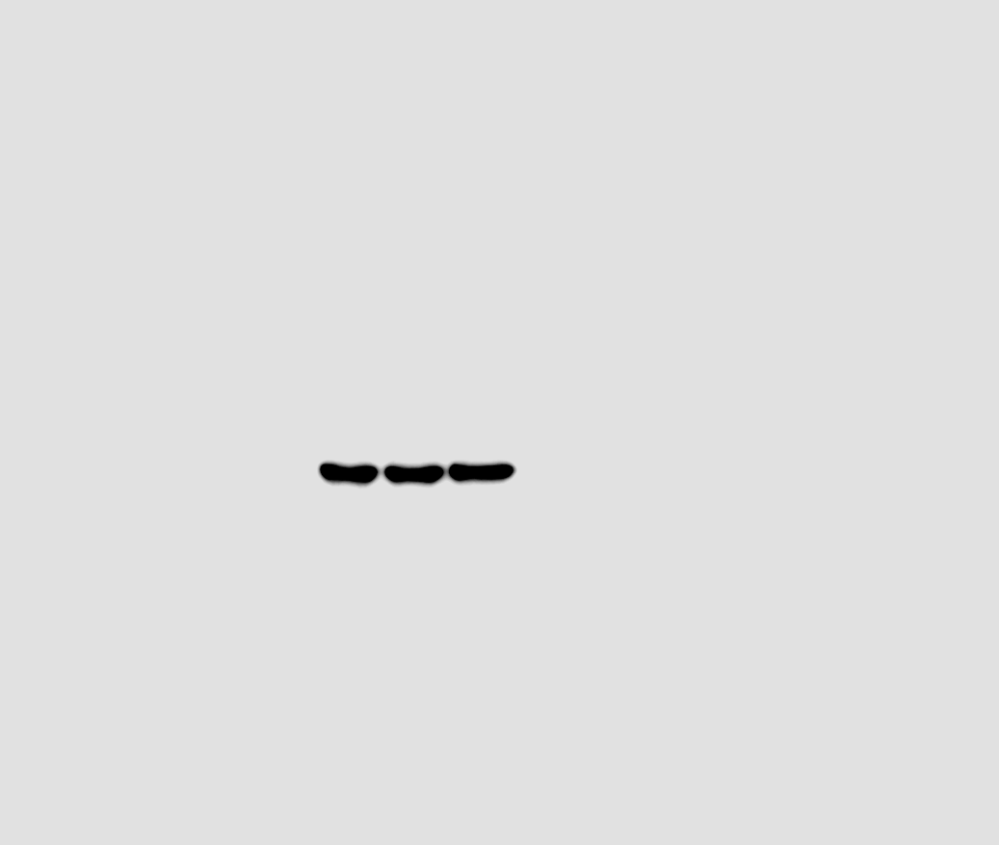

Supplement: Supplementary file 1 [file biomolecules-16-00856-s001.zip › Western Blot Files/Figure 4 (PARP)/Fig4_MCF7TamR_Rep2_beta-actin_RAW.tif]

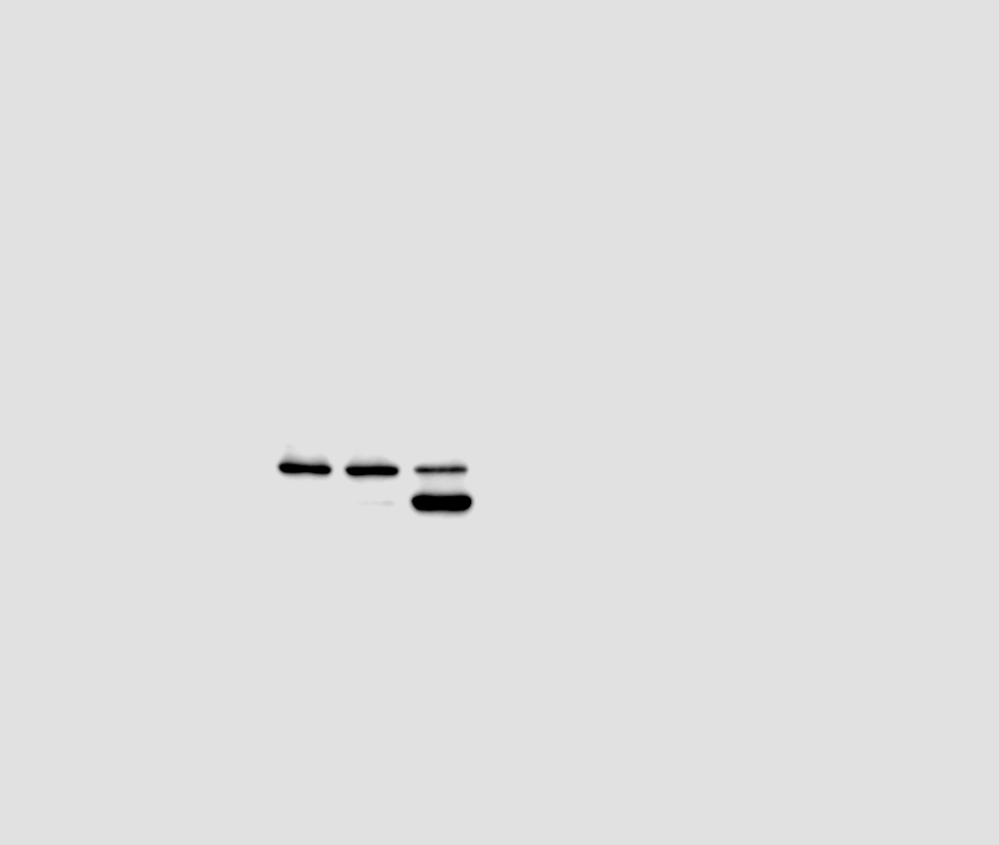

Supplement: Supplementary file 1 [file biomolecules-16-00856-s001.zip › Western Blot Files/Figure 4 (PARP)/Fig4_MCF7TamR_Rep2_PARP1_RAW.tif]

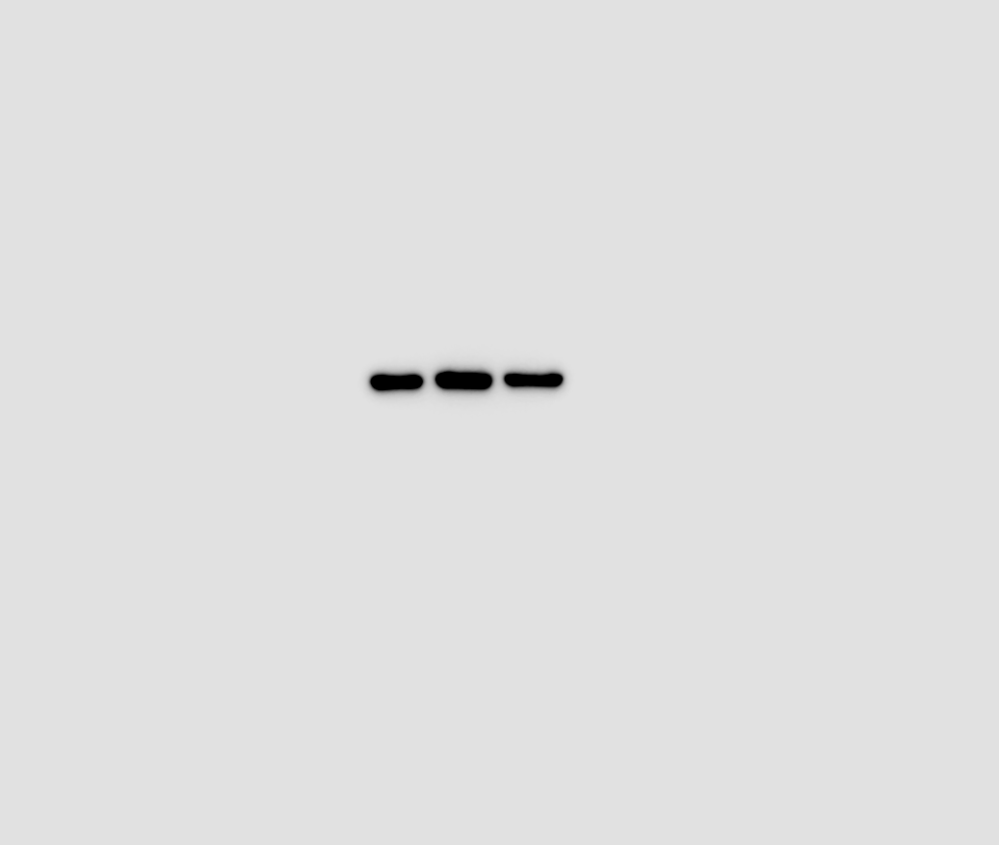

Supplement: Supplementary file 1 [file biomolecules-16-00856-s001.zip › Western Blot Files/Figure 4 (PARP)/Fig4_MCF7TamR_Rep3_beta-actin_RAW.tif]

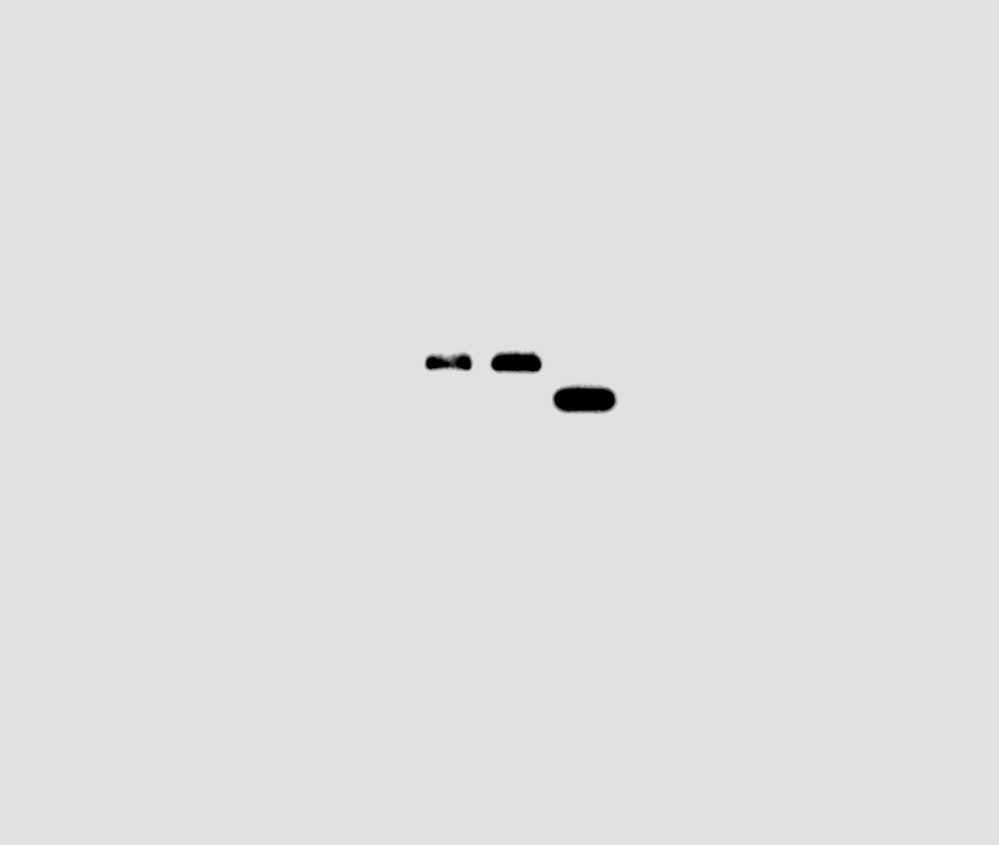

Supplement: Supplementary file 1 [file biomolecules-16-00856-s001.zip › Western Blot Files/Figure 4 (PARP)/Fig4_MCF7TamR_Rep3_PARP1_RAW.tif]

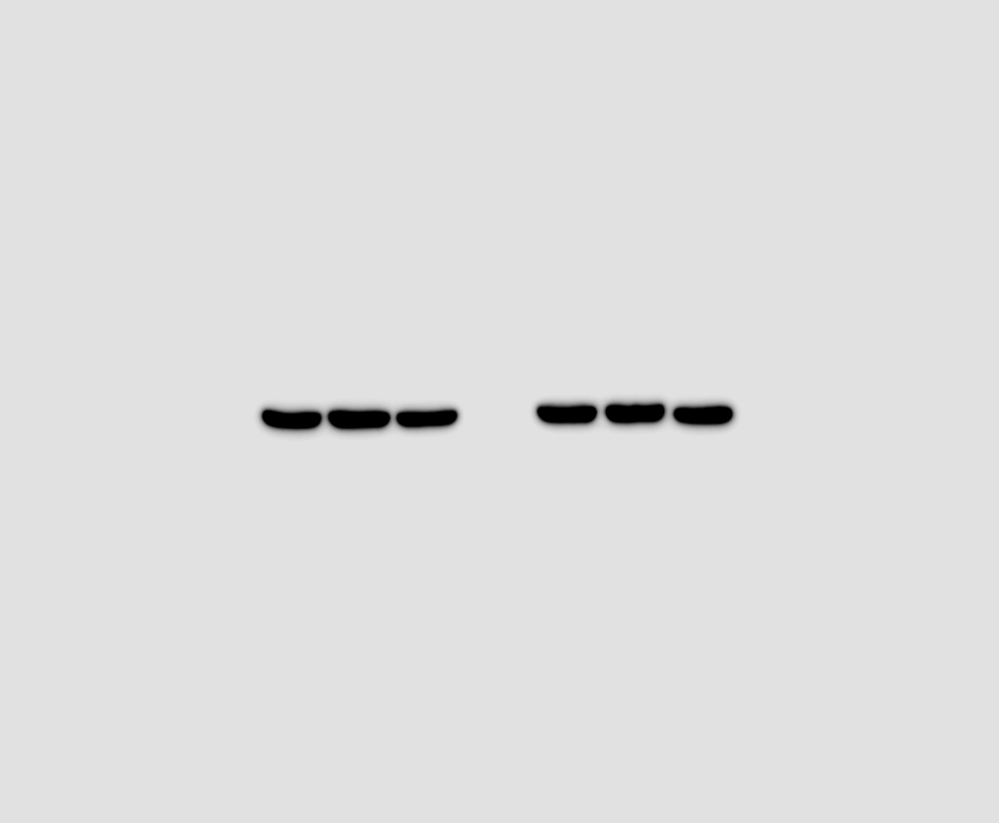

Supplement: Supplementary file 1 [file biomolecules-16-00856-s001.zip › Western Blot Files/Figure 4 (PARP)/Fig4_MCF7_Rep1_2_beta-actin_RAW.tif]

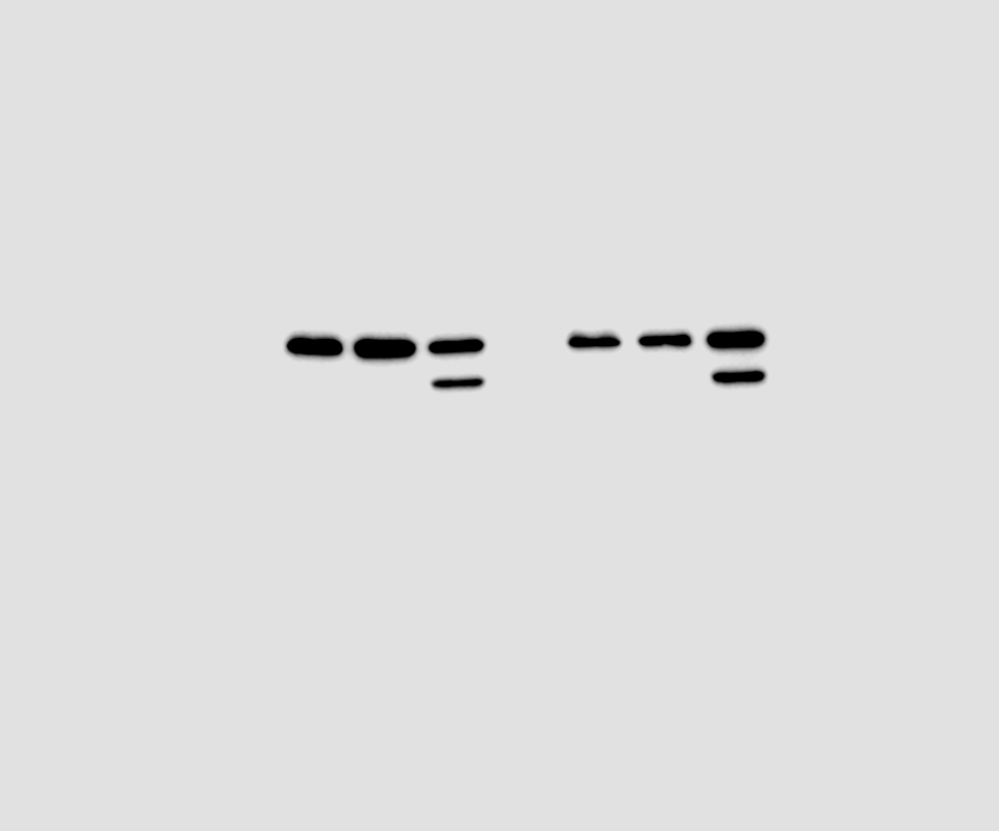

Supplement: Supplementary file 1 [file biomolecules-16-00856-s001.zip › Western Blot Files/Figure 4 (PARP)/Fig4_MCF7_Rep1_2_PARP1_RAW.tif]

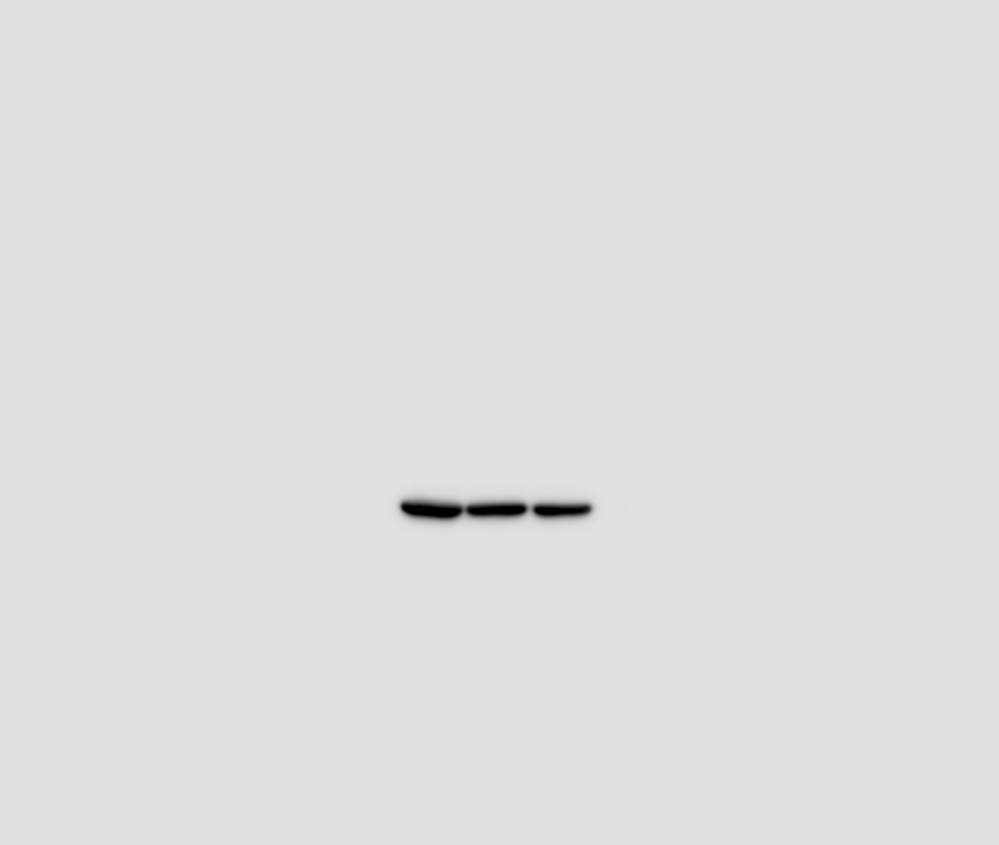

Supplement: Supplementary file 1 [file biomolecules-16-00856-s001.zip › Western Blot Files/Figure 4 (PARP)/Fig4_MCF7_Rep3_beta-actin_RAW.tif]

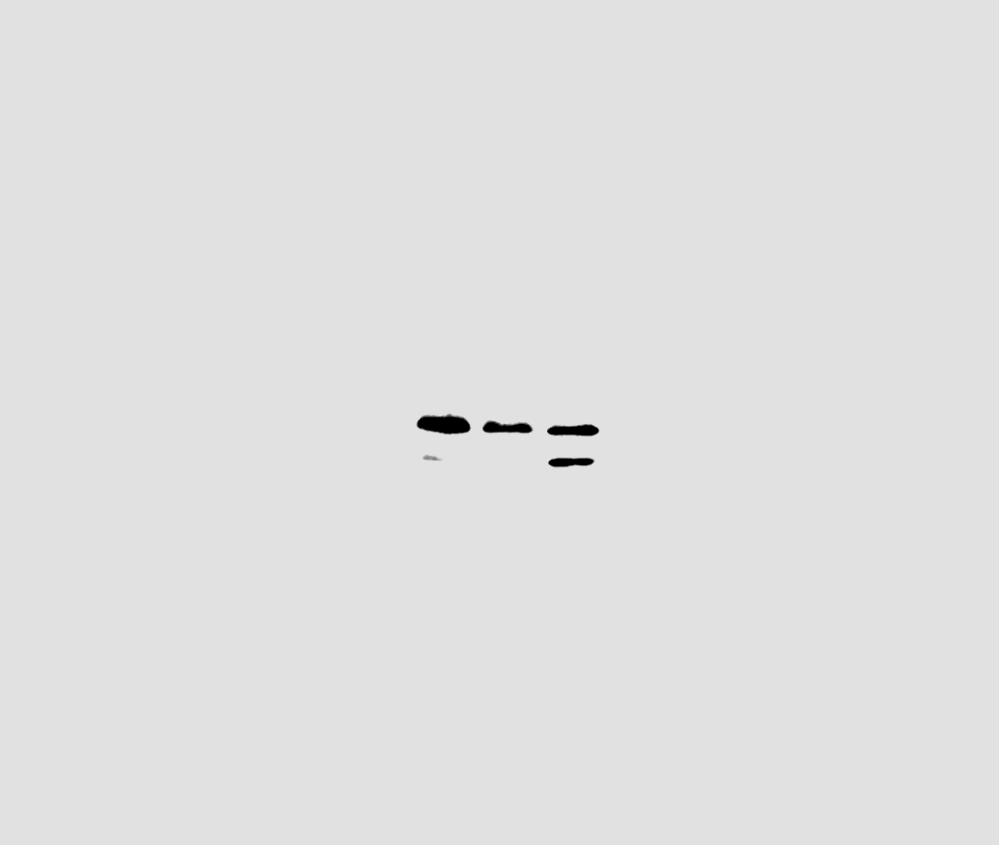

Supplement: Supplementary file 1 [file biomolecules-16-00856-s001.zip › Western Blot Files/Figure 4 (PARP)/Fig4_MCF7_Rep3_PARP1_RAW.tif]

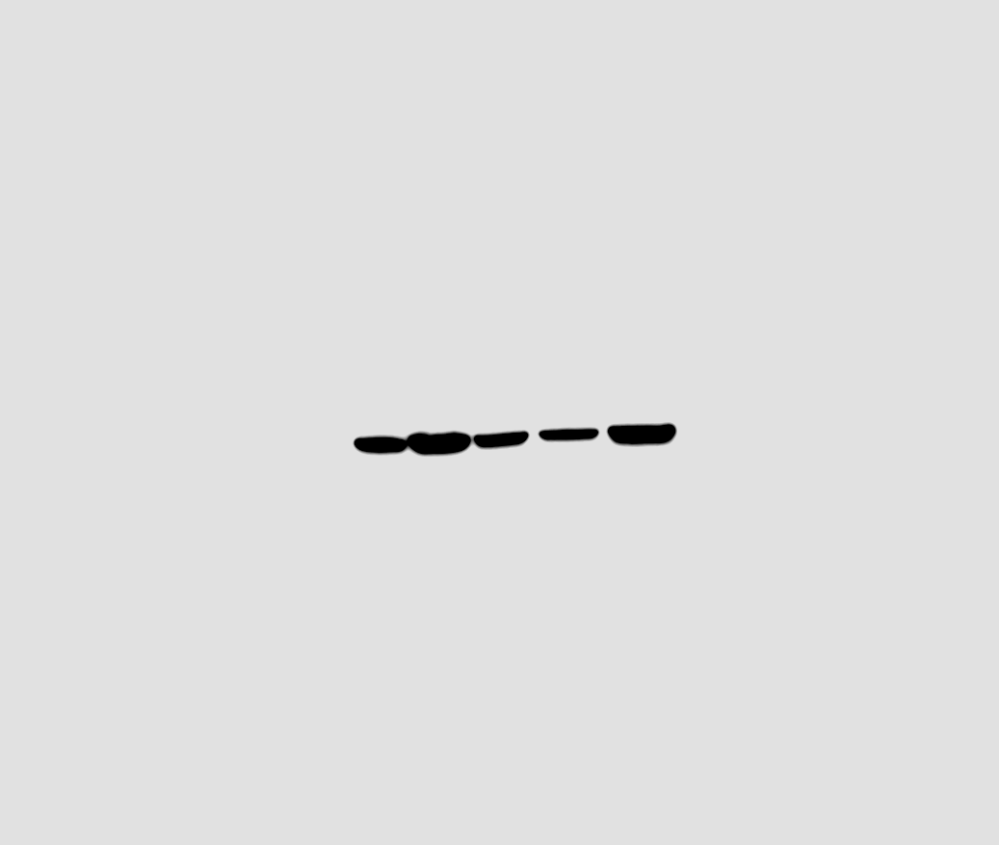

Supplement: Supplementary file 1 [file biomolecules-16-00856-s001.zip › Western Blot Files/Figure 6 (LXR)/Fig6_MCF7TamR_Rep1_beta-actin_RAW.tif]

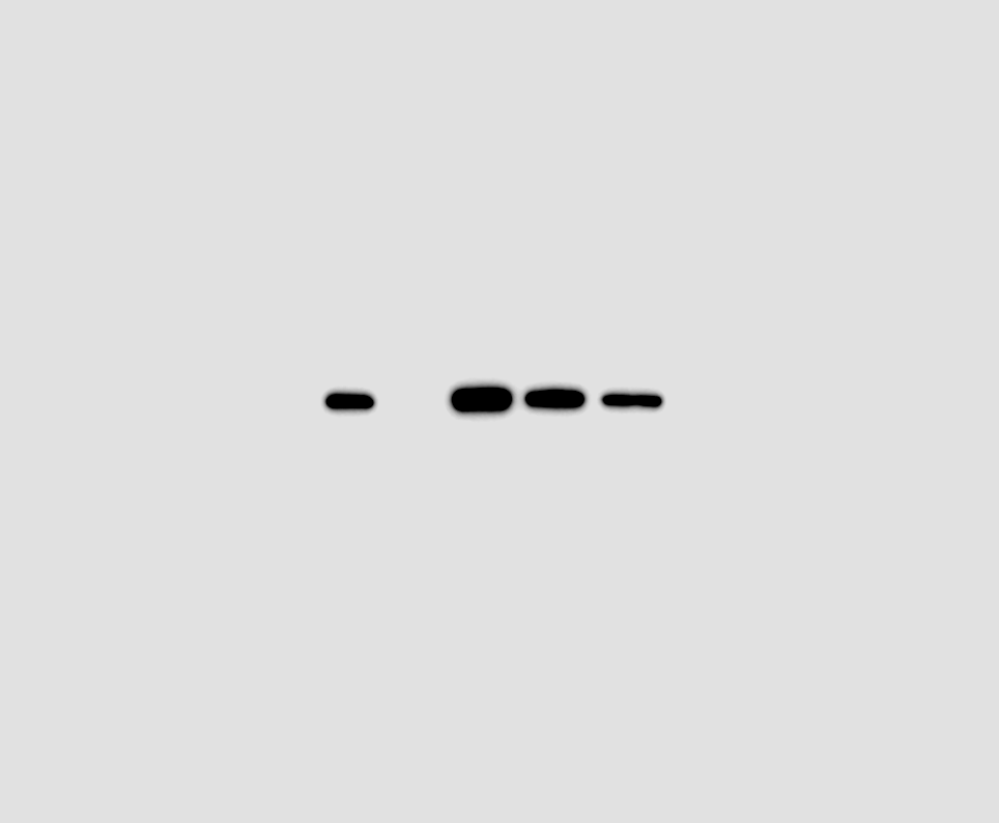

Supplement: Supplementary file 1 [file biomolecules-16-00856-s001.zip › Western Blot Files/Figure 6 (LXR)/Fig6_MCF7TamR_Rep1_LXRb_RAW_restored.tif]

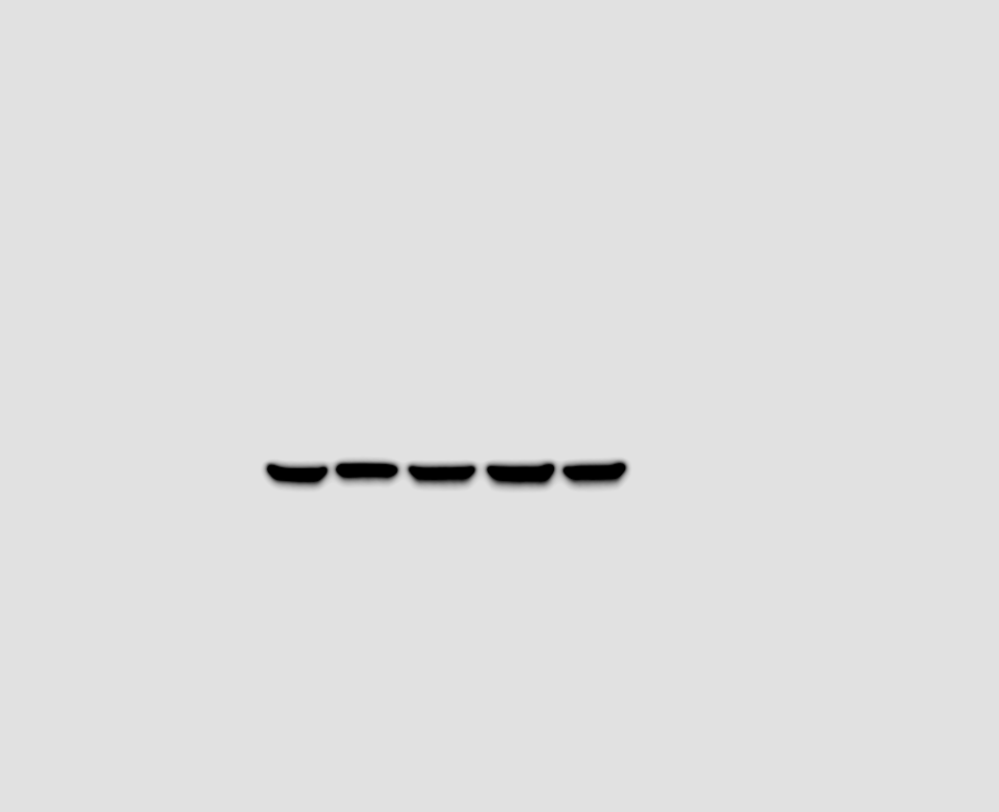

Supplement: Supplementary file 1 [file biomolecules-16-00856-s001.zip › Western Blot Files/Figure 6 (LXR)/Fig6_MCF7TamR_Rep2_beta-actin_RAW.tif]

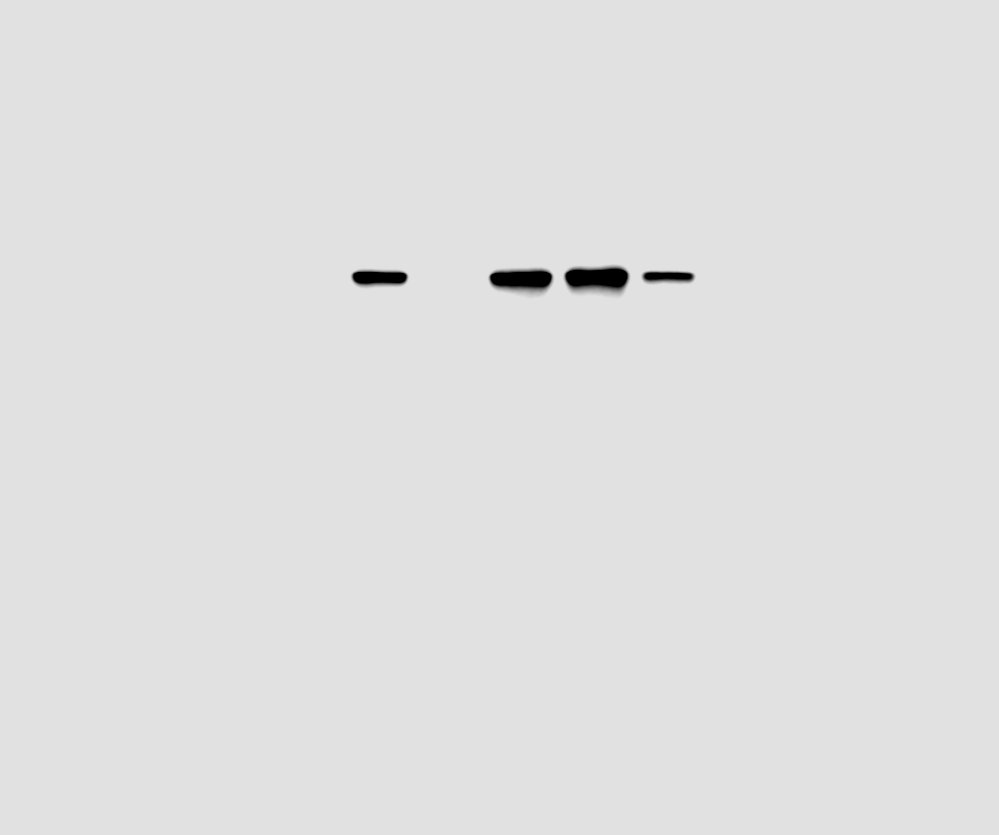

Supplement: Supplementary file 1 [file biomolecules-16-00856-s001.zip › Western Blot Files/Figure 6 (LXR)/Fig6_MCF7TamR_Rep2_LXRb_RAW_restored.tif]

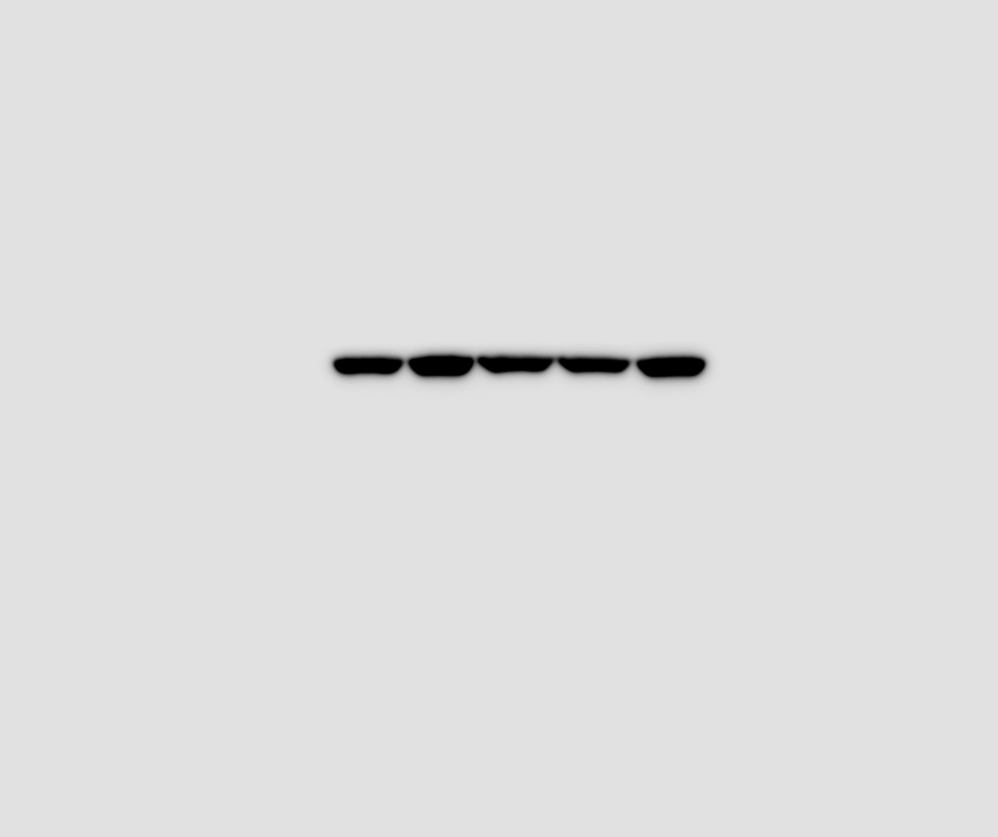

Supplement: Supplementary file 1 [file biomolecules-16-00856-s001.zip › Western Blot Files/Figure 6 (LXR)/Fig6_MCF7TamR_Rep3_beta-actin_RAW.tif]

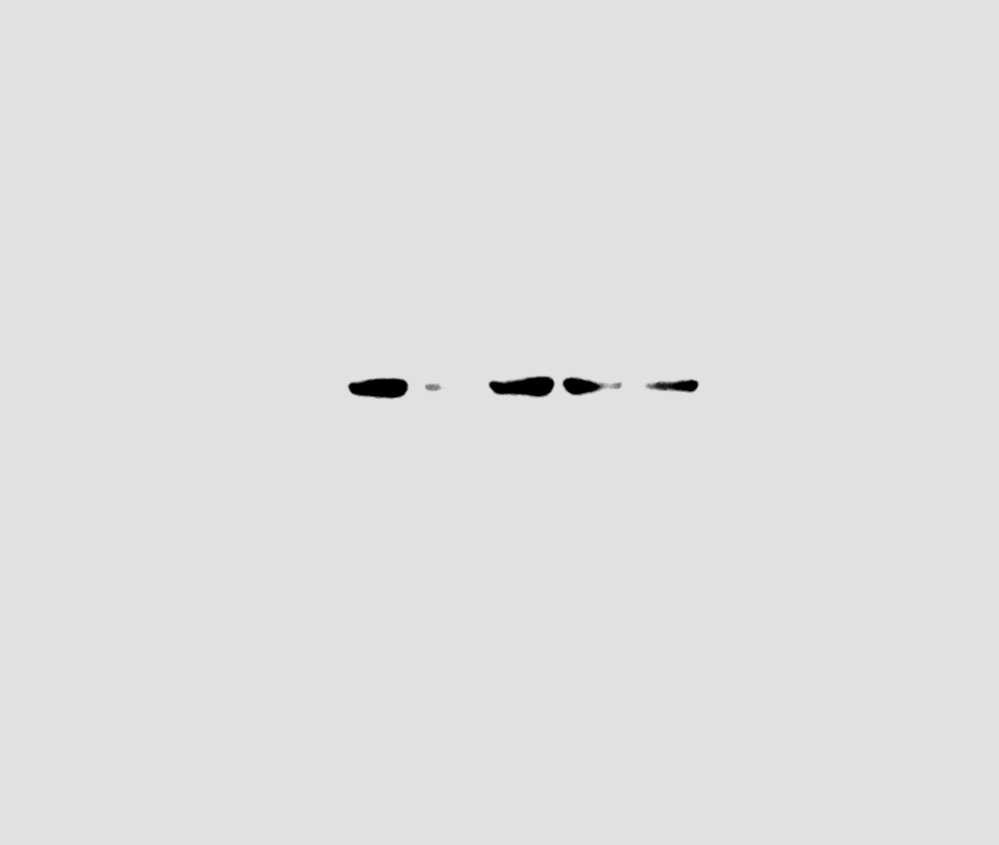

Supplement: Supplementary file 1 [file biomolecules-16-00856-s001.zip › Western Blot Files/Figure 6 (LXR)/Fig6_MCF7TamR_Rep3_LXRb_RAW.tif]

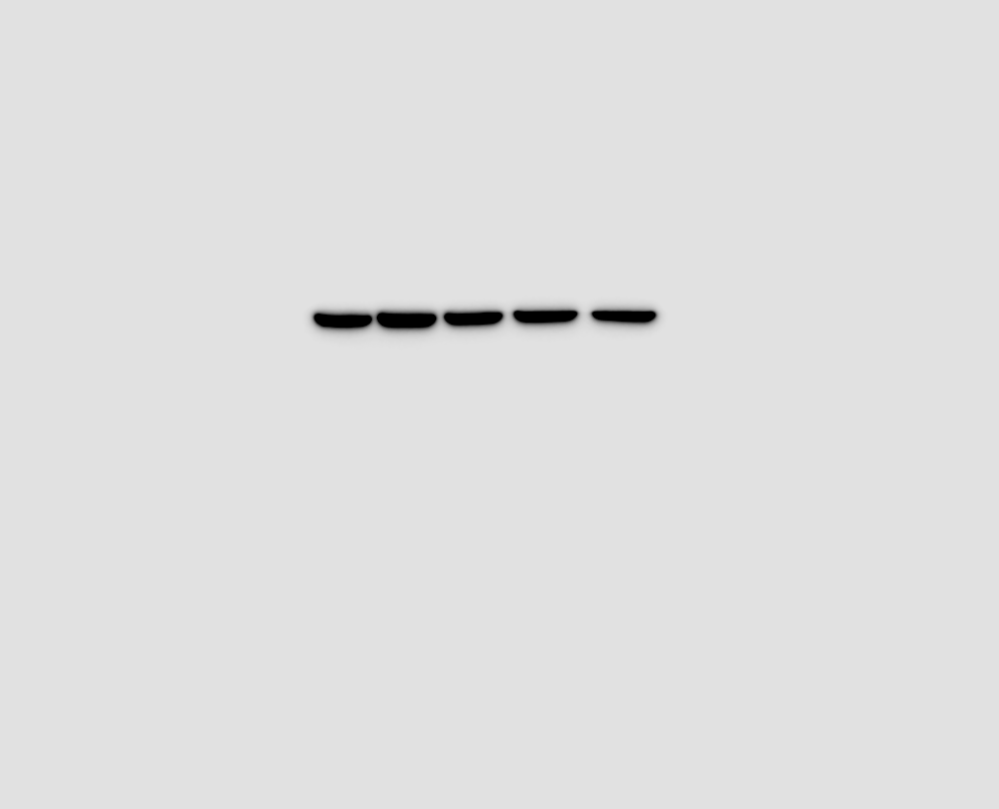

Supplement: Supplementary file 1 [file biomolecules-16-00856-s001.zip › Western Blot Files/Figure 6 (LXR)/Fig6_MCF7_Rep1_beta-actin_RAW.tif]

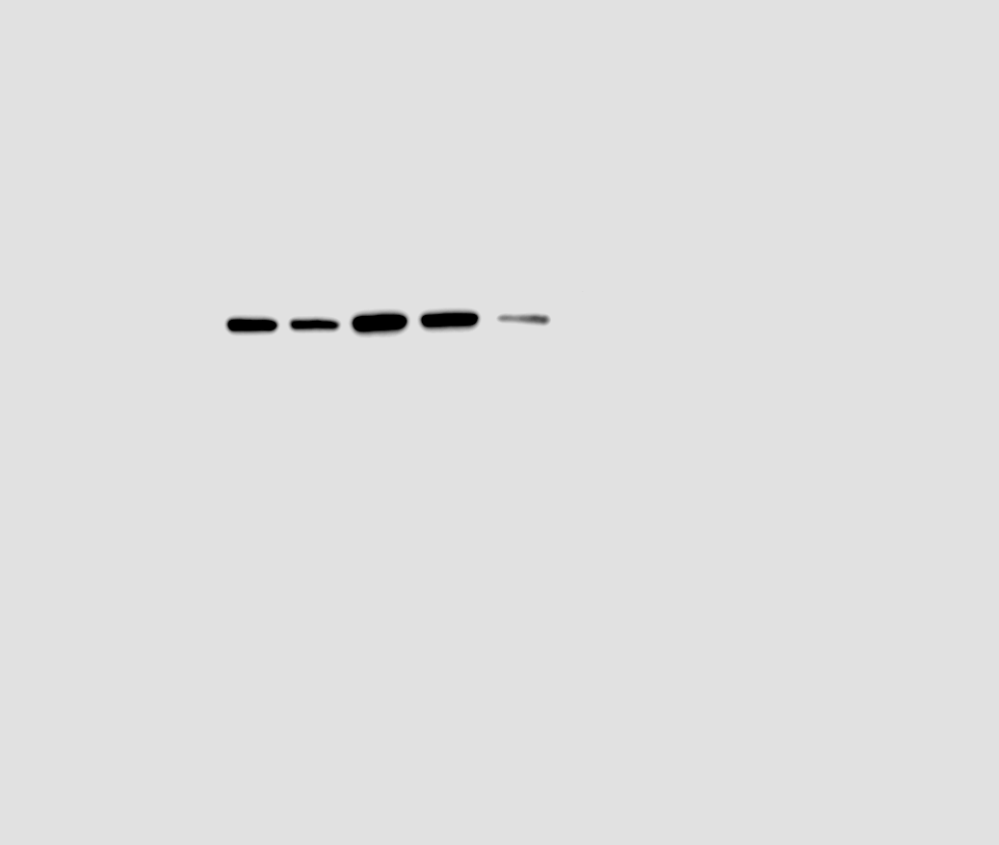

Supplement: Supplementary file 1 [file biomolecules-16-00856-s001.zip › Western Blot Files/Figure 6 (LXR)/Fig6_MCF7_Rep1_LXRb_RAW.tif]

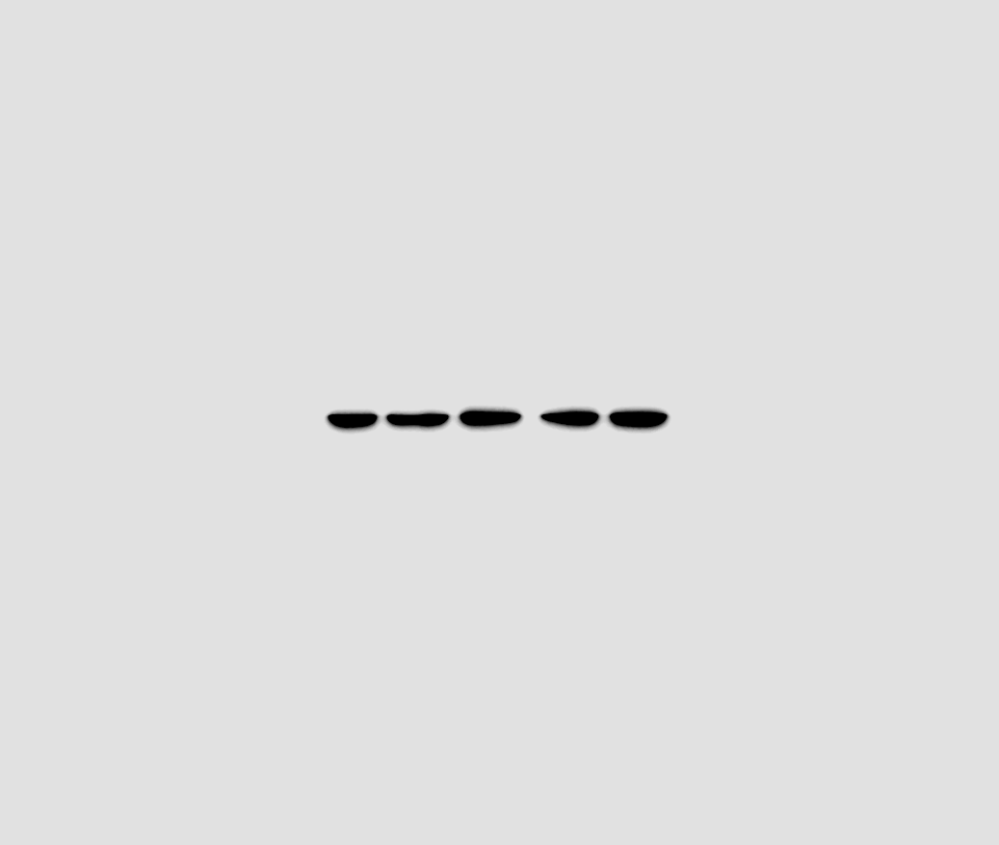

Supplement: Supplementary file 1 [file biomolecules-16-00856-s001.zip › Western Blot Files/Figure 6 (LXR)/Fig6_MCF7_Rep2_beta-actin_RAW.tif]

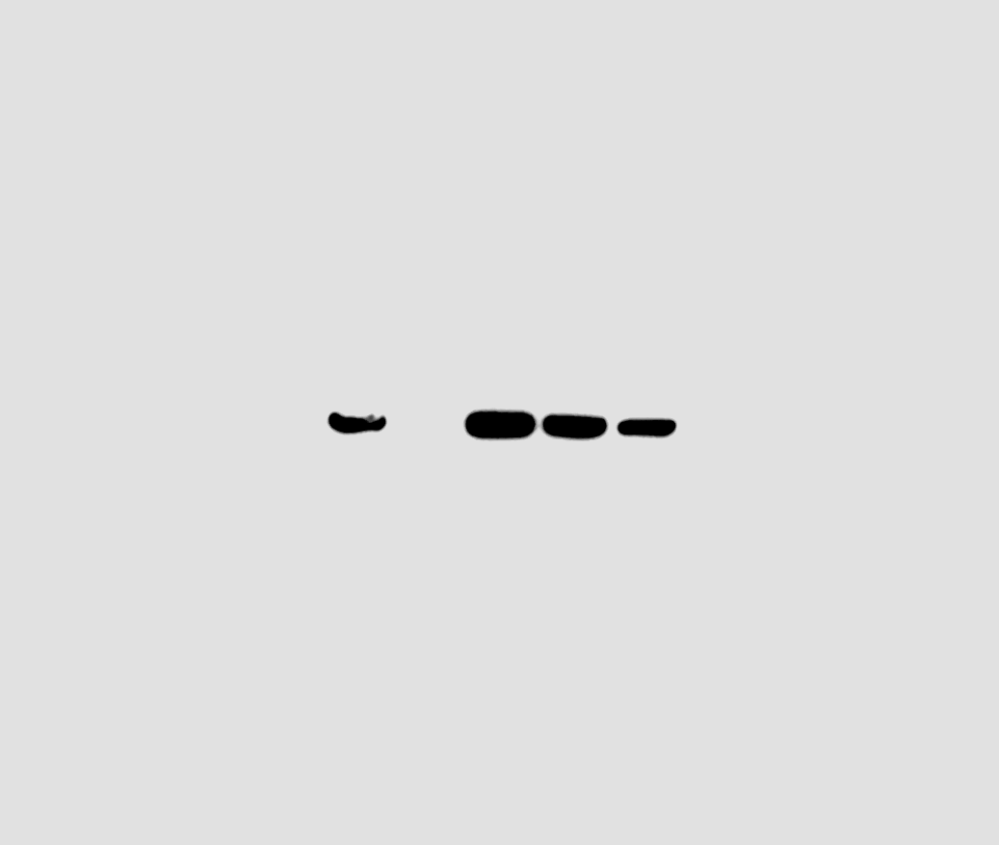

Supplement: Supplementary file 1 [file biomolecules-16-00856-s001.zip › Western Blot Files/Figure 6 (LXR)/Fig6_MCF7_Rep2_LXRb_RAW.tif]

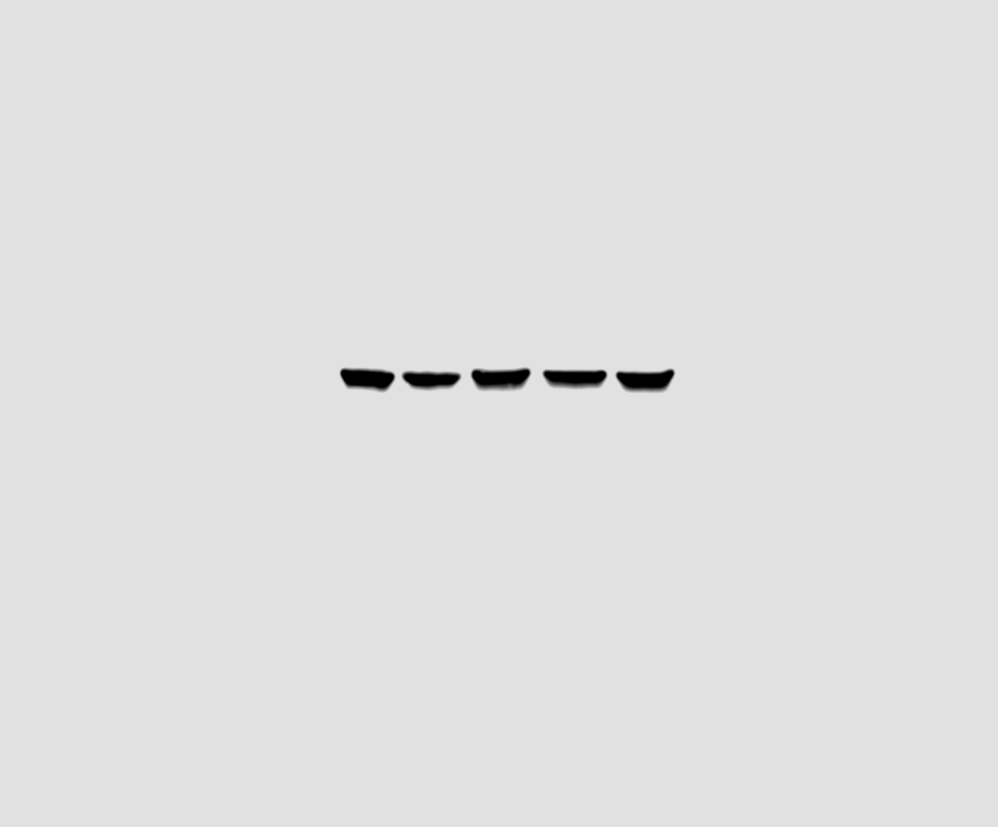

Supplement: Supplementary file 1 [file biomolecules-16-00856-s001.zip › Western Blot Files/Figure 6 (LXR)/Fig6_MCF7_Rep3_beta-actin_RAW.tif]

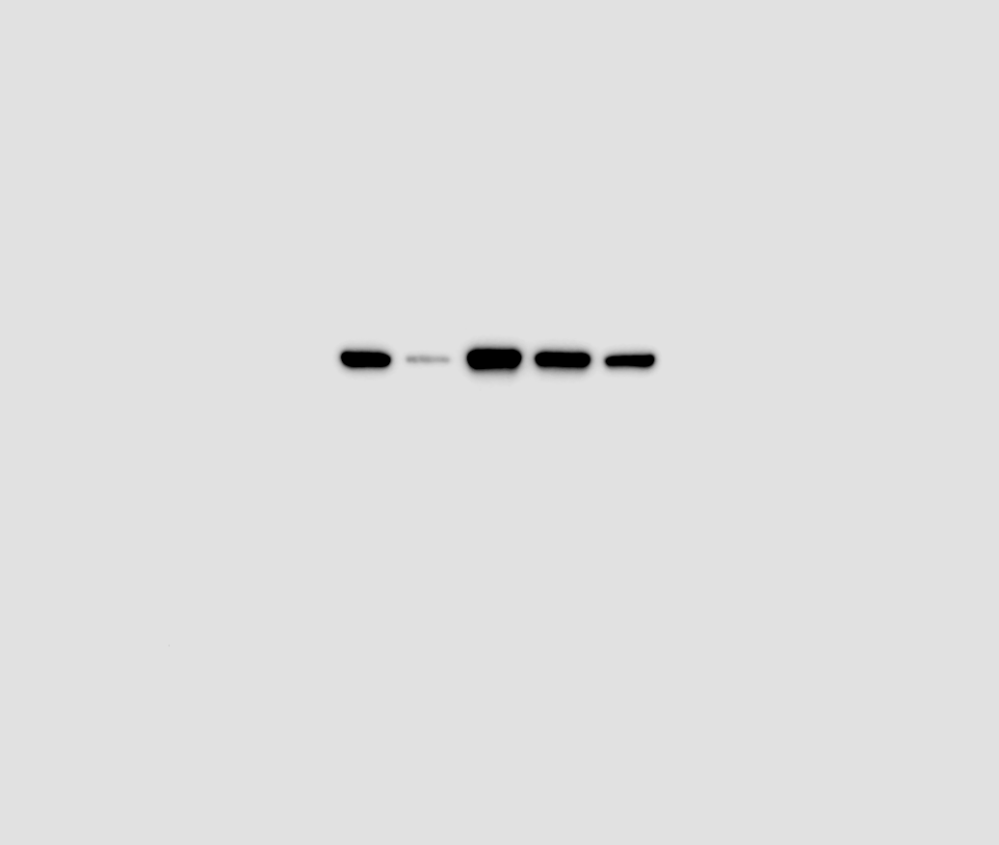

Supplement: Supplementary file 1 [file biomolecules-16-00856-s001.zip › Western Blot Files/Figure 6 (LXR)/Fig6_MCF7_Rep3_LXRb_RAW.tif]

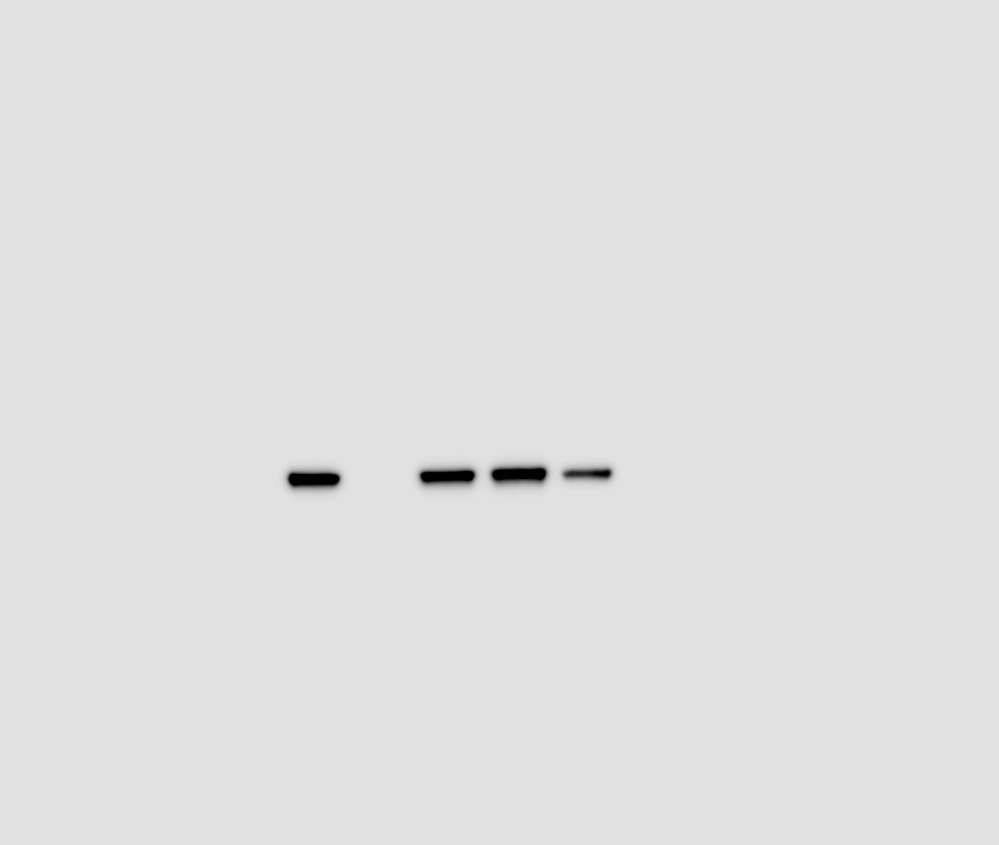

Supplement: Supplementary file 1 [file biomolecules-16-00856-s001.zip › Western Blot Files/Figure 7 (ER, AR, HER2)/Figure 7C (ER)/Fig7C_MCF7TamR_Rep1_ER_RAW.tif]

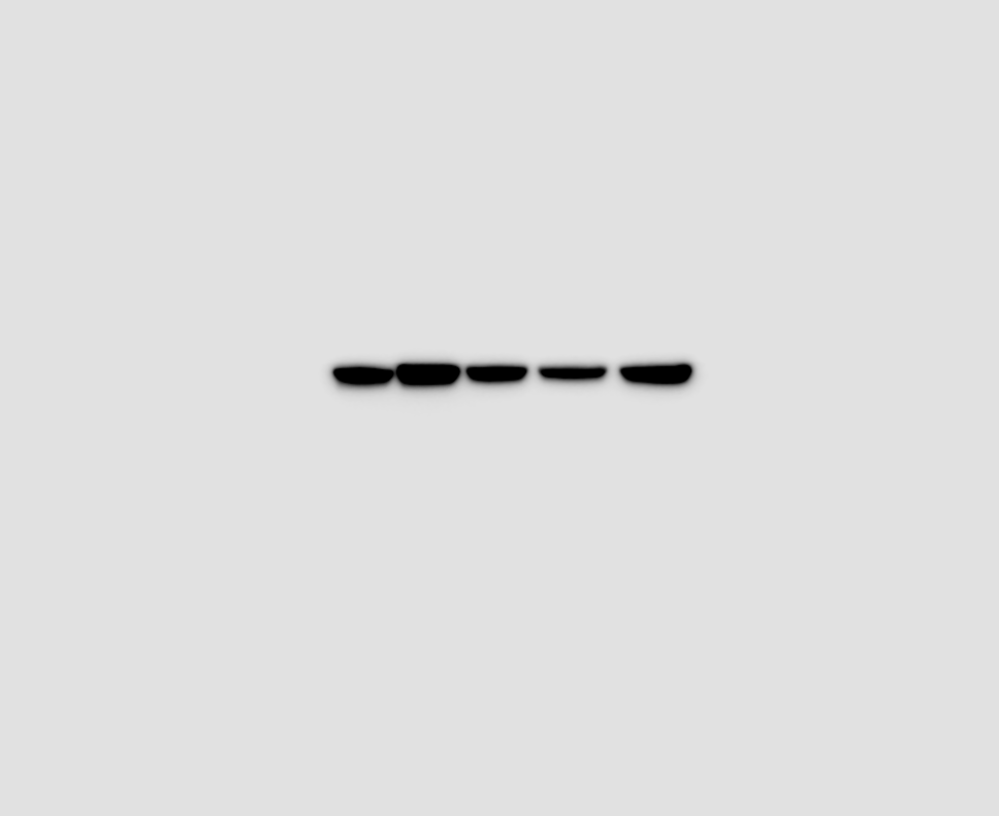

Supplement: Supplementary file 1 [file biomolecules-16-00856-s001.zip › Western Blot Files/Figure 7 (ER, AR, HER2)/Figure 7C (ER)/Fig7C_MCF7TamR_Rep2_beta-actin_RAW.tif]

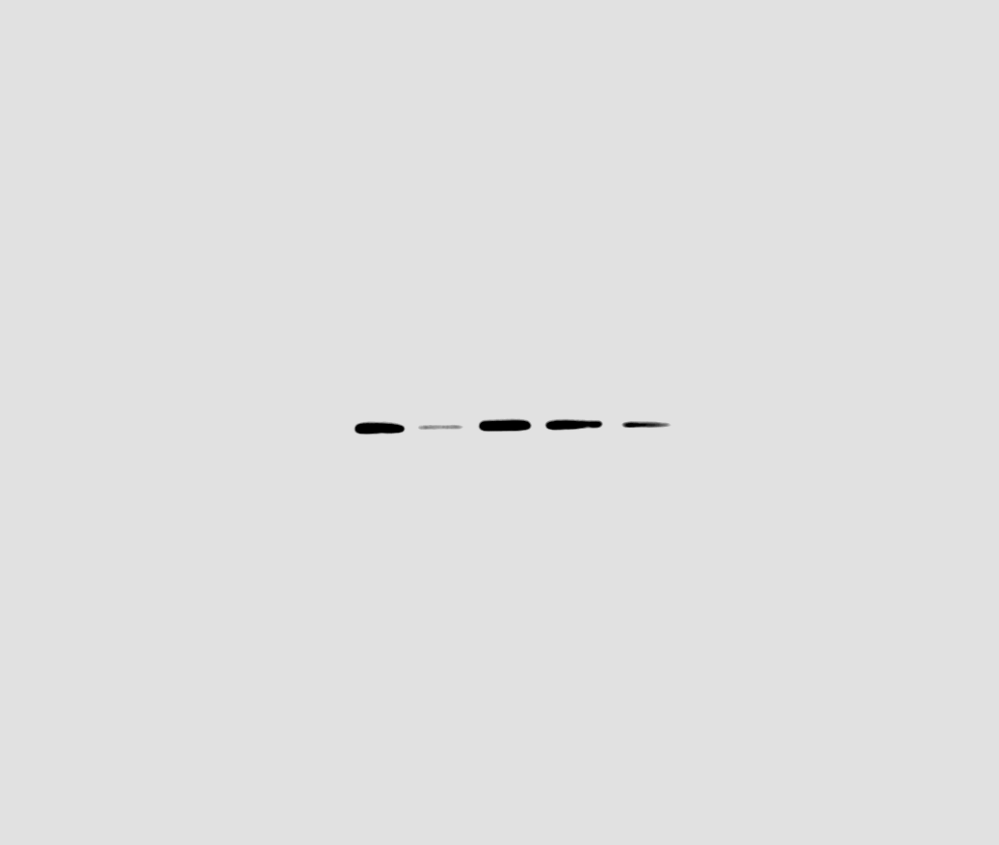

Supplement: Supplementary file 1 [file biomolecules-16-00856-s001.zip › Western Blot Files/Figure 7 (ER, AR, HER2)/Figure 7C (ER)/Fig7C_MCF7TamR_Rep2_ER_RAW.tif]

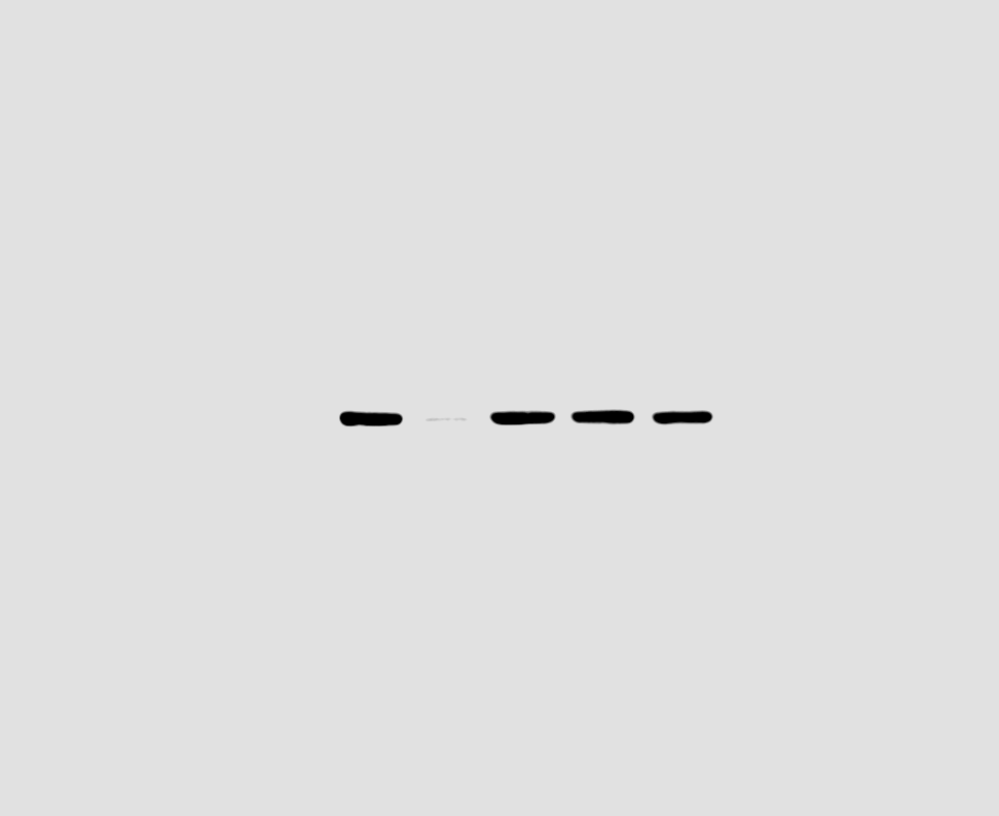

Supplement: Supplementary file 1 [file biomolecules-16-00856-s001.zip › Western Blot Files/Figure 7 (ER, AR, HER2)/Figure 7C (ER)/Fig7C_MCF7TamR_Rep3_ER_RAW.tif]

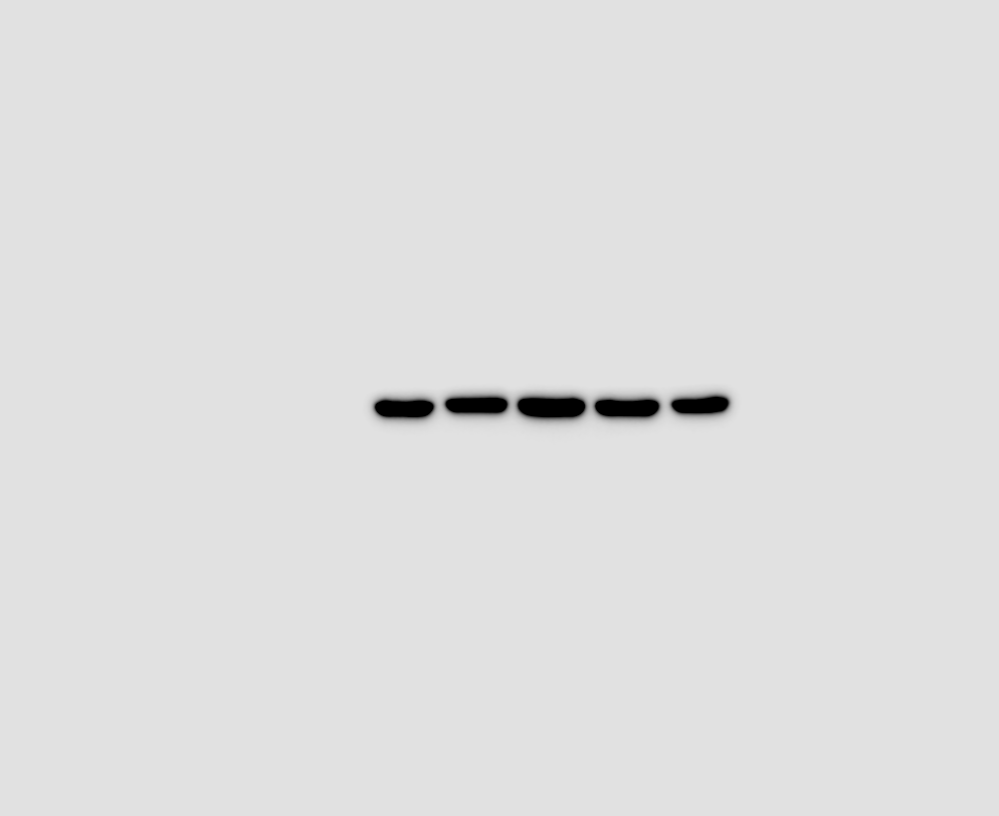

Supplement: Supplementary file 1 [file biomolecules-16-00856-s001.zip › Western Blot Files/Figure 7 (ER, AR, HER2)/Figure 7C (ER)/Fig7C_MCF7_Rep1_beta-actin_RAW.tif]

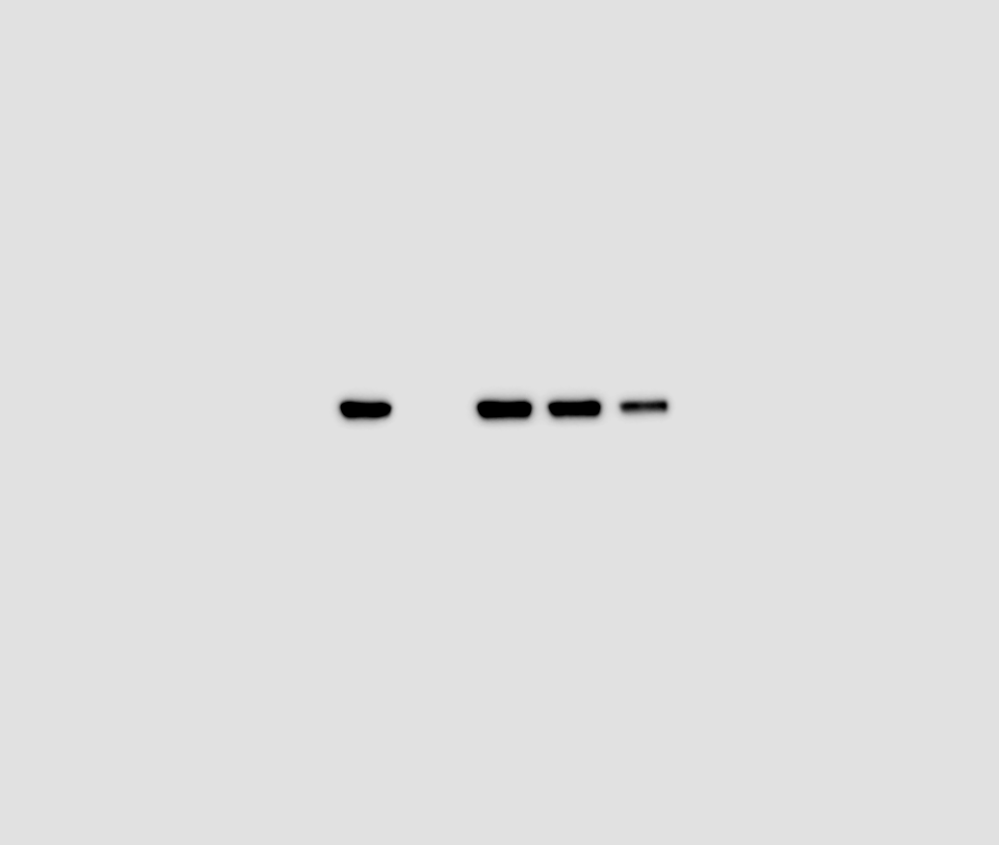

Supplement: Supplementary file 1 [file biomolecules-16-00856-s001.zip › Western Blot Files/Figure 7 (ER, AR, HER2)/Figure 7C (ER)/Fig7C_MCF7_Rep1_ER_RAW.tif]

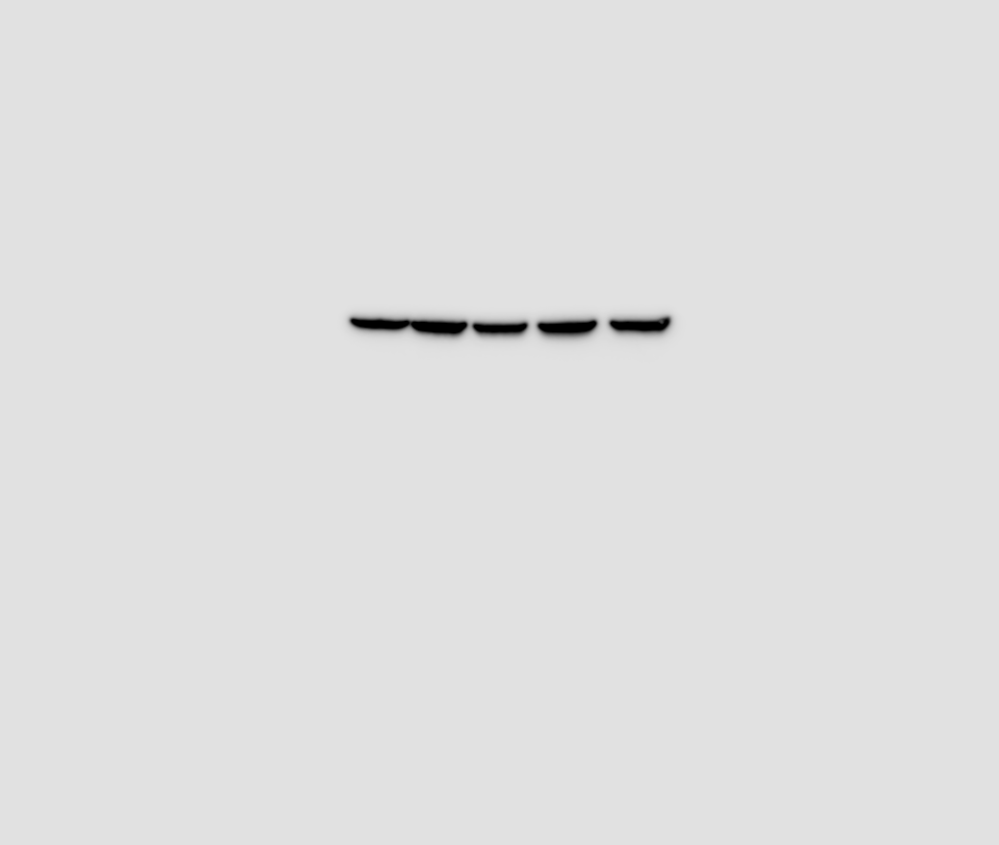

Supplement: Supplementary file 1 [file biomolecules-16-00856-s001.zip › Western Blot Files/Figure 7 (ER, AR, HER2)/Figure 7C (ER)/Fig7C_MCF7_Rep2_beta-actin_RAW.tif]

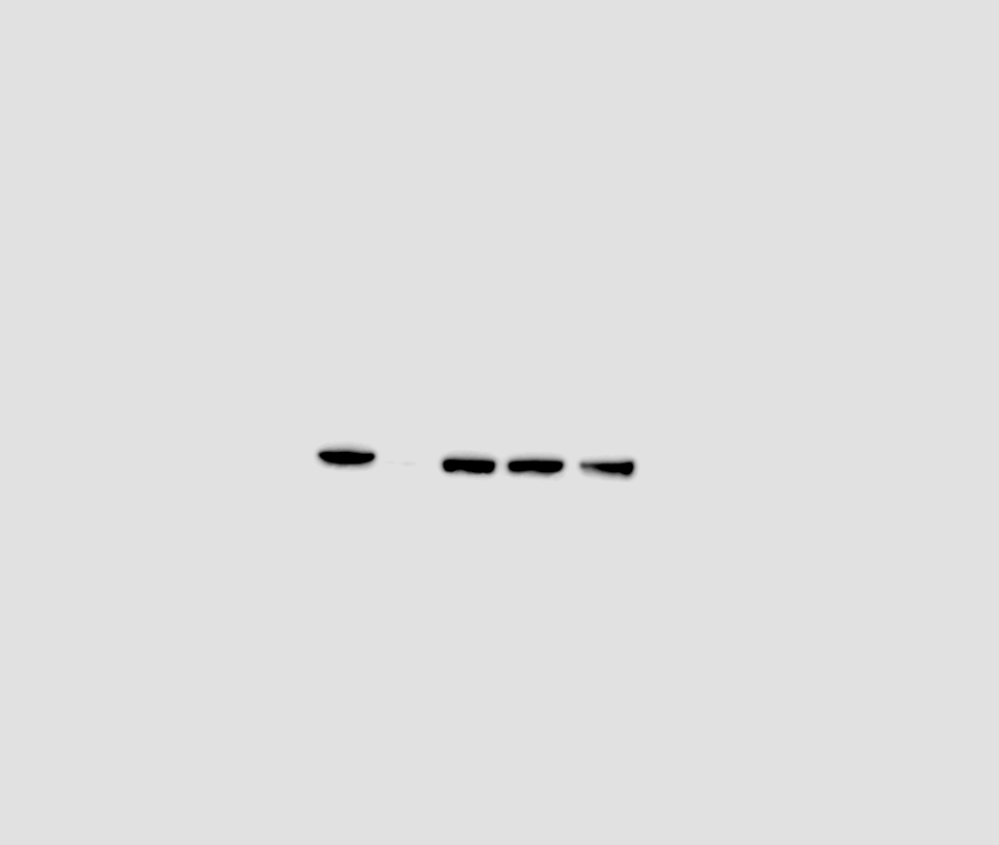

Supplement: Supplementary file 1 [file biomolecules-16-00856-s001.zip › Western Blot Files/Figure 7 (ER, AR, HER2)/Figure 7C (ER)/Fig7C_MCF7_Rep2_ER_RAW.tif]

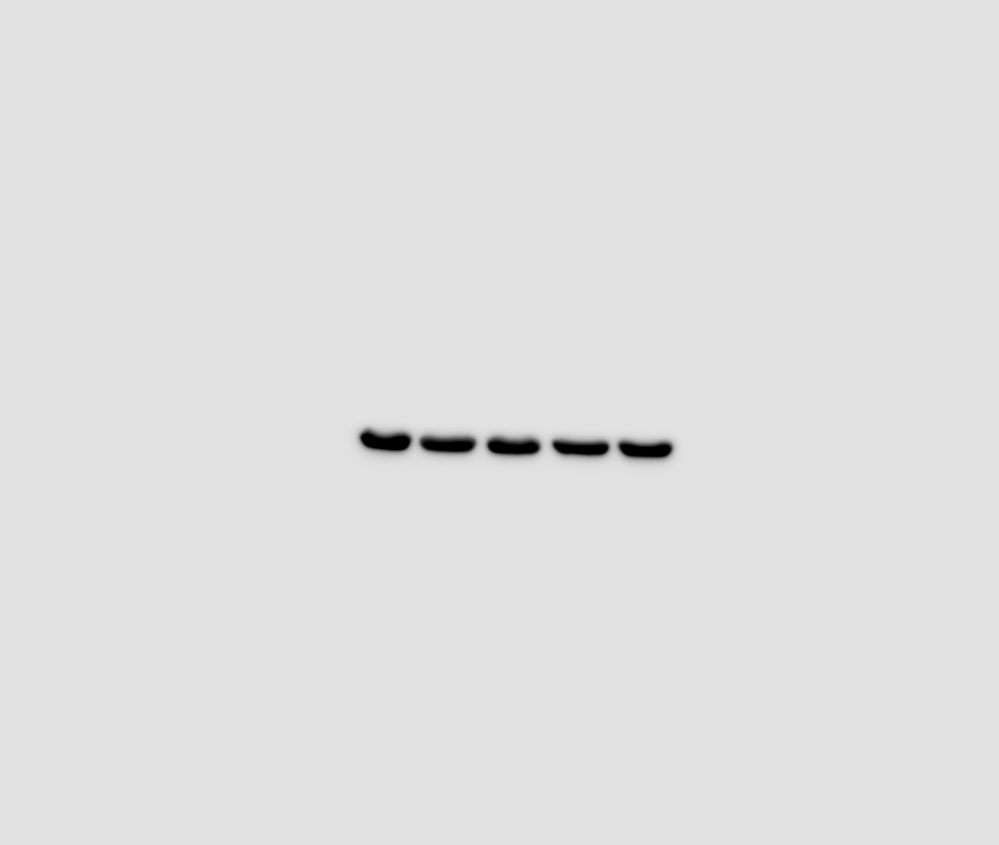

Supplement: Supplementary file 1 [file biomolecules-16-00856-s001.zip › Western Blot Files/Figure 7 (ER, AR, HER2)/Figure 7C (ER)/Fig7C_MCF7_Rep3_beta-actin_RAW.tif]

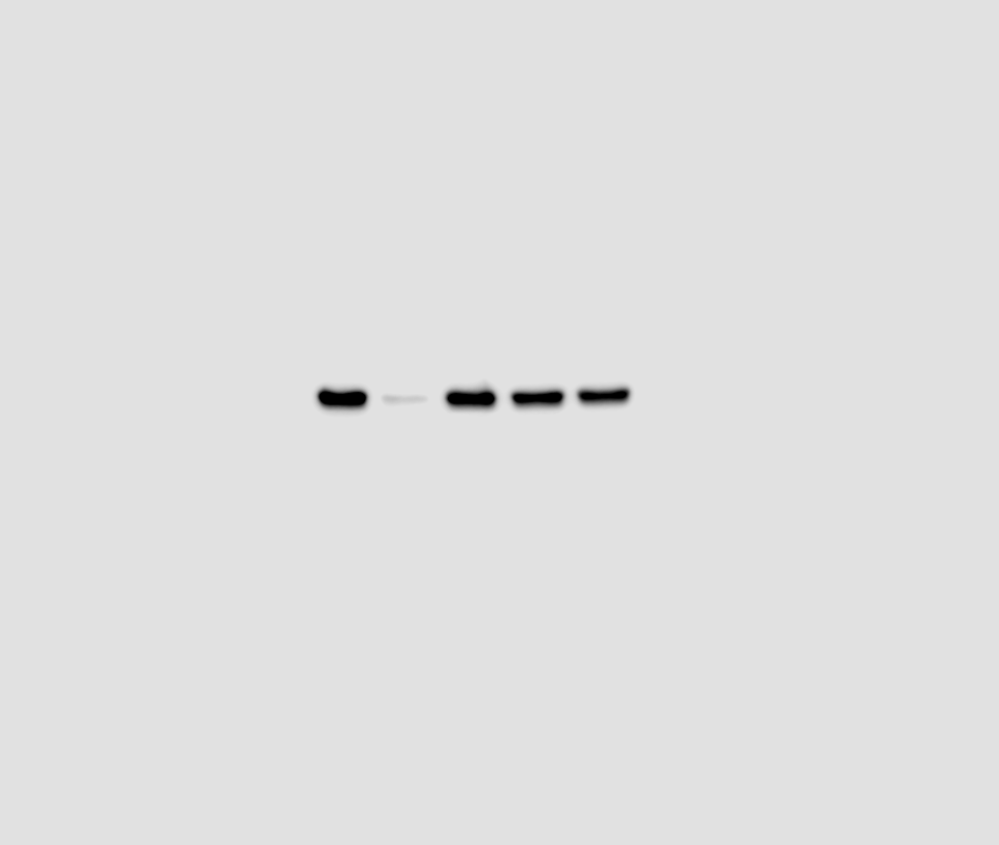

Supplement: Supplementary file 1 [file biomolecules-16-00856-s001.zip › Western Blot Files/Figure 7 (ER, AR, HER2)/Figure 7C (ER)/Fig7C_MCF7_Rep3_ER_RAW.tif]

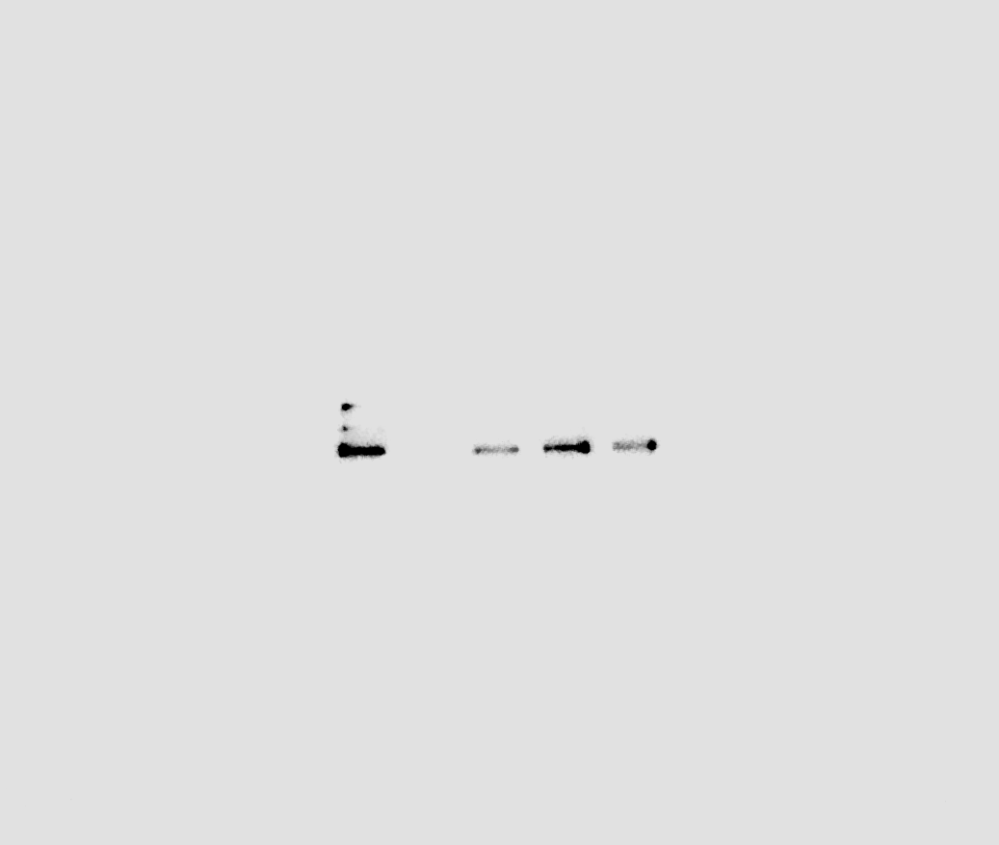

Supplement: Supplementary file 1 [file biomolecules-16-00856-s001.zip › Western Blot Files/Figure 7 (ER, AR, HER2)/Figure 7F (AR)/Fig7F_MCF7TamR_Rep1_AR_RAW_restored.tif]

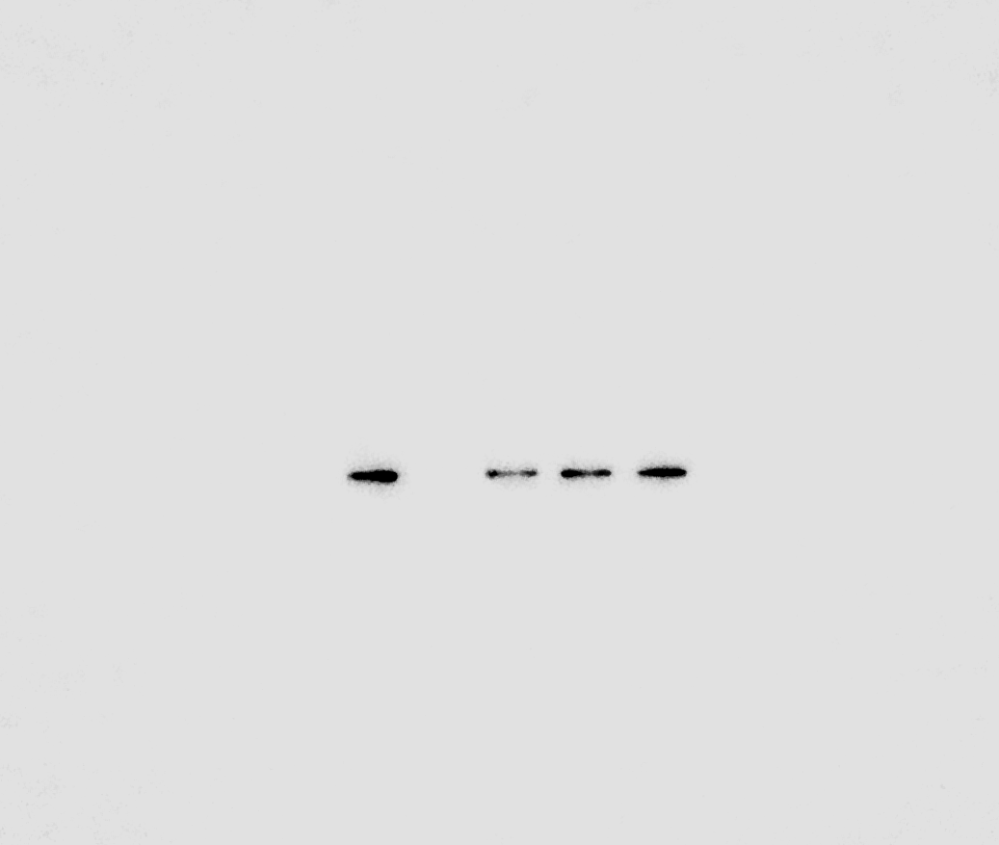

Supplement: Supplementary file 1 [file biomolecules-16-00856-s001.zip › Western Blot Files/Figure 7 (ER, AR, HER2)/Figure 7F (AR)/Fig7F_MCF7TamR_Rep2_AR_RAW.tif]

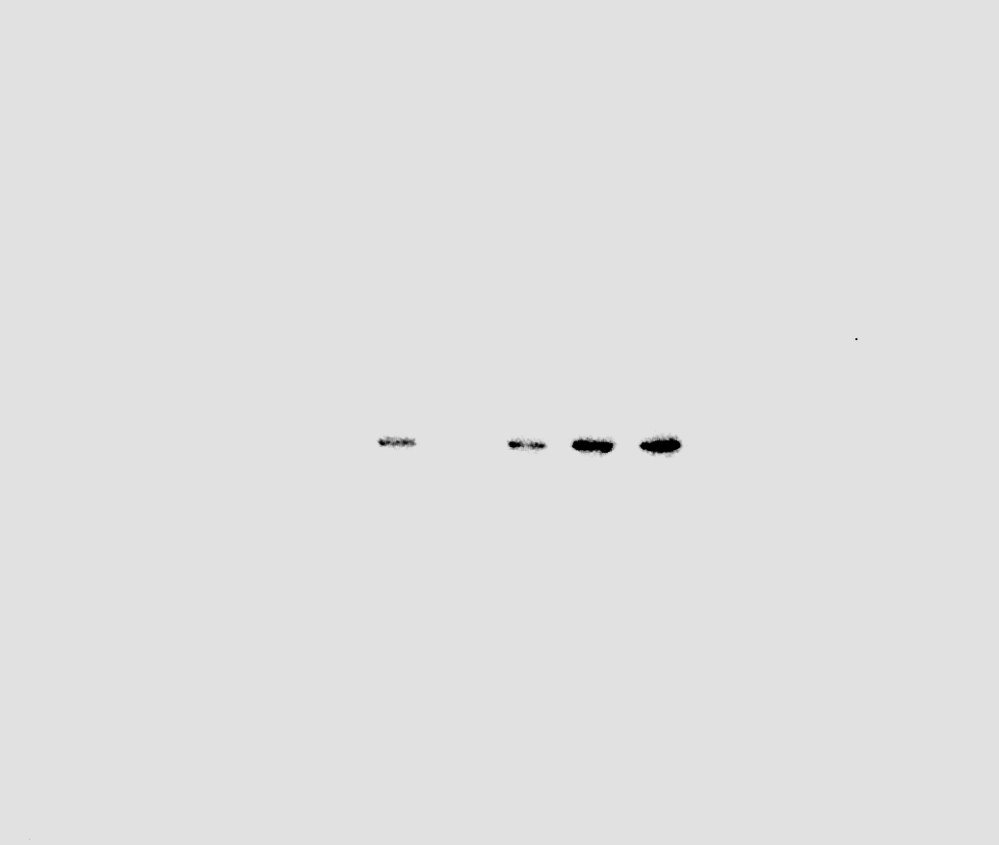

Supplement: Supplementary file 1 [file biomolecules-16-00856-s001.zip › Western Blot Files/Figure 7 (ER, AR, HER2)/Figure 7F (AR)/Fig7F_MCF7TamR_Rep3_AR_RAW.tif]

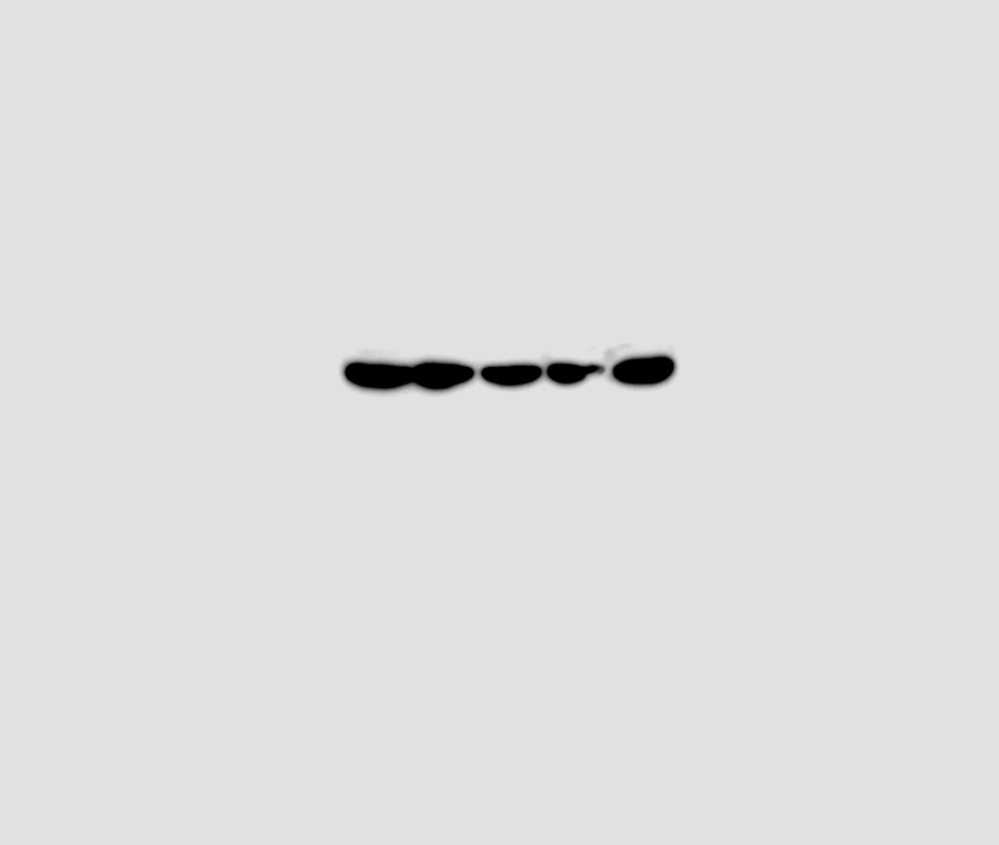

Supplement: Supplementary file 1 [file biomolecules-16-00856-s001.zip › Western Blot Files/Figure 7 (ER, AR, HER2)/Figure 7F (AR)/Fig7F_MCF7TamR_Rep3_beta-actin_RAW.tif]

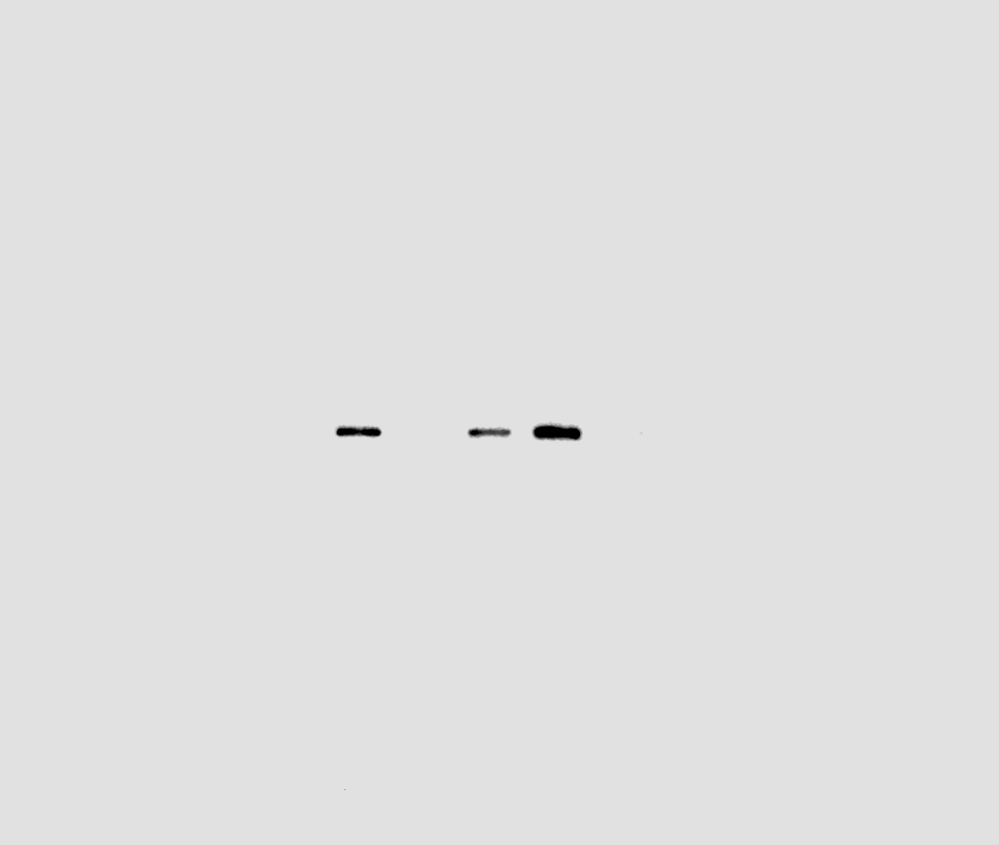

Supplement: Supplementary file 1 [file biomolecules-16-00856-s001.zip › Western Blot Files/Figure 7 (ER, AR, HER2)/Figure 7I (HER2)/Fig7I_MCF7TamR_Rep1_HER2_RAW.tif]

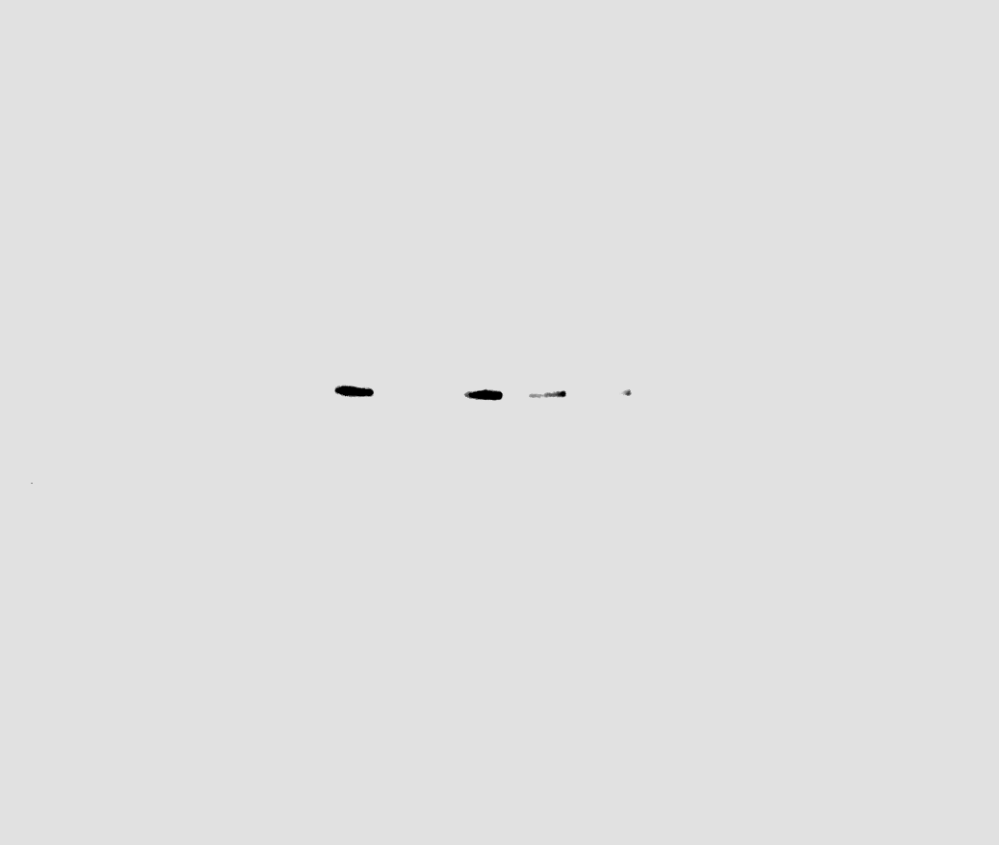

Supplement: Supplementary file 1 [file biomolecules-16-00856-s001.zip › Western Blot Files/Figure 7 (ER, AR, HER2)/Figure 7I (HER2)/Fig7I_MCF7TamR_Rep2_HER2_RAW.tif]

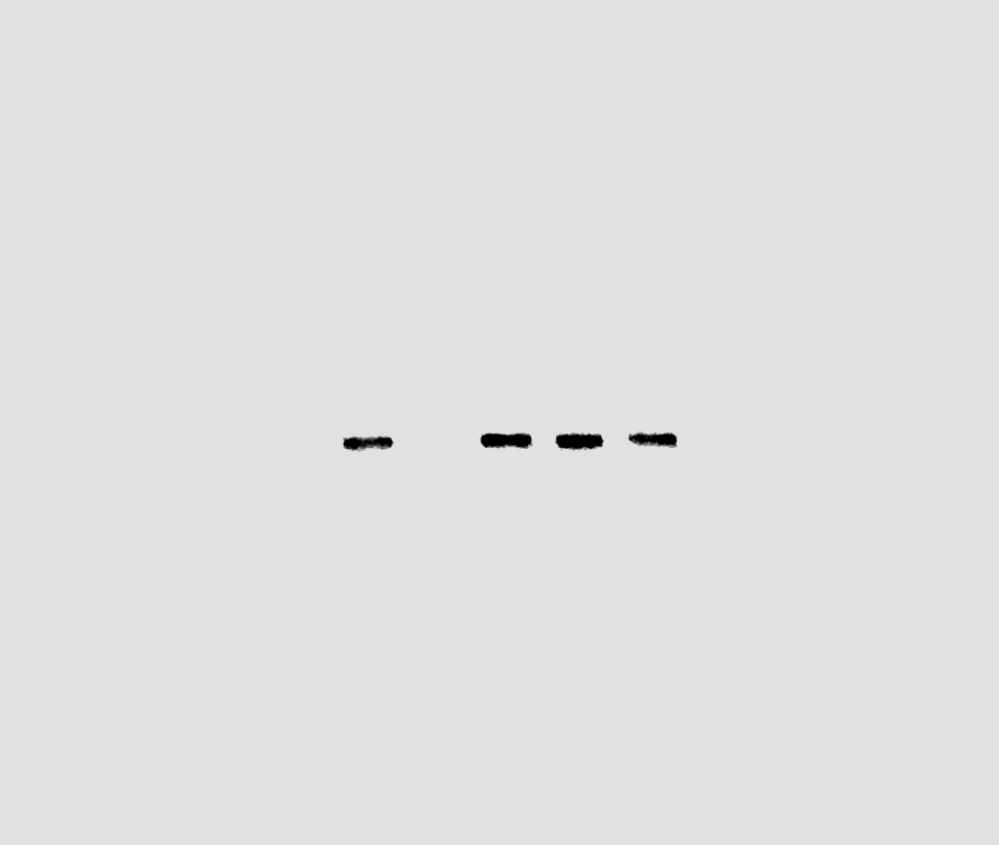

Supplement: Supplementary file 1 [file biomolecules-16-00856-s001.zip › Western Blot Files/Figure 7 (ER, AR, HER2)/Figure 7I (HER2)/Fig7I_MCF7TamR_Rep3_HER2_RAW.tif]

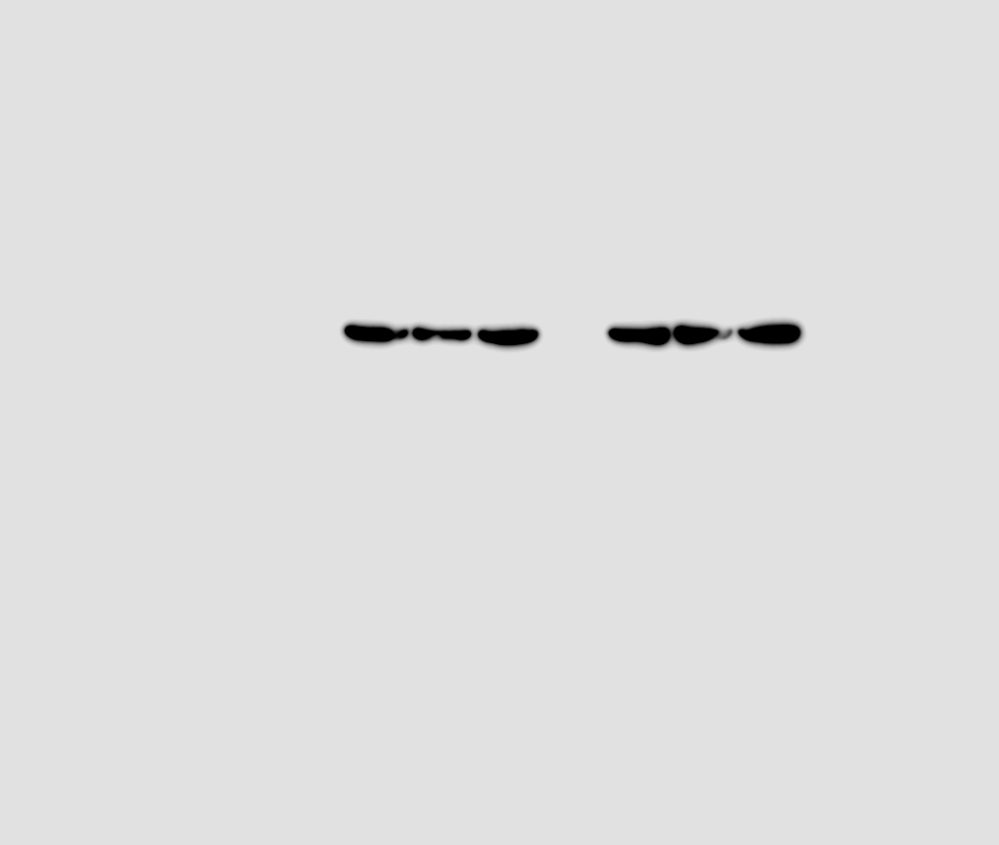

Supplement: Supplementary file 1 [file biomolecules-16-00856-s001.zip › Western Blot Files/Figure 8 (LXR Knockdown)/Figure 8C LXR Knockdown/Fig8C_MCF7TamR_Rep1,2_beta-actin_RAW.tif]

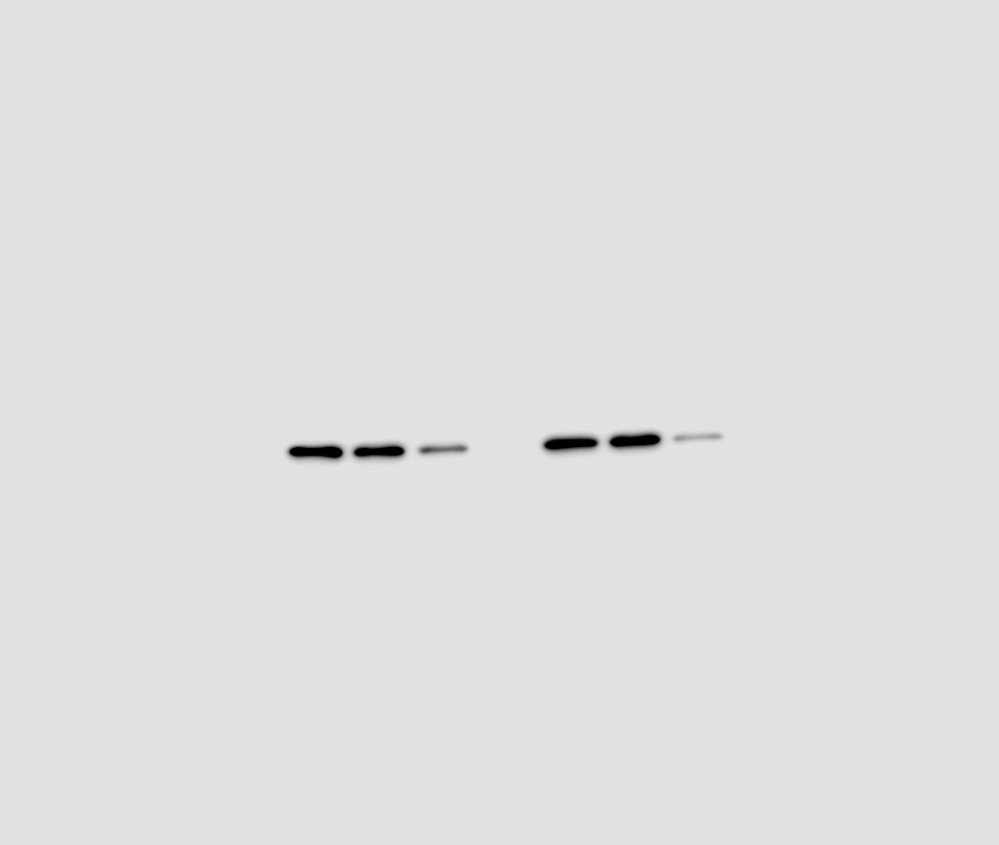

Supplement: Supplementary file 1 [file biomolecules-16-00856-s001.zip › Western Blot Files/Figure 8 (LXR Knockdown)/Figure 8C LXR Knockdown/Fig8C_MCF7TamR_Rep1,2_LXRb_RAW.tif]

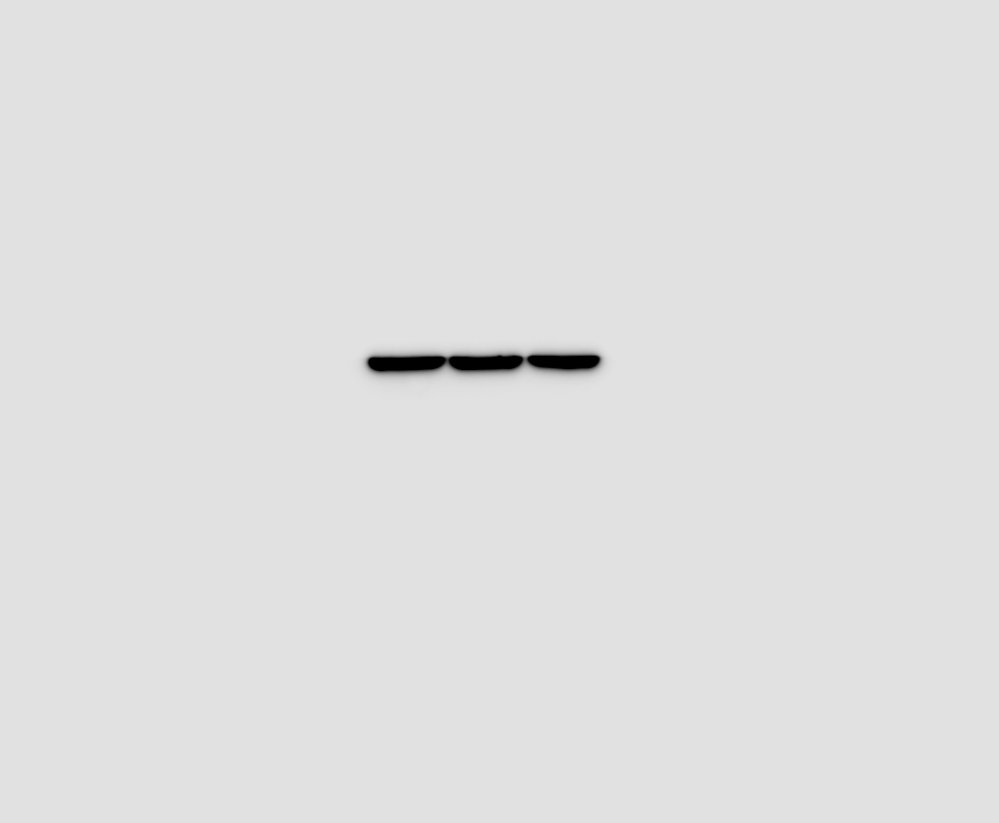

Supplement: Supplementary file 1 [file biomolecules-16-00856-s001.zip › Western Blot Files/Figure 8 (LXR Knockdown)/Figure 8C LXR Knockdown/Fig8C_MCF7TamR_Rep3_beta-actin_RAW.tif]

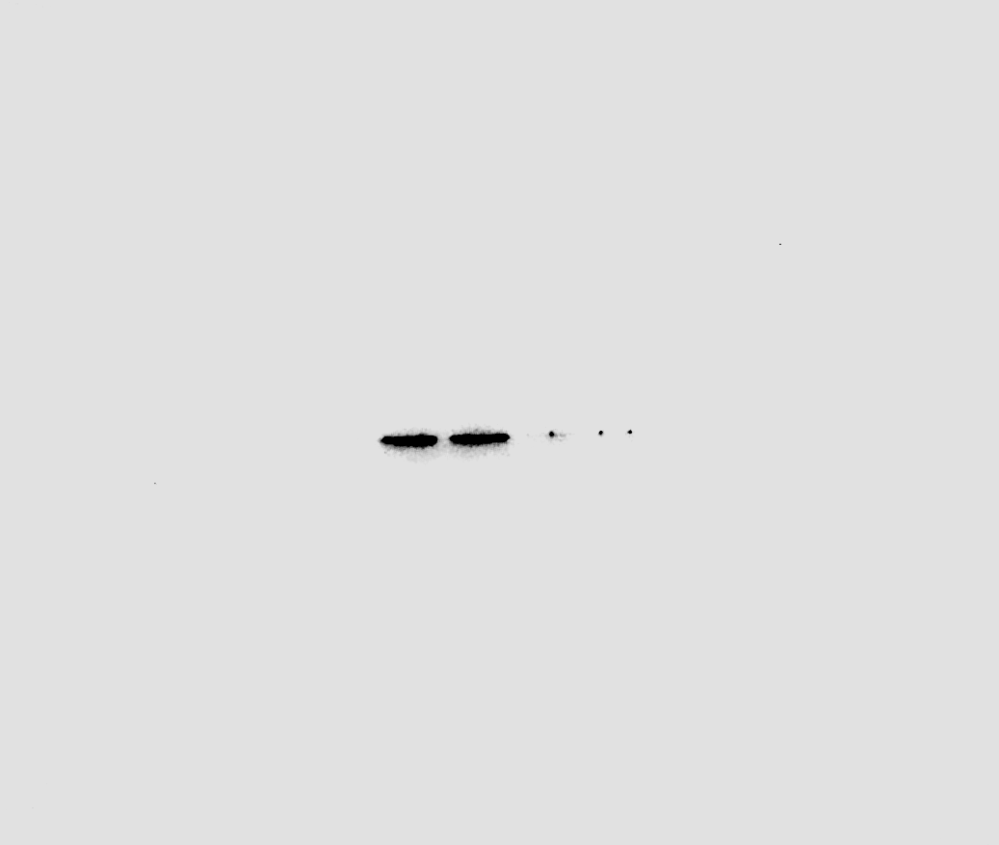

Supplement: Supplementary file 1 [file biomolecules-16-00856-s001.zip › Western Blot Files/Figure 8 (LXR Knockdown)/Figure 8C LXR Knockdown/Fig8C_MCF7TamR_Rep3_LXRb_RAW.tif]

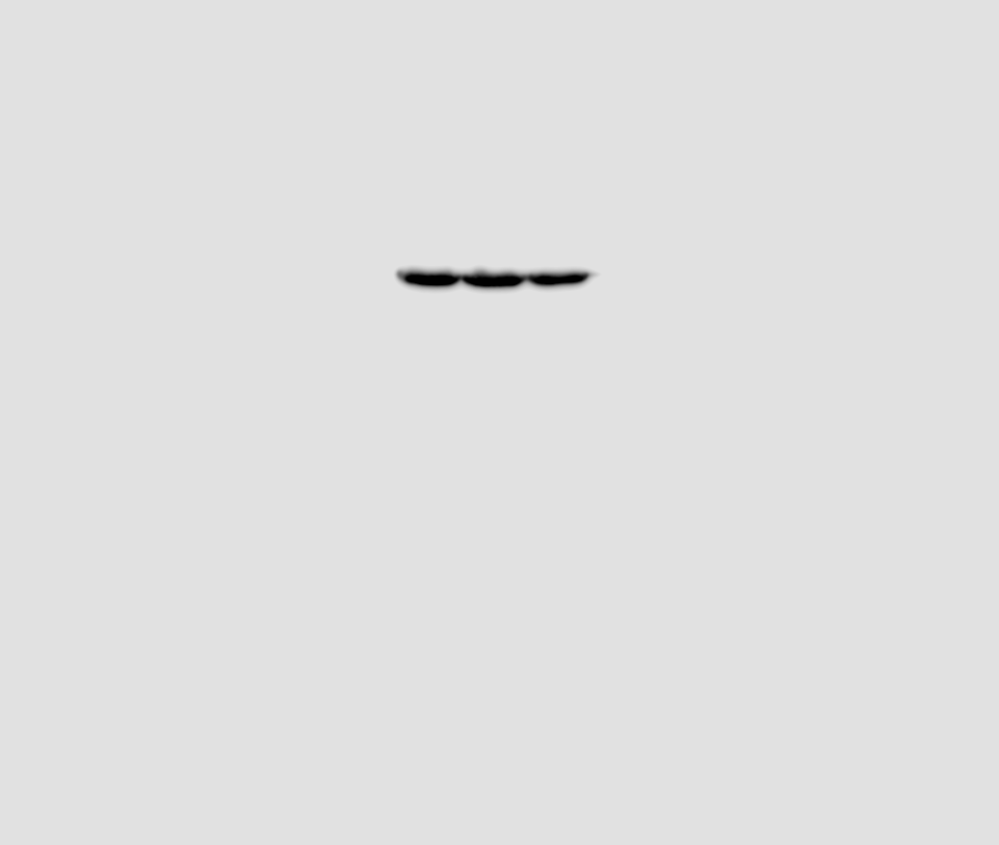

Supplement: Supplementary file 1 [file biomolecules-16-00856-s001.zip › Western Blot Files/Figure 8 (LXR Knockdown)/Figure 8C LXR Knockdown/Fig8C_MCF7_Rep1_beta-actin_RAW.tif]

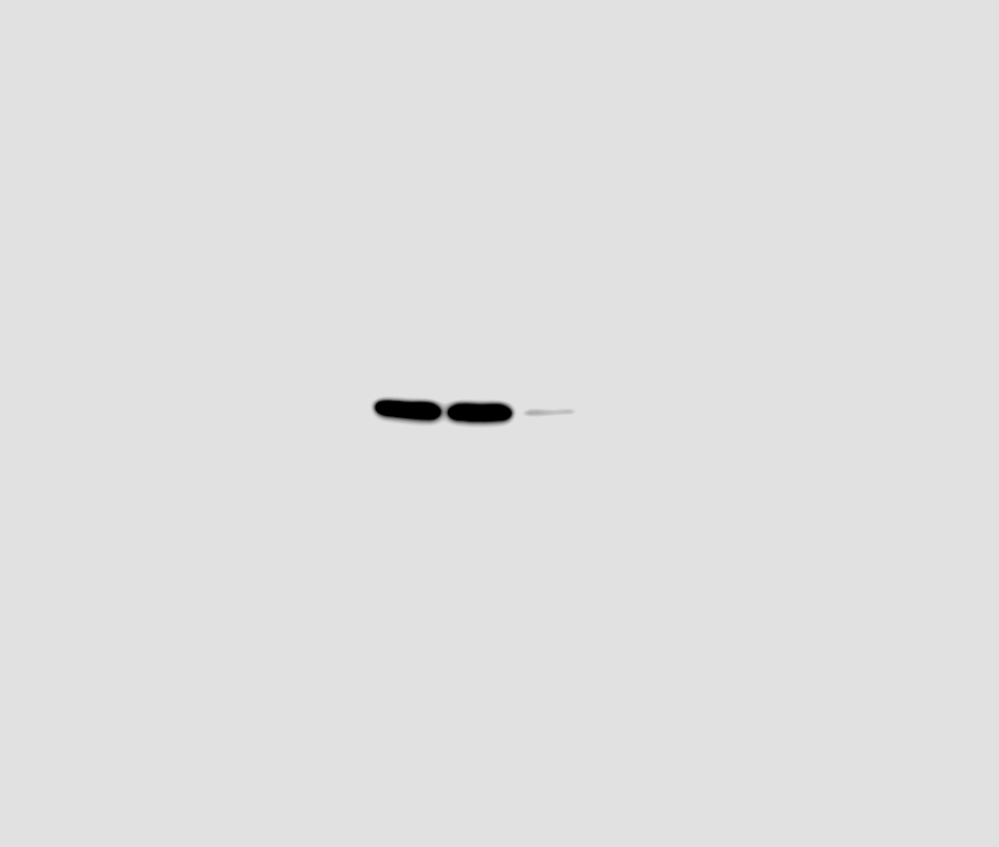

Supplement: Supplementary file 1 [file biomolecules-16-00856-s001.zip › Western Blot Files/Figure 8 (LXR Knockdown)/Figure 8C LXR Knockdown/Fig8C_MCF7_Rep1_LXRb_RAW.tif]

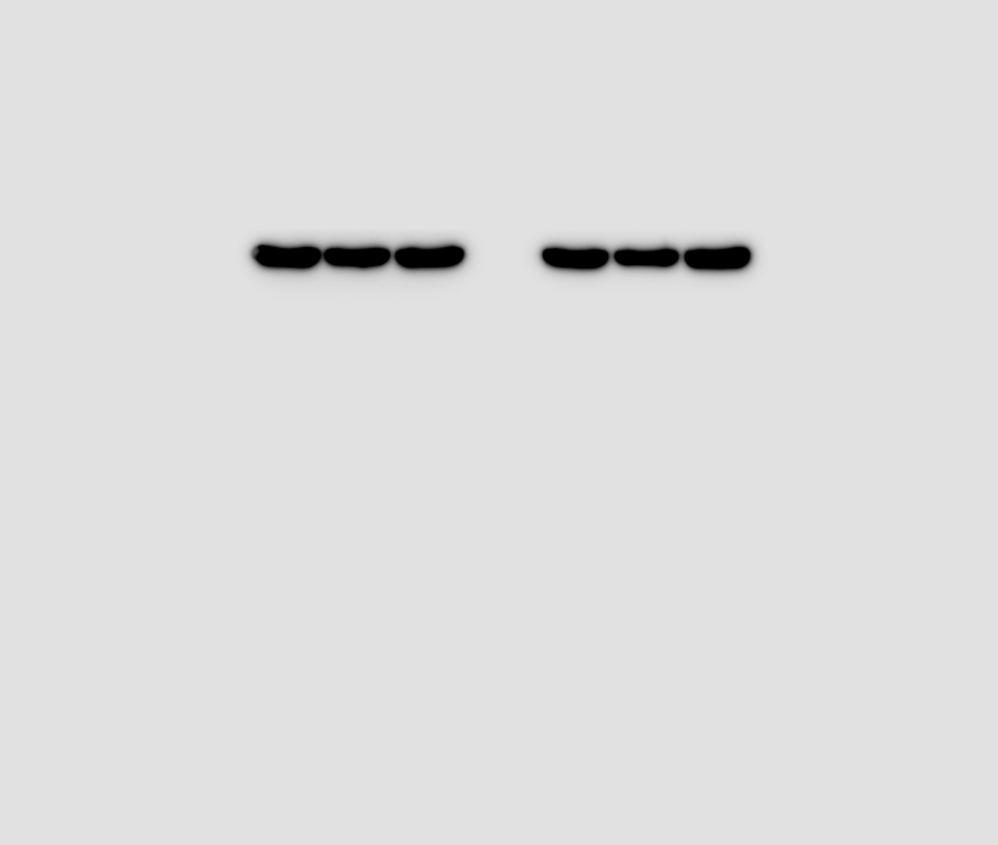

Supplement: Supplementary file 1 [file biomolecules-16-00856-s001.zip › Western Blot Files/Figure 8 (LXR Knockdown)/Figure 8C LXR Knockdown/Fig8C_MCF7_Rep2,3_beta-actin_RAW.tif]

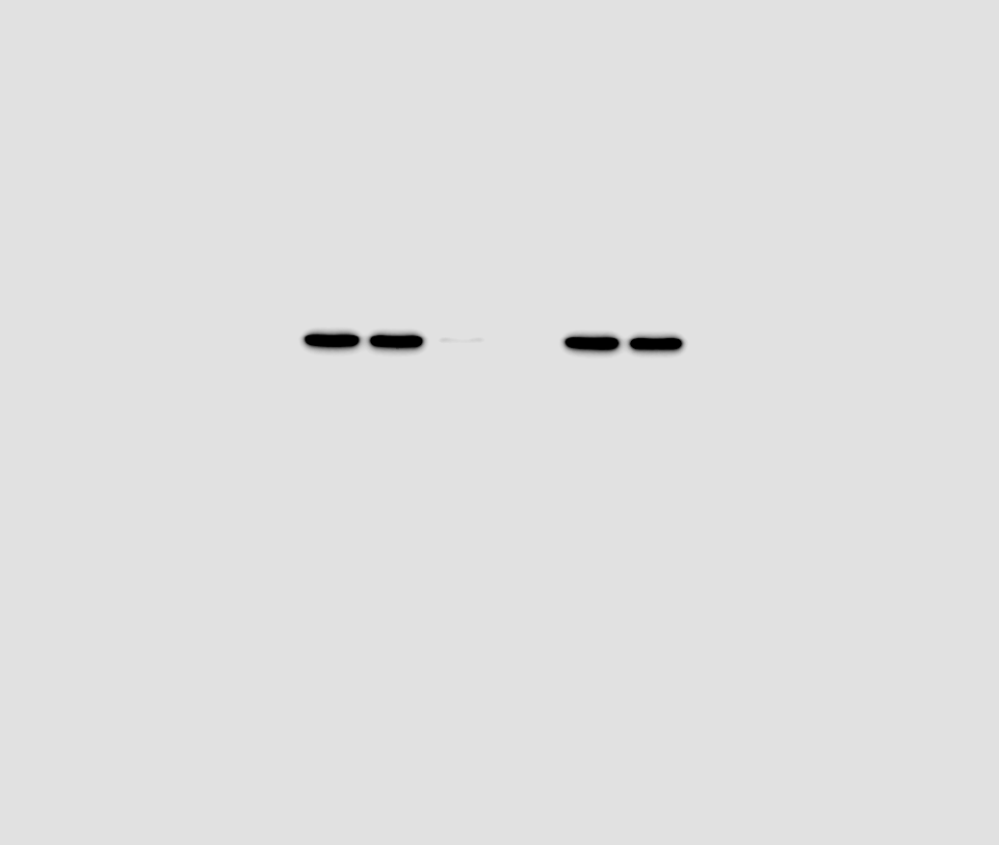

Supplement: Supplementary file 1 [file biomolecules-16-00856-s001.zip › Western Blot Files/Figure 8 (LXR Knockdown)/Figure 8C LXR Knockdown/Fig8C_MCF7_Rep2,3_LXRb_RAW.tif]

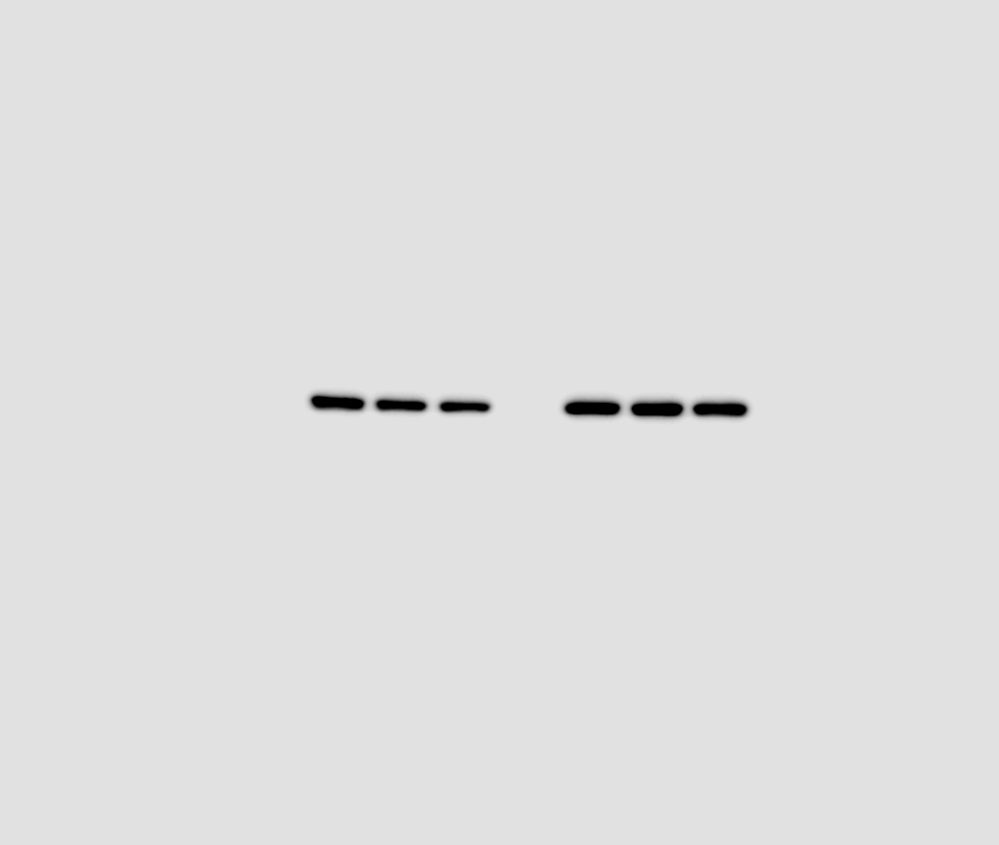

Supplement: Supplementary file 1 [file biomolecules-16-00856-s001.zip › Western Blot Files/Figure 8 (LXR Knockdown)/Figure 8F ER (post LXR knockdown)/Fig8F_MCF7TamR_Rep1,2_ER_RAW.tif]

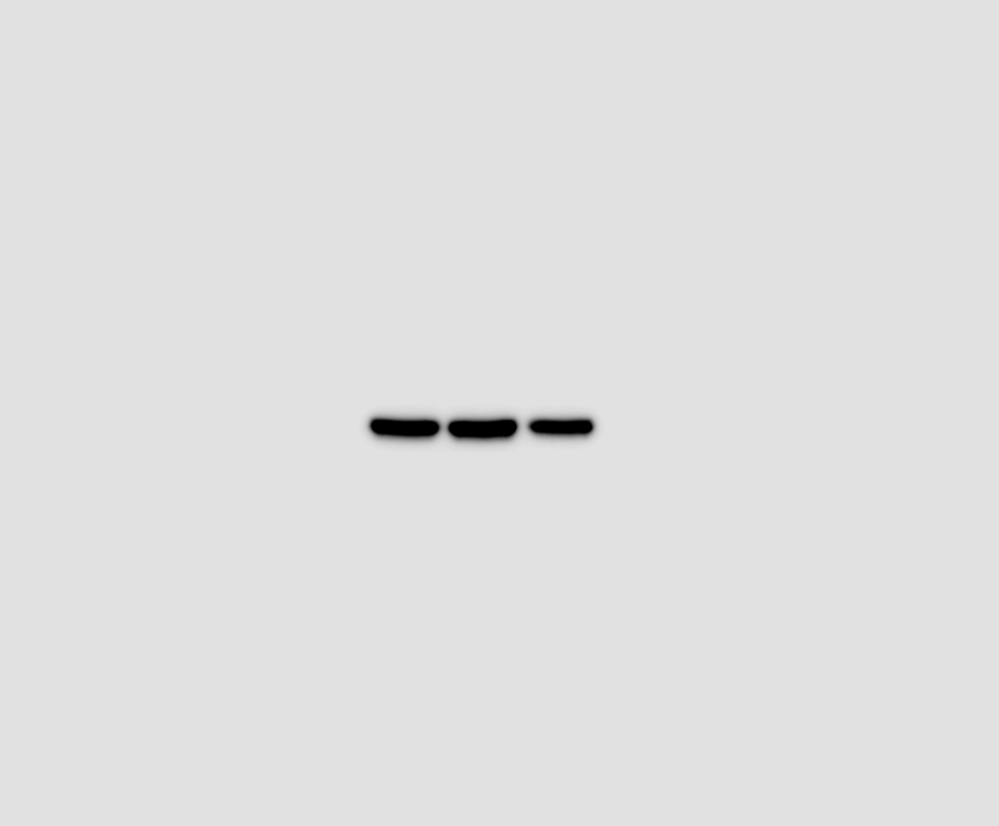

Supplement: Supplementary file 1 [file biomolecules-16-00856-s001.zip › Western Blot Files/Figure 8 (LXR Knockdown)/Figure 8F ER (post LXR knockdown)/Fig8F_MCF7TamR_Rep3_ER_RAW.tif]

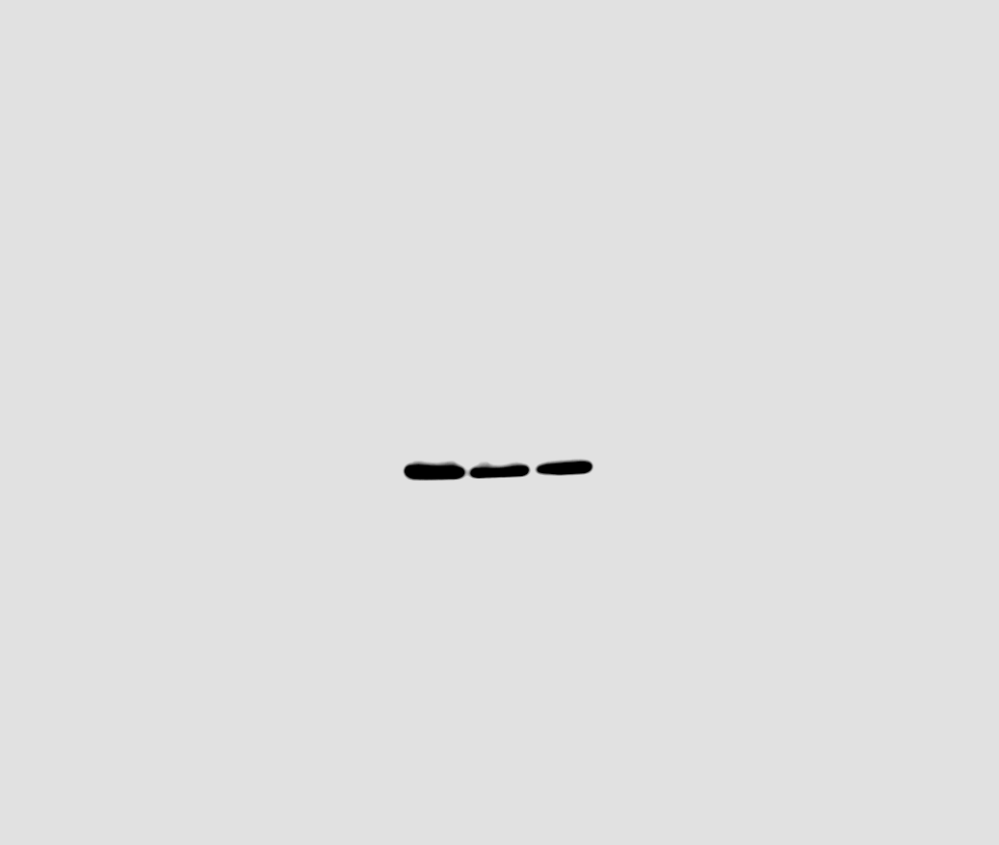

Supplement: Supplementary file 1 [file biomolecules-16-00856-s001.zip › Western Blot Files/Figure 8 (LXR Knockdown)/Figure 8F ER (post LXR knockdown)/Fig8F_MCF7_Rep1_ER_RAW.tif]

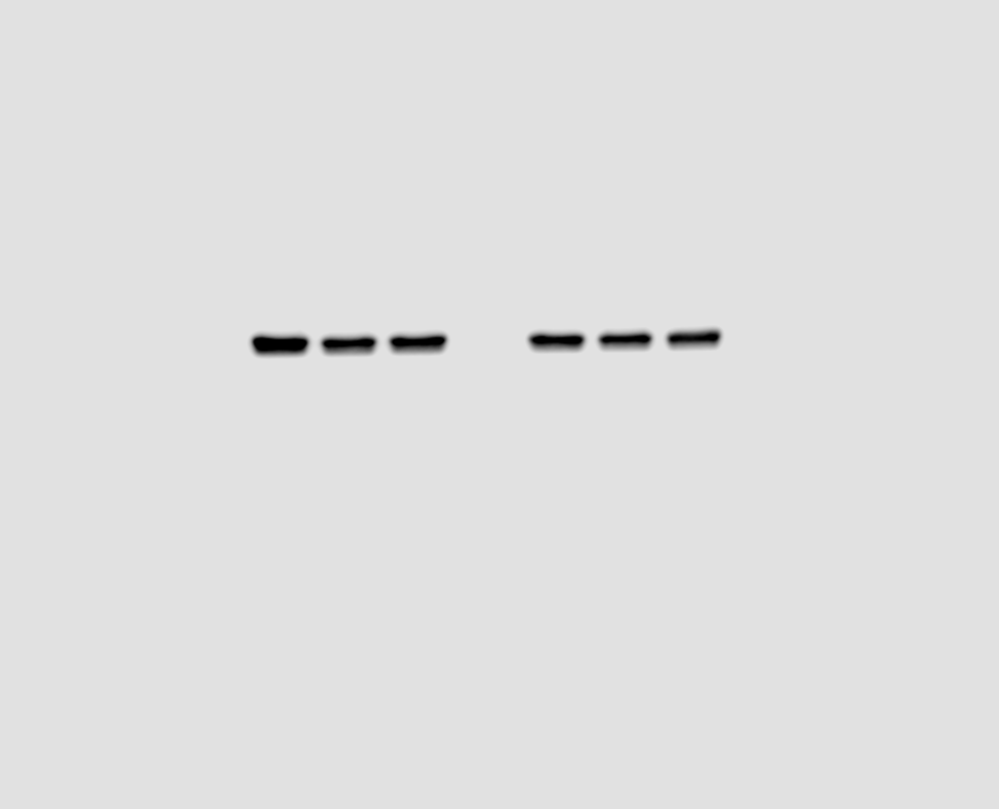

Supplement: Supplementary file 1 [file biomolecules-16-00856-s001.zip › Western Blot Files/Figure 8 (LXR Knockdown)/Figure 8F ER (post LXR knockdown)/Fig8F_MCF7_Rep2,3_ER_RAW.tif]

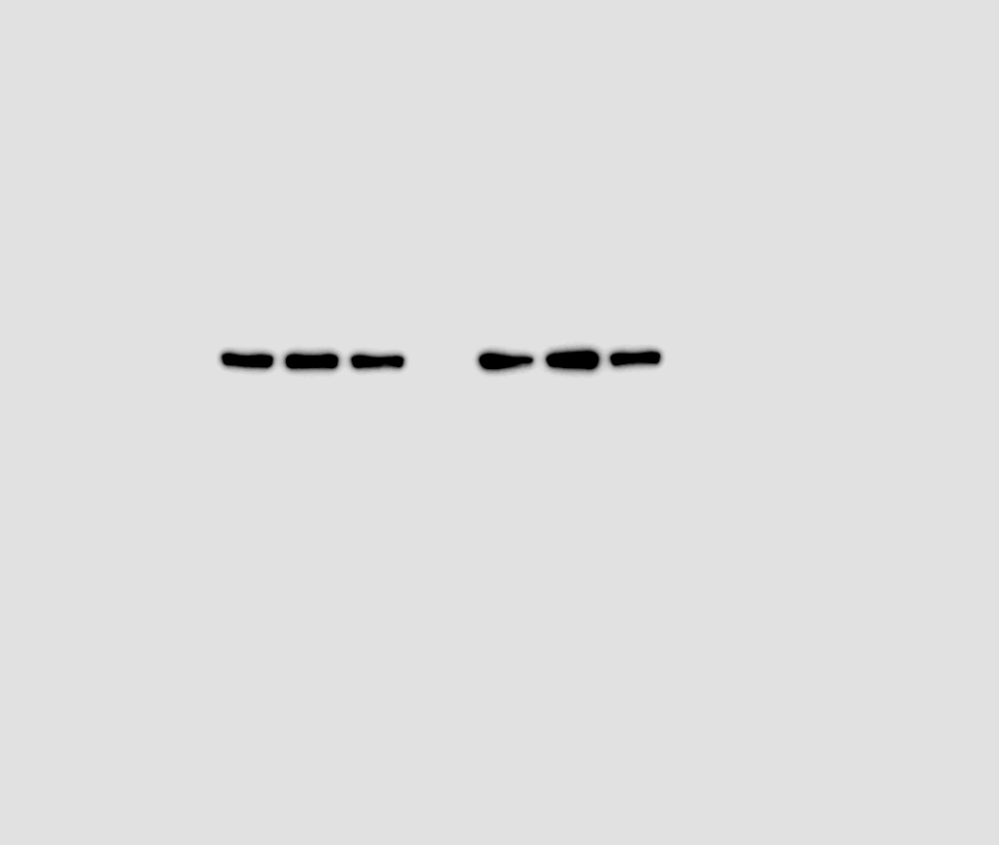

Supplement: Supplementary file 1 [file biomolecules-16-00856-s001.zip › Western Blot Files/Figure 8 (LXR Knockdown)/Figure 8I AR (post LXR knockdown)/Fig8I_MCF7TamR_Rep1,2_AR_RAW_restored.tif]

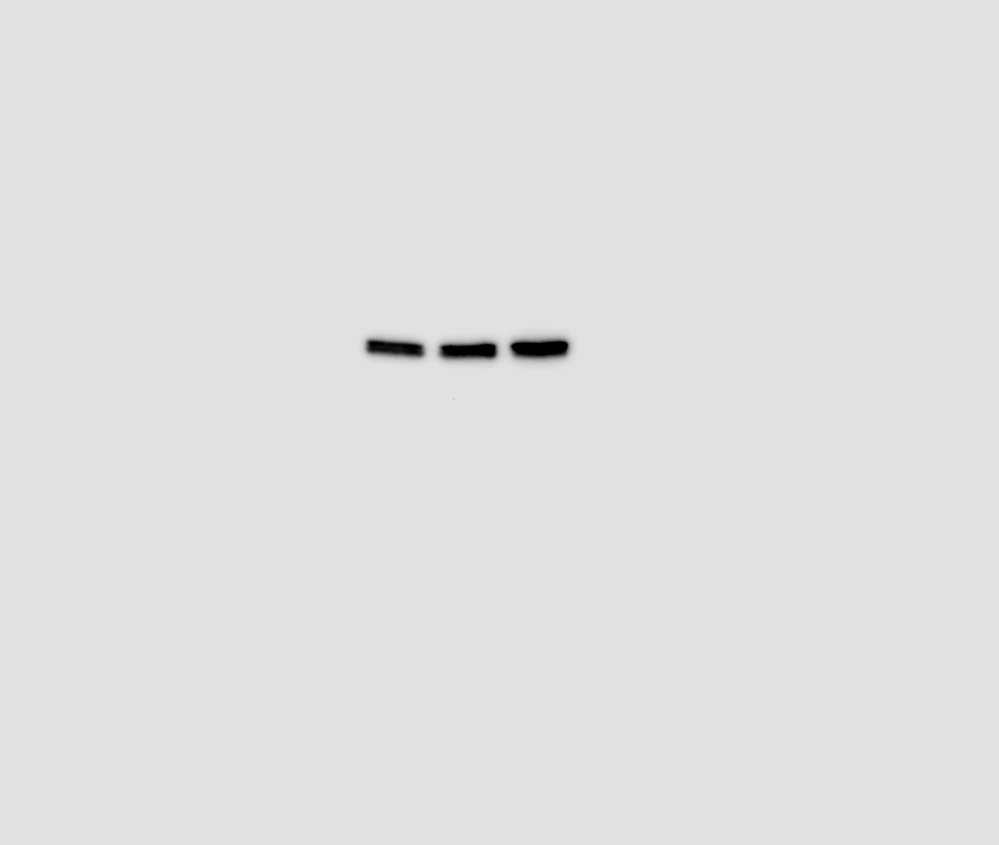

Supplement: Supplementary file 1 [file biomolecules-16-00856-s001.zip › Western Blot Files/Figure 8 (LXR Knockdown)/Figure 8I AR (post LXR knockdown)/Fig8I_MCF7TamR_Rep3_AR_RAW.tif]

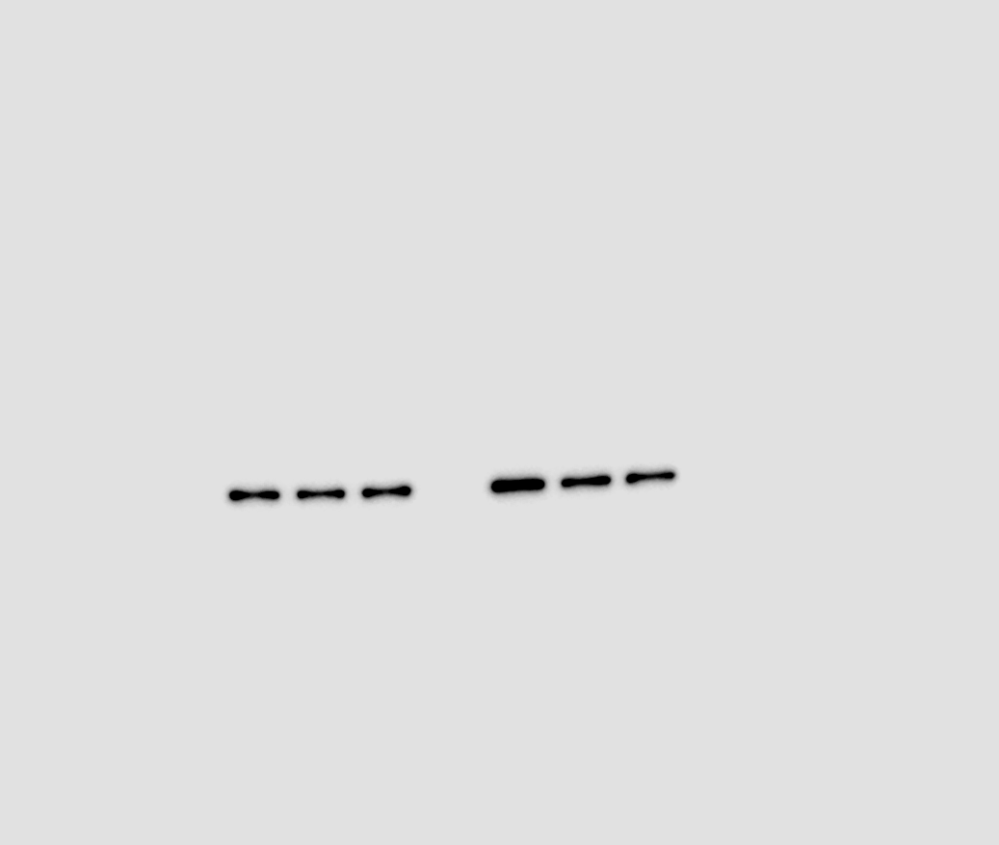

Supplement: Supplementary file 1 [file biomolecules-16-00856-s001.zip › Western Blot Files/Figure 8 (LXR Knockdown)/Figure 8L HER2 (post LXR knockdown)/Fig8L_MCF7TamR_Rep1,2_HER2_RAW.tif]

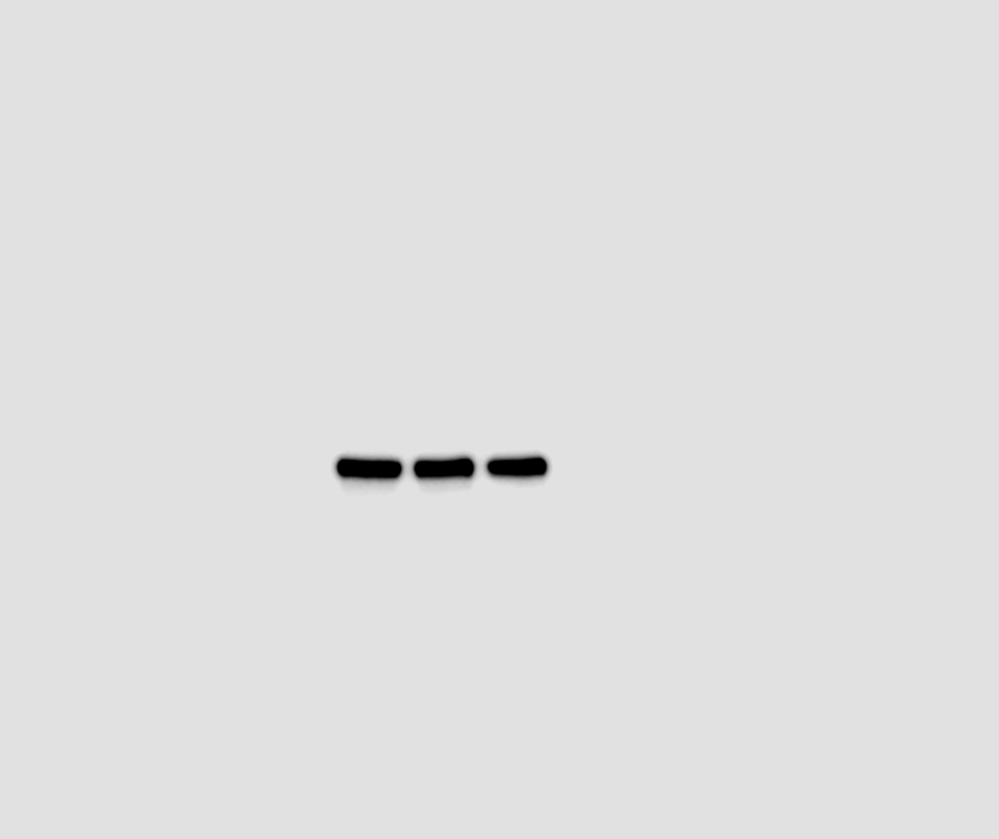

Supplement: Supplementary file 1 [file biomolecules-16-00856-s001.zip › Western Blot Files/Figure 8 (LXR Knockdown)/Figure 8L HER2 (post LXR knockdown)/Fig8L_MCF7TamR_Rep3_HER2_RAW.tif]
